# Diterpenoid Constituents of *Psiadiapunctulata* and Evaluation of Their Antimicrobial Activity

**DOI:** 10.1021/acs.jnatprod.1c01093

**Published:** 2022-06-24

**Authors:** Giuliana Donadio, Maria Giovanna Chini, Valentina Parisi, Francesca Mensitieri, Nicola Malafronte, Giuseppe Bifulco, Angela Bisio, Nunziatina De Tommasi, Ammar Bader

**Affiliations:** † Department of Pharmacy, University of Salerno, Via Giovanni Paolo II 132, 84084, Fisciano, Salerno, Italy; ‡ Department of Biosciences and Territory, University of Molise, Contrada Fonte Lappone, I-86090, Pesche, Isernia, Italy; § Ph.D. Program in Drug Discovery and Development, Department of Pharmacy, University of Salerno, Via Giovanni Paolo II 132, 84084, Fisciano, Salerno, Italy; ⊥ Department of Medicine, Surgery and Dentistry “Scuola Medica Salernitana”, University of Salerno, Via Salvador Allende, 84081, Baronissi, Italy; ∥ Department of Pharmacy, University of Genova, Viale Cembrano 4, 16148, Genova, Italy; ∇ Department of Pharmacognosy, Umm Al-Qura University, 21955 Makkah, Saudi Arabia

## Abstract

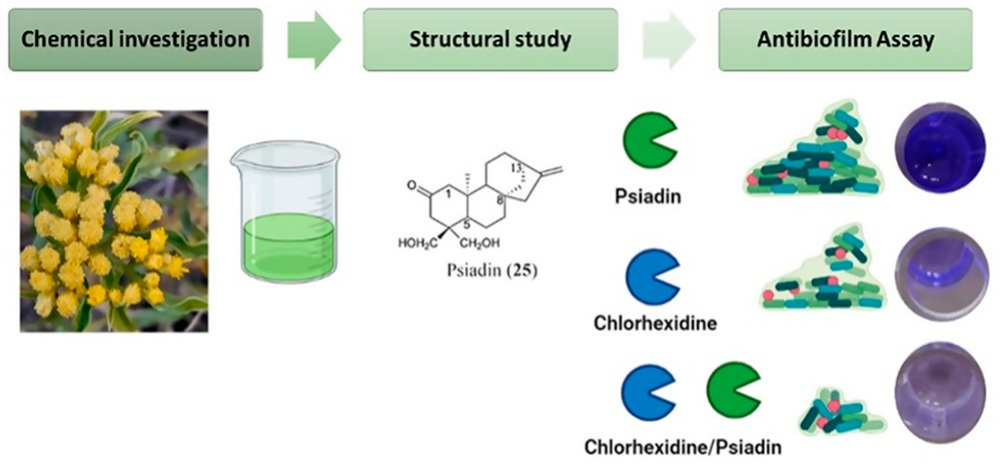

Sixteen diterpenes (**1**–**16**), along with 10 previously described compounds, including four flavonoids and six diterpenes, were isolated from the aerial parts of *Psiadia punctulata* growing in Saudi Arabia. The diterpene structures were elucidated using NMR spectroscopy and mass spectrometry data. Furthermore, a DFT/NMR procedure was used to suggest the relative configuration of several compounds. The labdane-derived skeletons, namely, *ent*-atisane, *ent*-beyerene, *ent*-trachylobane, and *ent*-kaurene, were identified. The extracts, fractions, and pure compounds were then tested against *Staphylococcus aureus*, *Streptococcus mutans*, *Treponema denticola*, and *Lactobacillus plantarum*. One diterpenoid, namely, psiadin, showed an additive effect with the antiseptic chlorhexidine, with a fractional inhibitory concentration index of less than 1. Additionally, psiadin showed a prospective inhibition activity for bacterial efflux pumps.

As part of an ongoing effort to conduct phytochemical studies for Arabian medicinal plants, the leaf exudate and the roots of *Psiadia punctulata* (DC.) Vatke collected from Saudi Arabia (mountains surrounding the Holey Makkah) were previously investigated to evaluate their antimicrobial and the cytotoxic potential.^1^*P. punctulata* was well known in ancient Arabic medicine as “Tobbag” and was mentioned by the Arabic poet Ta’abbata Sharran about 1500 years ago.^2^ In Saudi ethnomedicine, the leaves of this plant, which are rich in exudate, are simmered in hot water, and then the oily layer that is formed is collected and preserved in a recipient to be used as a wound disinfectant. The leaves are also burned as an insect repellent, and fresh leaves are warmed and applied locally to accelerate the healing of broken bones in humans and animals. The flowers of the plant are a good source for bee honey.^2^ The plant is also used in different ethnopharmacological systems. In South Africa, it is used for the treatment of asthma, chronic cough, fever, nasal congestion, pneumonia, sore throat, and tuberculosis.^3^ In Yemen, fresh leaves from the plant are tied around the affected area to treat musculoskeletal diseases, dislocation, and bone fractures.^4^ In East Africa, the plant is used against infectious and parasitic diseases such as bronchitis, scabies, and malaria.^5^ Previous phytochemical studies on the plant’s leaf exudate revealed the presence of different classes of metabolites, including diterpenoids (kaurane and trachylobane), flavonoids, and phenylpropanoids.^1,5^ The antimicrobial activity of the extracts and pure compounds of *Psiadia* spp.^1^ has been previously studied as well. To deepen the investigation on this species, in this work, the chemical composition and the antimicrobial potential of the extract and components of the leaves of *P. punctulata* were considered. The antimicrobial activity was tested against *Staphylococcus aureus*, *Streptococcus mutans*, *Treponema denticola*, and *Lactobacillus plantarum*. These nonpathogenic commensal oral strains are described to be crucial in the progression of dental caries and periodontal diseases, through biofilm development. Periodontitis and dental caries are two of the most common bacterial infections in humans. These diseases destroy the attachment of teeth and are considered very frequent in dental pathology.^6,7^ The minimal inhibitory concentration (MIC) values and the ability of psiadin to inhibit biofilm formation were investigated. Moreover, the possible synergic or additive activity of psiadin with chlorhexidine, a traditional dental antiseptic agent, was evaluated. Bacterial efflux pumps are determinants of antibiotic resistance; they allow microorganisms to regulate their internal environment by removing toxic substances, including antimicrobial agents, metabolites, and quorum-sensing signal molecules.^8^ The effect of psiadin with chlorhexidine on bacterial efflux pumps was then tested.

**Chart 1:**
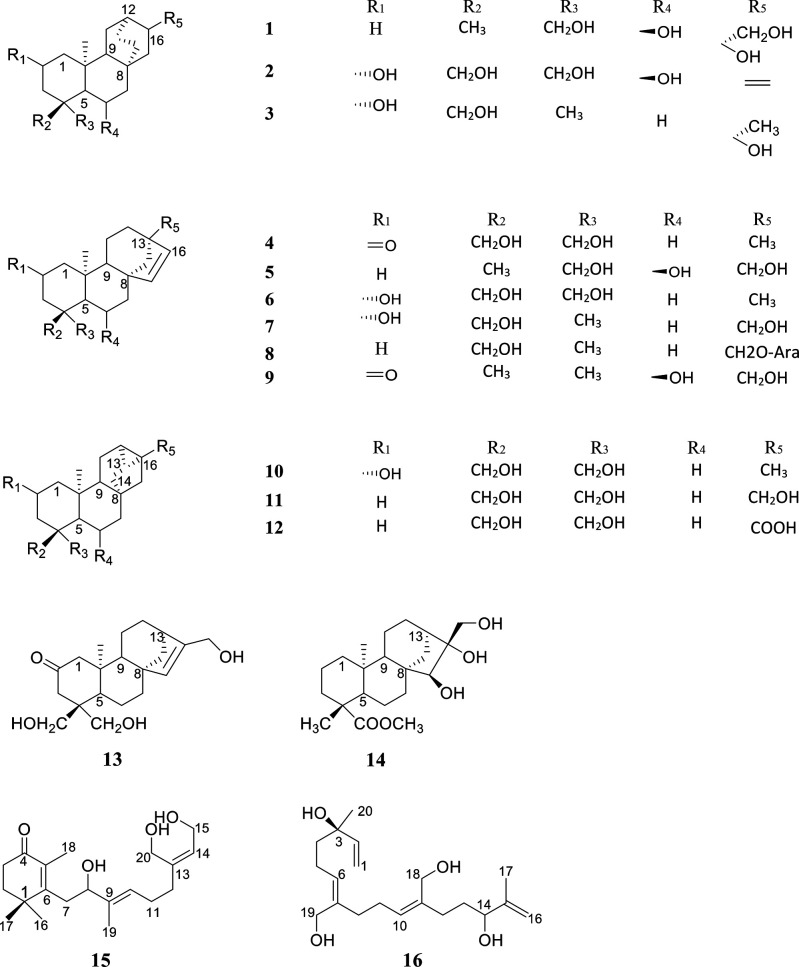


## Results and Discussion

Compound **1**, obtained as a colorless powder, was assigned the molecular formula C_20_H_34_O_4_ by its HRESIMS *m*/*z* of 361.2341 [M + Na]^+^, equating to four double-bond equivalents. The ^13^C NMR data (Table 1) showed 20 carbon signals, indicative of two methyls, eight methylenes, three methines, three quaternary carbons, two hydroxymethylenes, one hydroxymethine, and one nonprotonated carbon bearing a hydroxy group. The ^1^H NMR spectrum showed signals due to two hydroxymethylene groups at δ_H_ 3.37 (1H, d, *J* = 11.3) and 3.50 (1H, d, *J* = 11.3) and δ_H_ 3.46 (1H, d, *J* = 10.3) and 4.00 (1H, d, *J* = 10.3) and one hydroxymethine at δ_H_ 4.05 (1H, ddd, *J* = 14.0, 12.0, 3.9 Hz). A combination of 1D-TOCSY and ^1^H–^1^H COSY experiments provided evidence for the presence of the spin systems H-1/H-3, H-5/H-7, and H-9/H-14. The elucidation of all basic carbon skeletons from the above subunits was obtained by a series of ^1^*J*(HSQC) and ^3^*J*(HMBC) correlations, which also allowed the assignment of resonances in the ^13^C NMR spectrum to the pertinent carbon (Table 1). The NMR data suggested that **1** possessed the tetracyclic structure of *ent*-atisanes.^9^ HMBC correlations were observed between H_2_-19 and C-18, C-5, and C-4; H_3_-18 with C-5 and C-3; H-5 with C-9, C-4, C-20, and C-7; H_2_-17 with C-12, C-15, and C-16; H_2_-15 with C-9, C-8, and C-14; and H_2_-13 with C-12, C-14, C-15, C-16, and C-17. The relative configuration of C-8, C-12, and C-16 was suggested by calculating NMR properties at the quantum mechanical (QM) level, such as the chemical shifts (QM/NMR) for ^13^C and ^1^H NMR.^10,11^ This procedure, which was developed and optimized by us,^10^ consists of a multistep workflow, where the correct prediction of the stereochemical assignment of organic compounds is suggested by the most reliable correspondence between the experimental ^13^C and ^1^H NMR chemical shifts (related to the real isomer with unknown relative configuration) and the calculated ones (related to all the possible isomers with known relative configuration). Specifically, an extensive conformational search was carried out at the empirical level using Monte Carlo molecular mechanics (MCMM), low-mode conformational sampling (LMCS), and molecular dynamics (MD) simulations (see computational details, Experimental Section) for all possible diastereomers (**1a**–**d**). All the obtained conformers were then submitted to a geometry and energy optimization step based on density functional theory (DFT) at the MPW1PW91/6-31G(d) level of theory. Then, ^13^C and ^1^H NMR chemical shifts were predicted at the MPW1PW91/6-31G(d,p) level for **1a**–**d**, taking into account the Boltzmann distribution of the conformers for each stereoisomer obtained at the same level of theory. For all the DFT calculations, the integral equation formalism model (IEFPCM) was used for simulating MeOH as a solvent.^12,13^ Subsequently, the mean absolute error (MAE) values were used to compare the calculated and experimental values (see computational details, Experimental Section). Compound **1b** showed the lowest ^13^C and ^1^H MAE values (1.27 and 0.17 ppm, respectively), indicating 8R*,12S*,16S* as the relative configuration for **1**. To further confirm these findings, the DP4+ method was also employed,^14^ which is a powerful tool for assigning the correct stereochemical patterns for organic compounds. Again, the isomer **1b** showed the highest DP4+ probabilities (100.00%). Thus, the structure of **1** was defined as *ent*-atisan-6β,16β,17,19-tetraol.

**Table 1:** ^1^H and ^13^C NMR Spectroscopic Data for Compounds **1**, **2**, and **3**^a^

	**1**			**2**			**3**		
position	δ_C_, type	δ_H_	HMBC^c^	δ_C_, type	δ_H_	HMBC^c^	δ_C_, type	δ_H_	HMBC^c^
1	39.4, CH_2_	0.91 ddd (15.0, 13.0, 5.0); 1.54^b^	3, 5	48.1, CH_2_	1.18 dd (19.0;11.8) 1.71^b^	2, 5, 10, 20	45.0, CH_2_	1.36^b^; 1.67 dd (13.5; 4.5)	2, 5, 10, 20
2	17.8, CH_2_	1.34^b^; 1.56^b^		65.7, CH	3.98 m		67.0, CH	4.12 m	
3	39.7, CH_2_	1.12^b^; 1.56^b^		36.8, CH_2_	1.42 br t (12.5); 1.80 dd (14.0, 3.7)	1, 4, 5, 18, 19	41.0, CH_2_	1.46^b^; 1.80 dd (14.0; 4.2)	1, 2, 5, 18, 19
4	38.8, C			42.4, C			37.0, C		
5	61.5, CH	1.03^b^	4, 6, 7, 9, 20	53.7, CH	1.05 d (11.5)	4, 7, 18, 19, 20	48.4, CH	1.23	
6	68.2, CH	4.05 ddd (14.0; 12.0; 3.9)	4, 9	67.3, CH	4.01 ddd (14.0; 12.0; 3.9)		21.0, CH_2_	1.50^b^; 1.70^b^	
7	49.4, CH_2_	1.20^b^; 1.67^b^	5, 6, 8, 9, 14	45.0, CH_2_	1.36^b^; 1.70^b^		39.8, CH_2_	1.20 m; 1.37^b^	8, 14, 15
8	39.3, C			41.3, C			34.0, C		
9	52.0, CH	1.02^b^		53.3, CH	1.34^b^	4, 18, 19, 20	52.5, CH	1.32^b^	
10	38.3, C			41.1, C			38.5		
11	22.3, CH_2_	1.31^b^; 2.09 ddd (14.5; 11.0; 5.0)		26.0, CH_2_	1.58 m; 1.70^b^	8, 12, 16	19.6, CH_2_	1.40^b^; 1.45^b^	
12	32.8, CH	1.82 m		37.9, CH	2.27 m	8, 14, 15, 16, 17	37.7, CH	1.56 m	
13	25.0, CH_2_	1.26^b^; 1.65^b^	12, 14, 15, 16, 17	28.3, CH_2_	1.65^b^		24.0, CH_2_	1.28^b^; 2.10 br t (11.0)	9, 14
14	29.6, CH_2_	1.3^b^; 1.90 br t (12.8)		29.0, CH_2_	1.54 m; 1.69^b^	9, 10, 16	29.0, CH_2_	0.85 m; 1.90^b^	9
15	52.5, CH_2_	1.08^b^; 1.26^b^		49.0, CH_2_	2.00 d (15.5); 2.12 d (15.5)	7, 8, 14, 16, 17	58.2, CH_2_	1.24^b^; 1.29^b^	
16	75.0, C			152.8, C			72.0, C		
17	68.7, CH_2_	3.37 d (11.3); 3.50 d (11.3)	12, 15, 16	105.8, CH_2_	4.60 br s; 4.78 br s	12, 15, 16	30.0, CH_3_	1.27 s	13, 15, 16
18	31.0, CH_3_	1.19 s	3, 4, 5, 19	67.9, CH_2_	3.50 d (11.6); 3.91 d (11.6)	3, 4, 5, 19	70.0, CH_2_	3.10 d (11.5); 3.36 d (11.5)	3, 4, 5, 19
19	65.6, CH_2_	3.46 d (10.3); 4.00 d (10.3)	3, 4, 5, 18	65.3, CH_2_	3.67 d (11.6); 4.05 d (11.6)	3, 4, 5, 18	20.6, CH_3_	0.98 s	3, 4, 5, 18
20	16.0, CH_3_	1.09 s	1, 5, 9, 10	21.0, CH_3_	1.30 s	1, 5, 10	18.0, CH_3_	1.33 s	1, 5, 9, 10

a Spectra were recorded in methanol-*d*_4_, at 600 MHz (^1^H) and 150 MHz (^13^C); chemical shifts are given in ppm; *J* values are in parentheses and reported in Hz; assignments were confirmed by DQF-COSY, 1D-TOCSY, and HSQC experiments.

b Overlapped signal.

c HMBC correlations are from proton(s) stated to the indicated carbon.

Compound **2** was assigned the molecular formula C_20_H_32_O_4_ by its HRESIMS spectrum, which was acquired in the positive ion mode (*m*/*z* 337.2364 [M + H]^+^). This information, along with the ^13^C NMR data (Table 1), led to the determination of five indices of hydrogen deficiency and an *ent*-atiserene skeleton for **2**.^9^ The ^1^H and ^13^C NMR data (Table 1) of compound **2** showed several differences from **1**. Specifically, the ^1^H NMR spectrum (Table 1) showed signals due to one *exo*-methylene at δ_H_ 4.60 and 4.78 (2H, br s), two hydroxymethylene groups at δ_H_ 3.50 (1H, d, *J* = 11.6 Hz) and 3.91 (1H, d, *J* = 11.6 Hz) and δ_H_ 3.67 (1H, d, *J* = 11.6 Hz) and 4.05 (1H, d, *J* = 11.6 Hz), and two hydroxymethines at δ_H_ 3.98 (1H, m) and 4.01 (1H, ddd, *J* = 14.0, 12.0, 3.9 Hz). The HMBC correlations of H_2_-17/C-12 and H_2_-17/C-15 confirmed the presence of the *exo*-methylene group at C-17. ^1^H–^1^H COSY correlations of H_2_-1/H-2 and H-2/H_2_-3 as well as HMBC cross-peaks at H-1/C-2, H-1/C-5, and H-3/C-2 suggested that the second hydroxy group was located at C-2. The other hydroxy group was present at C-6 according to the COSY and HSQC data. The hydroxymethylenes at δ_C_ 67.9 and 65.3 were located at C-18 and C-19, respectively, as indicated by the proton and carbon chemical shifts of the A-ring and the HMBC correlations of H-3/C-18 and C-19 and H-5/C-18 and C-19. The relative configuration of **2** was obtained on the basis of the literature survey^9^ and ROESY data. The ROESY correlations of H-5/H-2 and H-9 and H-18 showed that these protons were on the same side. On the other hand, the correlations between H_3_-20/H-6 and H-19 allowed us to confirm the β orientation of OH at C-6. Thus, **2** was characterized as *ent*-atis-16(17)-en-2α,6β,18,19-tetraol.

The positive HRESIMS of compound **3**, obtained as an amorphous white powder, gave a sodiated molecular ion at *m*/*z* 345.2396 [M + Na]^+^ in accordance with the molecular formula C_20_H_34_O_3_, indicating an index of hydrogen deficiency of four. A comparison between the NMR data of **3** (Table 1) and those of *ent*-atisan-16α,18-diol^15^ indicated that both compounds had similar skeletons. Compound **3** showed an additional oxymethine group at δ_H_ 4.12 (1H, m), located at the C-2 position, which was confirmed by the ^1^H–^1^H COSY, HSQC, and HMBC correlations. The structure of compound **3** was then elucidated as *ent*-atisan-2α,16α,18-triol.

Compound **4**, obtained as a white powder, gave a molecular formula of C_20_H_30_O_3_ according to a [M + H]^+^ ion at *m*/*z* 319.2262 in its HRESIMS spectrum, indicating an index of hydrogen deficiency of six. The ^13^C NMR data (Table 2) revealed the presence of 20 carbon signals, indicative of two methyls, seven methylenes, four methines (two of which were sp^2^-hybridized), four quaternary carbons, one carbonyl carbon, and two hydroxymethylenes. The hydroxymethylenes at δ_H_ 3.60 (1H, d, *J* = 11.0 Hz) and 3.47 (1H, d, *J* = 11.0 Hz) and δ_H_ 3.58 (1H, d, *J* = 11.2 Hz) and 3.48 (1H, d, *J* = 11.2 Hz) were assigned to the C-18 and C-19 positions, respectively, based on the HMBC correlations of H_2_-3/C-18 and C-19 and H-5/C-18 and C-19. The oxo group (δ_C_ 215.6) was assigned to the C-2 position, which was corroborated by the HMBC correlations of H_2_-1/C-2 and H_2_-3/C-2. The ^1^H and ^13^C NMR data of **4** (Table 2) indicated a close structural similarity with *ent*-beyer-15-ene-18,19-diol.^16^ The only difference was in the presence of the keto carbonyl at C-2. As reported above, the QM/NMR approach was used to tentatively assign the configuration to C-8 and C-13 of compound **4**.^10,11^ Considering the experimental and literature data, in silico studies for the two possible diastereomers (**4a**,**b**) were performed. Briefly, the multistep computational procedure consists of four fundamental steps: (a) the conformational search and preliminary geometry optimization of all the significantly populated conformers of the compound diastereomers under examination; (b) the final geometry optimization of all the species at the QM level (MPW1PW91/6-31G(d) level of theory, using the IEFPCM model for simulating MeOH as solvent); (c) QM calculations of the ^13^C/^1^H NMR chemical shift of all the so-obtained structures at the QM level (MPW1PW91/6-31G(d,p), using the IEFPCM model for simulating MeOH as a solvent); (d) a comparison between the experimental and calculated Boltzmann-averaged experimental data (the ^13^C/^1^H NMR chemical shift for all the species, using statistical parameters to find the best-fitting model by utilizing the MAE values). Compound **4a** showed the lowest ^13^C and ^1^H MAE values (2.50 and 0.21 ppm, respectively), indicating 8R*,13S* as the relative configuration for **4**. To further confirm these findings, the DP4+ method was also employed,^14^ where the isomer **4a** showed the highest DP4+ probabilities (100.00%). Accordingly, compound **4** was identified as *ent*-18,19-dihydroxybeyer-15-en-2-one.

**Table 2:** ^1^H and ^13^C NMR Spectroscopic Data for Compounds **4, 5** and **6**^a^

	**4**			**5**			**6**		
position	δ_C_, type	δ_H_	HMBC^c^	δ_C_, type	δ_H_	HMBC^c^	δ_C_, type	δ_H_	HMBC^c^
1	53.8, CH_2_	2.12 d (13.5); 2.26 dd (13.5; 1.6)	2, 5, 10, 20	40.9, CH_2_	1.00 br dt (15.0; 13.0; 4.0) 1.65^b^	9, 10, 20	46.7, CH_2_	1.40^b^; 1.67^b^	2, 10, 20
2	215.6, C			19.2, CH_2_	1.31^b^; 1.64^b^		67.8 CH	4.11 m	4, 10
3	44.8, CH_2_	2.44 dd (14.0; 1.6); 2.52 d (14.0)	2, 5, 18, 19	39.6, CH_2_	1.11^b^; 1.62^b^		36.0, CH_2_	1.67^b^; 1.76^b^	1, 2, 4, 5, 18, 19
4	46.8, C			40.0, C			42.0, C		
5	47.6, CH	1.98 dd (12.3; 1.8)	4, 6, 7, 10, 18, 19, 20	61.9, CH	1.13 d (13.0)	4, 6, 7, 9, 18, 19, 20	48.6, CH	1.34^b^	
6	20.6, CH_2_	1.50^b^; 1.74 m		69.6, CH	4.09 ddd (15.0; 12.0; 4.5)		21.0, CH_2_	1.53^b^; 1.65^b^	7
7	36.9, CH_2_	1.48^b^; 1.66 d t (13.0; 6.0; 2.5)	5, 9	47.0, CH_2_	1.50 br t (12.0); 1.89 dd (12.7; 3.86)	5, 6, 8, 9, 15	38.4, CH_2_	1.37^b^; 1.60^b^	4, 8, 15
8	43.3, C			49.0, C			49.2, C		
9	52.4, CH	1.36^b^		54.0, CH	1.10^b^		55.0, CH	1.07^b^	8, 10, 14, 20
10	42.2, C			40.2, C			36.9, C		
11	20.0, CH_2_	1.50^b^; 1.56 ddd (16.0; 13.0; 3.5)	10	21.1 CH_2_	1.27 br t (7.0); 1.63^b^	9, 12	20.8, CH_2_	1.48^b^; 1.78 br dd (13.0; 7.0)	
12	33.0, CH_2_	1.37^b^; 1.37^b^		28.6, CH_2_	1.33^b^; 1.42 m	11, 13, 14, 16	33.5, CH_2_	1.28^b^; 1.36^b^	
13	48.9, C			51.0, C			43.4, C		
14	61.3, CH_2_	1.12 d (10.0); 1.51^b^	8, 9, 12, 13, 15, 16	57.2, CH_2_	1.09^b^; 1.65^b^		62.0, CH_2_	1.06^b^; 1.44 m	
15	134.9, CH	5.74 d (5.9)	8, 13, 14, 16	137.3, CH	5.82 d (6.0)	13, 14, 16	135.9, CH	5.75 d (6.0)	8, 13, 14, 16
16	137.0, CH	5.50 d (5.9)	8, 13, 14, 15	134.3, CH	5.65 d (6.0)	8, 14, 15	137.5, CH	5.48 d (6.0)	8, 13, 14, 15
17	24.8, CH_3_	1.04^b^	8, 12, 14, 16	68.9, CH_2_	3.41 d (11.5); 3.46 d (11.5)	8, 12, 14, 16	25.6, CH_3_	1.00 s	12, 14, 16
18	66.6, CH_2_	3.60 d (11.0); 3.47 d (11.0)	3, 4, 5, 19	32.0, CH_3_	1.21 s	4, 5, 19	69.3, CH_2_	3.51 d (10.8); 3.55 d (10.8)	3, 4, 5, 19
19	63.4, CH_2_	3.58 d (11.2); 3.48 d (11.2)	3, 4, 5, 18	67.8, CH_2_	3.48 d (10.7); 4.01 d (10.7)	4, 5, 18	66.0, CH_2_	3.66 d (11.5); 3.91 d (11.5)	3, 4, 5, 18
20	16.8, CH_3_	0.84 s	1, 5, 9, 10	17.4, CH_3_	0.89 s	1, 5, 9, 10	20.0, CH_3_	1.06 s	1, 5, 9, 10

a Spectra were recorded in methanol-*d*_4_, at 600 MHz (^1^H) and 150 MHz (^13^C); chemical shifts are given in ppm; *J* values are in parentheses and reported in Hz; assignments were confirmed by DQF-COSY, 1D-TOCSY, and HSQC experiments.

b Overlapped signal.

c HMBC correlations are from proton(s) stated to the indicated carbon.

Compound **5**, obtained as an amorphous white powder, showed a molecular formula of C_20_H_32_O_3_ according to a [M + Na]^+^ ion at *m*/*z* 343.2238, which is indicative of five indices of hydrogen deficiency. The NMR data (Table 2) also led to the determination of a *ent*-beyerene^17^ scaffold for **5**. The NMR data of **5** closely resembled those of *ent*-17,19-dihydroxybeyer-15,16-ene^16^ other than the presence of a hydroxymethine at C-6. The signal at δ_H_ 4.09 (Table 2) was assigned to H-6 by the ^1^H–^1^H COSY and HMBC correlations. Its β orientation was defined through the analysis of the H-6 coupling constants [δ_H_ 4.09 ddd (*J* = 15.0, 12.0, 4.5 Hz)], which are typical of an axial proton. The ROESY correlations of H-6/H_3_-20, H-6/H_2_-19, and H_3_-20/H_2_-19 confirmed the relative configuration. Therefore, the structure of **5** was identified as *ent*-beyer-15-ene-6β,17,19-triol.

Compound **6** was isolated as an amorphous white powder. Its molecular formula was determined as C_20_H_32_O_3_ by the positive HRESIMS signal at *m*/*z* 321.2417 [M + H]^+^ and 343.2239 [M + Na]^+^, and it accounted for five indices of hydrogen deficiency. The ^1^H and ^13^C NMR data (Table 2) of **6** were nearly superimposable with those of **4**, with the exception of the lack of the signal of the oxo group at C-2, which was replaced in **6** by a hydroxymethine (δ_C_ 67.8; δ_H_ 4.11, m). The 2α-OH orientation was deduced by the ROESY correlations H-2/H-5 and data from the literature.^18^ The same procedure that was applied for compounds **2** and **5** was used to suggest the relative configuration of compound **6** at C-8 and C-13. Further, in this case, the two possible diastereomers (**6a** and **6b**) and the combined evaluation of the ^13^C and ^1^H MAE values (1.50 and 0.25 ppm, respectively), together with the analysis of the DP4+ probability values (100.00%), led to the assigning of 8R*,13S* as the relative configuration for **6**. Compound **6** was thus established as *ent*-beyer-15-en-2α,18,19-triol.

Compound **7** was obtained as an amorphous white powder. Its HRESIMS spectrum exhibited a molecular ion at *m*/*z* 321.2422 [M + H]^+^ in accordance with the molecular formula C_20_H_32_O_3_, and it required five degrees of hydrogen deficiency. The ^1^H NMR spectrum (Table 3) showed signals due to two hydroxymethilene groups at δ_H_ 3.42 (1H, d, *J* = 11.5 Hz) and 3.45 (1H, d, *J* = 11.5 Hz) and δ_H_ 3.09 (1H, d, *J* = 11.0 Hz) and 3.37 (1H, d, *J* = 11.0 Hz), which were assigned to the C-17 and C-18 positions, respectively, based on the HMBC correlations H_2_-17/C-12, H_2_-17/C-14, and H_2_-18/C-19 and H_2_-18/C-5, respectively. The hydroxy group at C-2 was confirmed by the HMBC correlations H_2_-1/C-2, H-2/C-10, and H_2_-3/C-2. The NMR data of **7** closely resembled those of *ent*-beyer-15-ene-17,19-diol,^16^ with the exception of the hydroxy group at C-2 and the hydroxy group being located at C-18 instead of C-19. The ROESY correlation of H-2 with H_2_-18 implied that OH-2 was α-oriented. Following the same procedures reported above, the diastereomers of **7a** (8R*,13R*) showed a better fit with the experimental data (0.89 and 0.14 ppm as the ^13^C and ^1^H MAE values, respectively; DP4+ probability value of 100.00%). Therefore, compound **7** was determined to be *ent*-beyer-15-en-2α,17,18-triol.

**Table 3:** ^1^H and ^13^C NMR Spectroscopic Data for Compounds **7**, **8**, and **9**^a^

	**7**			**8**			**9**		
position	δ_C_, type	δ_H_	HMBC^c^	δ_C_, type	δ_H_	HMBC^c^	δ_C_, type	δ_H_	HMBC^c^
1	46.4, CH_2_	1.42^b^; 1.69^b^	2, 5, 10, 20	40.0, CH_2_	0.90 ddd (16.0; 13.0; 4.0); 1.64^b^		54.6, CH_2_	2.17 d (13.6); 2.22^b^	2, 5, 10, 16
2	68.0, CH	4.13 sxt (11.9; 10.2; 6.0)	3, 10	19.0, CH_2_	1.44^b^; 1.63^b^		215.4, C		
3	41.0, CH_2_	1.47^b^; 1.81 dd (14.0; 4.0)	2, 4, 18, 19	36.4, CH_2_	1.27^b^; 1.49^b^		57.0, CH_2_	2.22^b^; 2.38 d (13.5)	2, 4, 18, 19
4	38.5, C			37.9, C			40.4, C		
5	48.0, CH	1.28 dd (10.5; 5.0)	1, 4, 18, 19	49.8, CH	1.28^b^	4, 9, 10, 19, 20	60.2, CH	1.51 d (11.0)	3, 6, 9, 10, 16, 18, 19
6	21.2, CH_2_	1.49^b^; 1.54^b^		20.8, CH_2_	1.44^b^; 1.60^b^		68.7, CH	4.01 ddd (15.3; 11.0; 4.4)	
7	37.8, CH_2_	1.47^b^; 1.66^b^		38.6, CH_2_	1.48; 1.64^b^		48.3, CH_2_	1.61 br t (12.0); 1.98 dd (13.0; 5.0)	5, 6, 9, 15
8	51.2, C			49.7, C			45.0, C		
9	55.4, CH	1.12 dd (10.0; 4.0)		54.6, CH	1.12 dd (9.4)		53.0, CH	1.35^b^	
10	38.3, C			38.2, C			42.0, C		
11	21.1, CH_2_	1.45^b^; 1.66^b^		20.8, CH_2_	1.33^b^; 1.52^b^		21.0, CH_2_	1.36^b^; 1.56 m	
12	28.9, CH_2_	1.33 m; 1.44^b^		29.4, CH_2_	1.38^b^; 1.51^b^	9, 13, 16	28.5, CH_2_	1.37^b^; 1.46 m	
13	49.7, C			48.0, C			48.9, C		
14	57.2, CH_2_	1.03 d (9.7); 1.59^b^	9, 12, 15, 16	57.6, CH_2_	1.06 d (10.5); 1.62^b^	8, 12, 13, 15	57.8, CH_2_	1.14 br d (10.0); 1.71 dd (10.3; 3.0)	9, 12, 16
15	137.2, CH	5.84 d (5.5)	9, 14, 16	137.2, CH	5.81 d (5.7)	13, 14, 16	136.5, CH	5.82 d (5.8)	13, 16, 17
16	133.5, CH	5.63 d (5.5)	8, 14, 15	133.6, CH	5.69 d (5.7)	13, 15	134.9, CH	5.69 d (5.8)	15
17	69.0, CH_2_	3.42 d (11.5); 3.45 d (11.5)	8, 12, 14, 16	76.6, CH_2_	3.32 d (9.5); 3.77 d (9.5)	12, 13, 14, 16, Ara-1	68.5, CH_2_	3.41 d (11.0); 3.50 d (11.0)	12, 15, 16
18	71.6, CH_2_	3.09 d (11.0); 3.37 d (11.0)	3, 4, 5, 19	72.1, CH_2_	3.02 d (11.5); 3.37 d (11.5)	4, 5, 19	37.2, CH_3_	1.33 s	3, 4, 5, 19
19	20.7, CH_3_	0.99 s	3, 4, 5, 18	18.3, CH_3_	0.79 s	3, 4, 5, 19	24.8, CH_3_	1.19 s	3, 4, 5,18
20	19.5, CH_3_	1.10 s		16.2, CH_3_	0.85 s	1, 5, 9, 10	17.6, CH_3_	0.89 s	5, 9, 10
Ara-1				105.0, CH	4.20 d (7.3)	17			
2				72.3, CH	3.60 br t (8.9)				
3				74.2, CH	3.54^b^				
4				69.3, CH	3.83 m				
5				66.3; CH_2_	3.54^b^; 3.86 br d (4.0)	Ara-1, Ara-3, Ara-4			

a Spectra were recorded in methanol-*d*_4_, at 600 MHz (^1^H) and 150 MHz (^13^C); chemical shifts are given in ppm; *J* values are in parentheses and reported in Hz; assignments were confirmed by DQF-COSY, 1D-TOCSY, and HSQC experiments.

b Overlapped signal.

c HMBC correlations are from proton(s) stated to the indicated carbon.

The molecular formula of **8** was determined as C_25_H_40_O_6_ by HRESIMS, showing a sodium adduct ion at *m*/*z* 459.2705 [M + Na]^+^ and a protonated molecular ion at *m*/*z* 437.2888 [M + H]^+^. The ^13^C NMR spectrum (Table 3) showed the presence of 20 carbon atoms that were attributable to a aglycon moiety and five that were attributable to the saccharide portion, establishing the presence of a monosaccharide unit. In the HRESIMS data, the fragment at *m*/*z* 305.2469 [M + H – 132]^+^ suggested the presence of a pentose. The aglycone of **8** showed close similarity to *ent*-beyer-15-ene-17,19-diol,^16^ with the difference being that the hydroxy group was located at C-18 instead of C-19. The structure of the sugar moiety was deduced by the ^1^H–^1^H COSY, HSQC, and HMBC experiments, leading to the recognition of an α-arabino-pyranosyl moiety.^19^ Direct evidence of the sugar linkage at C-17 was derived from the HMBC correlations between H-1_Ara_ at δ_H_ 4.20 (1H, d, *J* = 7.3 Hz) and C-17 at δ_C_ 76.6. Consequently, **8** was defined as *ent*-17,18-dihydroxybeyer-15-en-17-arabinopyranoside.

The HRESIMS spectrum of **9** showed a molecular composition of C_20_H_30_O_3_ (*m*/*z* 319.2260 [M + H^+^]^+^) and involved six indices of hydrogen deficiency. Two of these could be accounted for (one carbonyl group and one double bound), and the lack of other unsaturated carbons implied a tetracyclic structure. The ^1^H NMR spectrum (Table 3) displayed the characteristic signals of three methyl singlets, i.e. δ_H_ 0.89 (3H, s), 1.19 (3H, s), and 1.33 (3H, s); two diastereotopic protons of an oxygenated methylene, i.e., δ_H_ 3.41 (1H, d, *J* = 11.0) and 3.50 (1H, d, *J* = 11.0); and two sp^2^ protons at δ_H_ 5.69 (1H, d, *J* = 5.8 Hz) and 5.82 (1H, d, *J* = 5.8 Hz). The placement of the carbonyl group at C-2 was deduced by the following observations: The chemical shifts of C-1 and C-3 were observed at 54.6 and 57.0 ppm, respectively, which were shifted downfield compared to the unsubstituted beyerenes (Table 3).^16^ These assignments were confirmed by the HMBC correlations from H_2_-1 to C-2, C-3, and C-10 and from C-20 and H_2_-3 to C-4, C-18, and C-19. Key HMBC correlations from H_2_-17 to C-13 and C-16 and from H-15 to C-9, C-14, and C-16 completed the substitution pattern with a hydroxy group at C-17. Thus, **9** was defined as *ent*-6β,17-dihydroxybeyer-15-en-2-one.

Compound **10** was obtained as an amorphous white powder. The HRESIMS showed an ion peak at *m*/*z* 321.2409 [M + H]^+^, which is consistent with a molecular formula of C_20_H_32_O_3_ and hydrogen deficiency of 5. The ^1^H and ^13^C NMR spectra of **10** (Table 4) displayed resonances attributable to a trachylobane skeleton.^1,5,20^ A comparison of its NMR spectra with those of *ent*-trachyloban-18,19-dihydroxy-2-one^1,20^ revealed differences in the signal due to the oxo group at C-2, replaced by an OH group. The relative stereochemistry was inferred from data from the literature and NOE data.^21^ Upon irradiation of H-2 (δ_H_ 4.09) in the 1D NOESY experiment, a NOE dipolar interaction with H-5 (δ_H_ 1.25) and H-9 (δ_H_ 1.24) was observed, which allowed us to assign the α orientation of OH-2. Therefore, compound **10** was found to be *ent*-trachylobane-2α,18,19-triol.

**Table 4:** ^1^H and ^13^C NMR Spectroscopic Data for Compounds **10**, **11**, and **12**^a^

	**10**			**11**			**12**		
position	δ_C_, type	δ_H_	HMBC^c^	δ_C_, type	δ_H_	HMBC^c^	δ_C_, type	δ_H_	HMBC^c^
1	46.3, CH_2_	1.29 m; 1.61^b^	2, 3, 5, 20	39.6, CH_2_	0.82 br d (12.0); 1.46^b^		40.0, CH_2_	0.81 ddd (15.0, 13.0, 3.8); 1.56 br d (13.4)	3, 5, 20
2	67.6, CH	4.09 m	4, 10	18.0, CH_2_	1.64^b^; 1.37^b^		20.8, CH_2_	1.36^b^; 1.62^b^	
3	36.2, CH_2_	1.64^b^; 1.73 dd (14.7; 7.4)	1, 4, 18	30.0, CH_2_	1.29^b^; 1.65^b^		30.6, CH_2_	1.32^b^; 1.72 m	1, 5
4	39.5, C			42.4, C			45.4, C		
5	48.3, CH	1.25^b^		50.1, CH	1.24^b^		50.8, CH	1.25^b^	
6	21.5, CH_2_	1.42^b^; 1.62^b^		19.0, CH_2_	1.43^b^; 1.50^b^		18.3, CH_2_	1.43^b^; 1.63^b^	
7	39.7, CH_2_	1.41^b^; 1.42^b^		39.0, CH_2_	1.44^b^; 1.56^b^		39.9, CH_2_	1.46^b^; 1.48^b^	
8	40.4, C			40.8, C			41.5, C		
9	54.8, CH	1.24^b^		54.0, CH	1.28^b^		54.1, CH	1.25^b^	
10	39.0, C			37.8, C			39.3, C		
11	20.6, CH_2_	1.83 ddd (14.5; 7.5; 2.3); 1.99 ddd (14.5; 11.2; 2.7)	9, 10, 16	20.5, CH_2_	1.73^b^; 1.97 ddd (14.0; 11.0; 2.5)	8, 9, 12, 13	21.0, CH_2_	1.80^b^; 2.00 ddd (14.0, 12.0, 3.3)	8, 9, 12, 14
12	21.8, CH	0.63 br d (8.4)	8, 16	19.0, CH	0.84^b^		25.0, CH	1.64^b^	
13	25.2, CH	0.85 dd (8.0; 3.2)		22.5, CH	1.08 dd (8.4; 3.0)	8, 14	30.6, CH	1.43^b^	
14	34.0, CH_2_	1.17 m; 2.13 br d (12.0)	8, 12, 15	34.0, CH_2_	1.17^b^; 2.15 d (11.2)	8, 9, 12, 13, 15, 16	33.5, CH_2_	1.31^b^; 2.20 br d (12.0)	9, 13, 15
15	51.4, CH_2_	1.28 d (11.5); 1.39 d (11.5)		46.4, CH_2_	1.41 d (12.0); 1.56 d (12.0)		43.4, CH_2_	1.49^b^; 1.82^b^	
16	21.8, CH			29.0, C			30.0, C		
17	20.6, CH_3_	1.15 s	13, 15, 16	68.8, CH_2_	3.54 br s; 3.55 br s	12, 13, 15, 16	180.0, C		
18	68.8, CH_2_	3.50 d (10.0); 3.54 d (10.0)	3, 4, 5, 19	68.4, CH_2_	3.49 d (1.6); 3.52 d (1.6)	3, 4, 5, 19	64.0, CH_2_	3.52 d (11.0); 3.78 d (11.0)	3, 4, 5, 19
19	66.0, CH_2_	3.66 d (10.8); 3.89 d (10.8)	3, 4, 5, 18	63.0, CH_2_	3.52 d (10.7); 3.81 d (10.7)	3, 4, 5, 18	69.4, CH_2_	3.53 d (11.2); 3.49 d (11.2)	3, 4, 5, 18
20	18.7, CH_3_	1.30 s	1, 5, 9, 10	15.6, CH_3_	1.01 s	1, 5, 9, 10	15.7, CH_3_	1.00	1, 5, 9, 10

a Spectra were recorded in methanol-*d*_4_, at 600 MHz (^1^H) and 150 MHz (^13^C); chemical shifts are given in ppm; *J* values are in parentheses and reported in Hz; assignments were confirmed by DQF-COSY, 1D-TOCSY, and HSQC experiments.

b Overlapped signal.

c HMBC correlations are from proton(s) stated to the indicated carbon.

Compound **11**, an amorphous white powder, had a molecular formula of C_20_H_32_O_3_ based on its HRESIMS (*m*/*z* 321.2417 [M + H]^+^) and the ^1^H and ^13^C NMR data (Table 4), suggesting five degrees of hydrogen deficiency. The ^1^H and ^13^C NMR data were very similar to those of *ent*-trachyloban-17,18,19-trihydroxy-2-one.^1^ The most remarkable difference was the substitution of the 2-oxo group in *ent*-trachyloban-17,18,19-trihydroxy-2-one with a methylene group in **11** (δ_C_ 18.0; δ_H_ 1.64, 1.37). The structure of **11** was thus assigned as *ent*-trachylobane-17,18,19-triol.

Compound **12** was isolated as an amorphous white powder. Its molecular formula was determined as C_20_H_30_O_4_ based on its HRESIMS (*m*/*z* 335.2206 [M + H]^+^) and the ^13^C NMR data (Table 4). The basic skeleton of *ent*-trachylobane-18,19-diol was recognized in **12** by comparing it with the data reported in previous literature^21^ and those of compound **11**. The only difference was the presence of a carboxyl group at C-17. Thus, **12** was elucidated as *ent*-18,19-trihydroxytrachyloban-17-oic acid.

Compound **13** was obtained as an amorphous white powder. The HRESIMS of **13** revealed a molecular ion at *m*/*z* 335.2212 [M + H]^+^, which is consistent with the molecular formula C_20_H_30_O_4_ and six indices of hydrogen deficiency. The ^13^C NMR data (Table 5) displayed 20 signals comprising one methyl, seven methylenes, four methines (one sp^2^), four quaternary carbons (one sp^2^), three hydroxymethylenes, and one carbonyl group. The analysis of the ^13^C NMR and ^1^H NMR data (Table 5) suggested an *ent*-kaurane nucleus.^22^ A comparison of the NMR data of **13** with those of *ent*-kaur-16(17)-en-18,19-dihydroxy-2-one (psiadin)^23^ indicated that **13** is a psiadin derivative. Particularly, the proton and carbon chemical shifts of the rings A and B resonated at almost the same frequencies as the corresponding signals in psiadin, while the NMR signals of rings C and D were observed at somewhat different chemical shift values. The NMR spectra of **13** showed the presence of signals at δ_H_ 5.39, δ_C_ 135.9 and δ_H_ 4.14, δ_C_ 61.0. The 1D TOCSY and ^1^H–^1^H COSY experiments provided evidence of the spin systems H-9/H-14 and H-15/H-17. Furthermore, the HMBC correlations of H_2_-14 with C-9, C-12, C-15, and C-16, H-15 with C-9, C-13, and C-17, and H_2_-17 with C-13, C-15, and C-16 established the presence of a Δ^15,16^ double bond and C-17 hydroxymethylene. Hence, the structure of **13** was established as *ent*-17,18,19-trihydroxykaur-15-en-2-one.

**Table 5:** ^1^H and ^13^C NMR Spectroscopic Data for Compounds **13**, **14**, **15**, and **16**^a^

	**13**			**14**			**15**			**16**		
position	δ_C_, type	δ_H_	HMBC^c^	δ_C_, type	δ_H_	HMBC^c^	δ_C_, type	δ_H_	HMBC^c^	δ_C_, type	δ_H_	HMBC^c^
1	56.7, CH_2_	2.01 d (13.0); 2.53 d (13.0)	2, 3, 5, 10, 20	42.5, CH_2_	0.91^b^; 1.87^b^	2, 9, 10, 20	36.0, C			112.0, CH_2_	5.06 dd (11.0; 1.6); 5.24 dd (17.0; 1.6)	3
2	215.8, C			20.2, CH_2_	1.39^b^; 1.88^b^		38.0, CH_2_	1.86^b^; 2.51^b^	1, 3, 4, 6, 16	146.2, CH	5.94 dd (17.0; 11.0)	3
3	45.3, CH_2_	2.47 d (15.0); 2.50 d (15.0)	5, 18, 19	36.7, CH_2_	1.64^b^; 1.80^b^		34.0, CH_2_	2.51^b^		72.0, C		
4	44.6, C			41.0, C			201.0, C			43.2, CH_2_	1.56 m	12, 14
5	49.5, CH	1.92 br d (12.0)	4, 9, 18, 19	58.0, CH	1.13^b^	3, 4, 6, 8, 9, 10, 19, 20	132.0, C			22.9, CH_2_	2.15 m	4, 6, 7
6	20.5, CH_2_	1.49^b^; 1.57^b^		22.4, CH_2_	1.72^b^; 1.92^b^	3, 5, 10	165.0, C			128.6, CH	5.37 m	8, 19
7	39.8, CH_2_	1.60 d t (12.0; 6.0; 2.5); 1.77^b^	5, 14	37.0, CH_2_	1.63^b^; 1.80^b^		38.0, CH_2_	1.84^b^; 2.65 dd (14.0, 8.0)	1, 4, 5, 6, 9	138.0, C		
8	48.0, C			52.0, C			77.0, CH	4.28 dd (7.0, 5.2)	10, 19	34.4, CH_2_	1.69 m	
9	48.4, CH	1.35 br d (9.5)	1, 5, 10, 11, 12, 14	56.0, CH	1.11^a^		139.0, C			27.0, CH_2_	2.26 m	
10	44.4, C			40.8, C			126.0, CH	5.42 m	11	128.6, CH	5.37 m	8, 18
11	20.0, CH_2_	1.73^b^; 1.76^b^		19.9, CH_2_	1.49^b^; 1.51^b^		27.0, CH_2_	2.24^b^		136.2, C		
12	26.2, CH_2_	1.55^b^; 1.57^b^		26.8, CH_2_	1.36 ddd (16.0; 14.0; 4.0); 1.60^b^	8, 9, 11, 14, 1	35.0, CH_2_	2.24^b^		27.0, CH_2_	2.25 m	
13	41.4, CH	2.58 m		44.0, CH	2.09 m	12, 15	141.1, C			43.0, CH_2_	1.69 m	
14	42.5, CH_2_	2.11 br d (14.5); 2.43 br d (14.5)	12, 13, 15, 16	39.0, CH_2_	1.62^b^; 1.80^b^		127.4, C	5.51 br t (6.6)	12, 15	76.0, CH	4.03 br t (7.0)	
15	135.9, CH	5.39 s	8, 9, 14, 17	83.1, CH	3.37 br s	9, 13, 14, 17	58.5, CH_2_	4.18 d (6.6)	13, 14	147.0, C		
16	147.9, CH			81.0, C			27.3, CH_3_	1.24 s	2, 3, 6, 17	111.3, CH_2_	4.85 br s; 4.95 br s	14, 17
17	61.0, CH_2_	4.14 s	13, 15, 16	66.4, CH_2_	3.65 d (11.0); 3.72 d (11.0)	13, 15	27.0, CH_3_	1.27 s	2, 3, 6, 16	17.3, CH_3_	1.75 br s	14, 15, 16
18	65.9, CH_2_	3.43 d (10.0); 3.62 d (10.0)	4, 5, 19	29.0, CH_3_	1.21 s	3, 4, 5, 19	11.0, CH_3_	1.81 s	4, 5, 6	59.5, CH_2_	4.12 br s	8, 10, 11
19	64.3, CH_2_	3.48 d (10.5); 3.60 d (10.5)	4, 5, 18	179.4, C			11.3, CH_3_	1.74 br s	8, 9, 10	59.5, CH_2_	4.12 br s	6, 7, 8
20	19.7, CH_3_	1.09 s	15, 10	16.0, CH_3_	0.92 s	1, 5, 9, 10	60.0, CH_3_	4.14 br s	12, 14	27.6, CH_3_	1.28 s	2, 3, 4
MeO-				51.0, CH_3_	3.66 s	19						

a Spectra were recorded in methanol-*d*_*4*_, at 600 MHz (^1^H) and 150 MHz (^13^C); chemical shifts are given in ppm; *J* values are in parentheses and reported in Hz; assignments were confirmed by DQF-COSY, 1D-TOCSY, and HSQC experiments.

b Overlapped signal.

c HMBC correlations are from proton(s) stated to the indicated carbon.

Compound **14** had a molecular formula of C_21_H_34_O_5_ according to its HRESIMS (*m*/*z* 367.2477 [M + H]^+^). The spectroscopic features (Table 5) of this compound were in accordance with an *ent*-kaurane derivative.^22^ Compound **14** has been previously reported as an intermediate in the structural characterization of 15α-hydroxykaur-16(17)-ene-19-methyl ester,^24^ but in this study, it was isolated from a natural source for the first time. The NMR data of *ent*-15β,16α,17-trihydroxykaurane-19-methyl ester (**14**) are assigned for the first time here, as only the UV, IR, and MS data were present in the paper of Ali et al.^24^

The molecular formula of C_20_H_32_O_4_ was determined for compound **15** from its HRESIMS data (*m*/*z* 359.2188 [M + Na]^+^), indicating five indices of hydrogen deficiency. The ^13^C NMR spectrum (Table 5) exhibited 20 carbon resonances corresponding to four methyls, five methylenes, two hydroxymethylenes, two methines (sp^2^ carbons), one hydroxymethine, five quaternary carbons (four of them were sp^2^ carbons), and, finally, one carbonylic carbon. The ^1^H–^1^H COSY and 1D TOCSY experiments allowed us to determine the spin systems H_2_-2/H_2_-3, H_2_-7/H-8, and H-10/H_2_-15. The HMBC experiment (Table 5) allowed the assignment of structural fragments through the cross-peaks of H_2_-2/C-1, C-4, C-6, and C-16; H_2_-7/C-1, C-4, C-6, and C-9; H_3_-16 and H_3_-17/C-2 and C-6; H_3_-18/C-4 and C-6; H-8/C-10; H-14/C-12; and H_2_-20/C-12 and C-14. The hydroxymethylenes at C-15 and C-20 were attributed to the C-15 and C-20 positions based on the HMBC correlations of H-14/C-15, H_2_-15/C-13, H_2_-20/C-12, and H_2_-20/C-14. The hydroxymethine group at C-8 was defined through the HMBC correlation of H-8 with C-10 and C-19. The carbonyl group was attributed to the C-4 position based on the HMBC correlations of H_2_-2/C-4, H-7/C-4, and H_3_-18/C-4. Based on its spectroscopic data, **15** was identified as a retinoid derivative.^25^ The *E* configuration of the double bonds at C-9/C-10 and C-13/C-14 was inferred from the chemical shift of H-10 and H-14 and from the 1D ROESY spectra.^25^ Thus, **15** must be 4-oxo-8,20-dihydroxy-7,11-dihydroretinol.

Compound **16** had a molecular formula of C_20_H_34_O_4_, as deduced from its HRESIMS (*m*/*z* 339.2523 [M + H]^+^), indicating four degrees of hydrogen deficiency. The inspection of ^1^H and ^13^C NMR data revealed the presence of two methyls, six methylenes, two exomethylenes, three methines (sp^2^ carbons), two hydroxymethylenes, one secondary hydroxy group, one tertiary hydroxy group, and three sp^2^ quaternary carbons. The inspection of the HSQC, ^1^H–^1^H COSY, and 1D TOCSY experiments (Table 5) enabled the identification of the spin systems H_2_-1/H-2, H_2_-4/H-6, H_2_-8/H-10, and H_2_-12/H-17. The HMBC correlations of H-2/C-3, H_2_-5/C-7, H-10/C-18, H_2_-16/C-14, H_2_-18/C-11, and H_3_-20/C-3 led to defining compound **16** as an acyclic diterpenoid. The olefinic methylenes, i.e., δ_H_ 5.06 (1H, dd, *J* = 11.0, 1.6 Hz) and 5.24 dd (1H, dd, *J* = 17.0, 1.6 Hz) and δ_C_ 112.0 and δ_H_ 4.85 (1H, br s) and 4.95 (1H, br s) and δ_C_ 111.3, were located at C-1 and C-16, respectively, considering the HMBC correlations of H_2_-1 with C-3, H_2_-16 with C-14, and H_3_-17 with C-16. The hydroxymethylenes, i.e., δ_H_ 4.12 (2H, br s) and δ_C_ 59.5 and δ_H_ 4.12 (2H, br s) and δ_C_ 59.5, were assigned to C-7 and C-11, respectively, considering the HMBC correlations of H-6 with C-19, H-10 with C-18, H_2_-18 with C-10 and C-11, and H_2_-19 with C-6 and C-8. The secondary hydroxy group (δ_C_ 76.0; δ_H_ 4.03) was located at C-14, which was confirmed by the HMBC correlations of H_2_-16/C-14 and H_3_-17/C-14. The tertiary hydroxy group (δ_C_ 72.0) was located at C-3 according to the HMBC cross-peaks of H_2_-1/C-3, H-2/C-3, and H_3_-20/C-3. The *E* configuration of the double bonds at C-6/C-7 and C-10/C-11 was inferred from the chemical shift of H-10 and H-6 and the 1D ROESY spectra. CH_2_-19 and CH_2_-18 showed correlations peaks with H_2_-5 and H_2_-9, respectively; these correlations suggested that these double bonds are in the *E* configuration.^25^ Due to the extremely small amount of this compound, stereochemical investigations could not be performed. Thus, compound **16** was defined as 1,15-dehydro-2,14-dihydro-3,14,18,19-tetrahydroxygeranylgeraniol.

The known compounds were identified through comparisons with literature data of 5,4′-dihydroxy-6,7,8-trimethoxyflavone (xanthomicrol) (**17**),^26^ 5-hydroxy-7,4′-dimethoxyflavone (**18**),^27^ 5,4′-dihydroxy-6,7,8,3′,5′-pentamethoxyflavone (**19**),^28^ 5-hydroxy-6,7,8,4′-tetramethoxyflavone (5-demethyltangeretin) (**20**),^29^*ent*-kaurane-16,17-diol (**21**),^30^*ent-*kaur-16(17)-ene-6,19-dihydroxy-2-one (propsiadin) (**22**),^31^*ent*-trachyloban-6β,19-dihydroxy-2-one (**23**),^1^ 2-oxo-trachyloban-18,19-diol (**24**),^20^*ent*-kaur-16(17)-ene-18,19-dihydroxy-2-one (psiadin) (**25**),^23^ and *ent*-(16α)-16,17,19-trihydroxykauran-2-one (**26**).^32^

Diterpenes are among the most frequently occurring compounds in Asteraceae and can be considered of chemotaxonomic importance at the subfamilial level.^33^*Psiadia* is included in the Asterinae tribe and is mainly characterized by the labdane,^34^ kaurane,^22^ and trachylobane^35^ skeletal types.^33,36^ Previous research on the exudate of the aerial parts of *P. punctulata* has reported the presence of *ent*-kaurane and *ent*-trachylobane diterpenes.^1,5,20^ In the present work, other labdane-related diterpenoids showing a bridged ring system for rings C and D, namely, *ent*-atisane and *ent*-beyerene,^16^ were characterized by means of a combined experimental and computational approach.^10,37^ The *ent*-atisane diterpenoids are characterized by a tetracyclic skeleton presenting a perhydrophenanthrene moiety (rings A, B, and C), fused to a cyclohexane unit (ring D), bearing methyl groups at C-4, C-10, and C-16, and an extensive oxidative pattern.^9^ Furthermore, *ent*-beyerenes show a tetracyclic scaffold isomer of *ent*-kaurene structure, which is produced by a skeletal rearrangement.^16^ Both scaffolds present a bicyclo[3.2.1]octane moiety and a bridged ring system (C- and D-rings) with a spiro center at C-8. The C-17 is linked to C-16 in *ent*-kaurenes and to C-13 in *ent*-beyerenes.^38^ To date, for the family Asteraceae, *ent*-atisane diterpenoids have only been reported for five genera, namely, *Artemisia*, *Cassinia*, *Helianthus*, *Helicrysum*, and *Stevia*,^9^ while *ent*-beyerene has been isolated from the Astereae (*Baccharis*, *Brachycome*, and *Nidorella*), Gnaphalieae (*Myriocephalus*, *Calocephalus*, *Craspedia*, *Helipterum* syn. *Syncarpha*, and *Helichrysum*), and Calenduleae (*Dimorphotheca*) tribes.^33^ To the best of our knowledge, this is the first report of the presence of *ent*-atisane and *ent*-beyerene diterpenoids in *Psiadia* spp. and, particularly, of the co-occurrence of these skeletal types.

As part of a project aimed at isolating and characterizing metabolites with antimicrobial activity from plants belonging to the Lamiaceae and Asteraceae families,^1,39,40^ the bacteria that are etiological agents of the pathologies of the oral cavity were recently studied. Dental caries and periodontal diseases are common bacterial infections in humans. Dental biofilm, related to dental caries, is a dynamic, constantly metabolically active structure, and it represents a localized, progressive, and destructive process. Periodontitis is a gum disease and a severe chronic inflammation that causes the destruction of gum tissue.^41^ The increasing evidence of the central role played by the oral microbiome in many diseases, such as cardiovascular disease, pneumonia, rheumatoid arthritis, and cancer,^42^ makes the search of new antimicrobial compounds of interest. *S. mutans*, *S. aureus*, *L. plantarum*, and *T. denticola* are considered the most relevant bacteria in the transition of nonpathogenic commensal oral microbiota to biofilm, which contributes to the dental caries process and periodontal diseases. Based on this and the antimicrobial activity reported for *Psiadia* spp. preparations and compounds, the antibacterial potential of the extracts, fractions, and isolates of *P. punctulata* leaves was investigated against *S. mutans*, *S. aureus*, *L. plantarum*, and *T. denticola* through MIC, biofilm inhibition, and efflux pump inhibition.

The MIC values are shown in Table 6. The chloroform extract of the leaves of *P. punctulata* showed moderate or low antimicrobial activity. Among the nine fractions (A–I) analyzed, fraction F was the most active against *S. mutans*. All isolates were tested. Xanthomicrol (**17**), propsiadin (**22**), and psiadin (**25**) showed very low antimicrobial activity (Table 6), while the other compounds were inactive (data not shown). The possible ability of psiadin, an oxidized kaurene diterpene, to enhance the efficacy of the widely used oral disinfectant chlorhexidine was then studied. Due to the toxicity and resistance of common antiseptic agents,^43^ the formulation of antibacterial agents with plant bioactive molecules proved to be a promising strategy against multiresistant microbial strains.^44^ However, to the best of our knowledge, there are no reports in the literature investigating the antimicrobial effect of this diterpene in combination with oral antiseptics. Chlorhexidine is a biguanide largely used as an antiseptic agent to reduce the buildup of plaque. Thus, the antimicrobial activity of psiadin (**25**) alone and in combination with chlorhexidine was tested at different concentrations using a checkerboard dilution assay against the selected bacterial strains to verify a potential synergic or additive action (Table 7). A decrease in the MIC values of chlorhexidine and psiadin was observed when they were used in combination against *S. aureus* and *S. mutans*. These data were used to calculate the fractional inhibitory concentration (FIC) index. FICs of 0.9 and 0.7 were measured when incubating *S. aureus* with a combination of chlorhexidine and psiadin (50 and 25 μg/mL, respectively), while a FIC index of 0.85 was found for *S. mutans* (Table 7). These data could support the presence of an additive effect even if no proper synergic mechanism was identified (FIC ≤ 0.5).

**Table 6:** MIC Values for Compounds **17**, **22**, and **25**^a^

bacterial strains	chloroform extract	chloroform/methanol extract	**17**	**22**	**25**
*S. aureus*	1500	1800	>250	>250	125
*S. mutans*	500	700	180	250	140
*T. denticola*	1000	1000	200	125	100

a MIC values, expressed in μg/mL; data not shown where the MIC values were >2000, >500, and >500, respectively.

**Table 7:** MIC Values for Chlorhexidine and Chlorhexidine/Psiadin^a^

bacterial strains	CHX	CHX/psiadin, 25 μg/mL	CHX/psiadin, 50 μg/mL	CHX/psiadin, 100 μg/mL	CHX/psiadin, 200 μg/mL
*S. aureus*	2.5	1.25	1.25	0.6	0.3
*S. mutans*	1.25	1.25	0.6	0.6	0.3
*L. plantarum*	10	10	10	10	2.5
*T. denticola*	2.2	2.5	2.5	2.5	1.25

a MIC values, expressed in μg/mL, for chlorhexidine (CHX) alone and in combination with psiadin at different concentrations.

**Figure 1:**
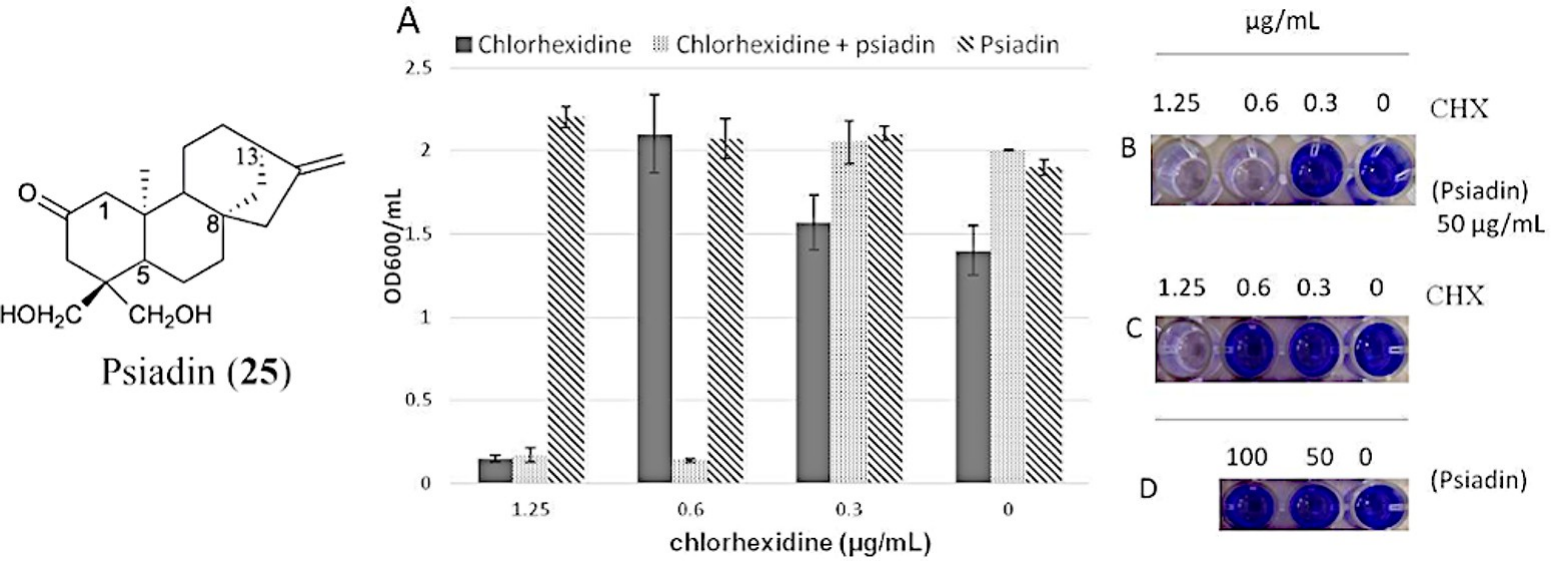
Biofilm of *S. mutans* in the presence of different concentrations of chlorhexidine (CHX), psiadin, and chlorhexidine–psiadin. Photographs of the biofilm of *S. mutans* attached to the surface of microtiter wells after washing and crystal violet (CV) staining in the presence of chlorhexidine–psiadin (B), chlorhexidine (C), and psiadin (D). The graphics were obtained by destaining the wells with 95% ethanol and measuring the absorbance of the CV at 595 nm (A).

The effect of psiadin, chlorhexidine, and their combination on the biofilm formation of *S. mutans* was then evaluated. The results indicated that psiadin did not affect biofilm formation (Figure 1, panel D), whereas, when used in combination (50 μg/mL, MIC 1/2) with chlorhexidine, it was able to reduce the concentration of chlorhexidine to fully inhibit the *S. mutans* biofilm (Figure 1, panel B). These results suggested that the use of psiadin in combination with chlorhexidine could enable a reduction in the dosage of the antiseptic agent required to achieve efficacy, thus minimizing the possible undesired effects of chlorhexidine-based treatments, such as mouth lining irritation, tooth discoloration, and tartar buildup.

**Figure 2:**
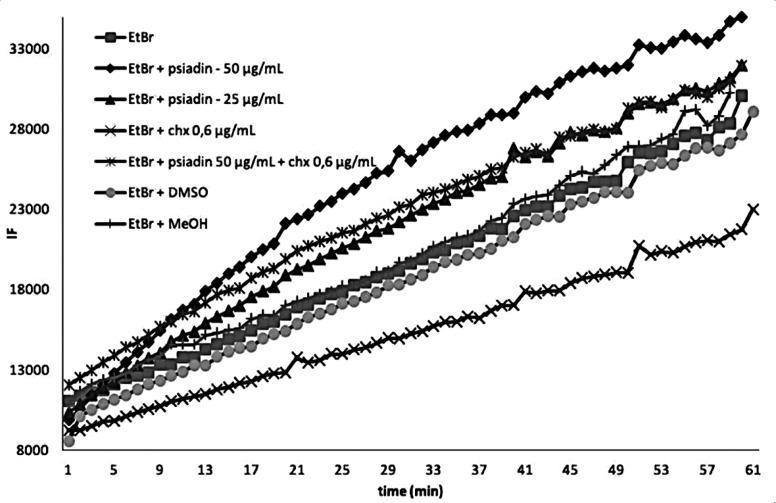
Ethidium bromide (EtBr) accumulation assay. Psiadin was added to the medium at two subinhibitory concentrations (50 and 25 μg/mL); psiadin (50 μg/mL) was also incubated in combination with subinhibitory concentrations of chlorhexidine (0.6 mL). Negative controls were performed using DMSO and 0.5% MeOH.

Among the numerous bacterial self-defense strategies, efflux pumps are one of the key tools for facilitating bacterial survival and contributing to multidrug resistance.^45^ The enhancement of the antimicrobial activity of several antiseptic agents when combined with natural compounds was frequently reported as a consequence of their inhibition of an efflux pump.^45^ In this light, the efflux pump inhibitory activity of psiadin via an ethidium bromide-based fluorometric assay was investigated for *S. mutans*. For both 50 and 25 μm/mL of psiadin, there was an increase in EtBr accumulation in a dose-dependent mode (Figure 2). The increase in EtBr accumulation fluorescence suggests that psiadin could act as an efflux pump inhibitor. Interestingly, chlorhexidine seemed to exert an opposite effect (Figure 2). When chlorhexidine is assayed alone, a significant decrease in EtBr accumulation is observed as compared to the control. This could be possibly due to the membrane-perturbing antimicrobial action of chlorhexidine. Indeed, the combination of chlorhexidine and psiadin showed a minor increase in EtBr accumulation compared with that observed with psiadin alone (Figure 2). This evidence suggested that the additive effect observed in the MIC values of the chlorhexidine–psiadin combination could be due to different mechanisms of action. These results could lead to the consideration that both chlorhexidine and psiadin have a general role in membrane perturbations through different mechanism, resulting in overall alterations of the cellular efflux system.

## Experimental Section

### General Experimental Procedures

Optical rotations were measured on a Atago AP-300 digital polarimeter with a 1 dm microcell and a sodium lamp (589 nm). NMR data were acquired in methanol-*d*_4_ (99.95%, Sigma-Aldrich, Milano, Italy) on a Bruker DRX-600 NMR spectrometer (Bruker BioSpin GmBH, Rheinstetten, Germany) equipped with a Bruker 5 mm TCI CryoProbe at 300 K. Data processing was carried out with Topspin 3.2 software. All 2D NMR spectra were acquired in methanol-*d*_4_ (99.95%, Sigma-Aldrich, Milano, Italy), and standard pulse sequences and phase cycling were used for DQF-COSY, HSQC, HMBC, and ROESY spectra. The NMR data were processed using Topspin 3.2 software (Bruker BioSpin GmBH, Rheinstetten, Germany). HRESIMS data were obtained in the positive ion mode on a Q Exactive Plus mass spectrometer, Orbitrap-based FT-MS system, equipped by an ESI source (Thermo Fisher Scientific Inc., Bremem, Germany). Column chromatography was performed over silica gel (70–220 mesh, Merck). RP HPLC separations were carried out using a Shimadzu LC-8A series pumping system equipped with a Shimadzu RID-10A refractive index detector and Shimadzu injector (Shimadzu Corporation, Kyoto, Japan) on a Waters XTerra Semiprep MS C18 column (300 mm × 7.8 mm i.d.) and a mobile phase consisting of a MeOH–H_2_O mixture at a flow rate of 2.0 mL/min. TLC separations were conducted using silica gel 60 F_254_ (0.20 mm thickness) plates (Merck, Darmstadt, Germany) and Ce(SO_4_)_2_/H_2_SO_4_ as spray reagent (Sigma-Aldrich, Milano, Italy).

### Plant Material

Leaves of *P*. *punctulata* were collected in Wadi Ghazal, Saudi Arabia, in June 2018 near Wadi Thee Ghazal about 25 km south of Taif City, Saudi Arabia (coordinates: 21° 08′ N, 40° 22′ E). The plant material was identified by A. Bader. A voucher specimen (SA/IT 2015/1a) was deposited in the Laboratory of Pharmacognosy at Umm Al-Qura University, Saudi Arabia.

### Extraction and Isolation

Dried leaves of *P. punctulata* (400 g) were extracted with solvents of increasing polarity, including *n*-hexane, CHCl_3_, and MeOH by exhaustive maceration (2 L) to give 6.0, 15.0, and 21.9 g of the respective dried residues. Part of the CHCl_3_ extract (7 g) was subjected to column chromatography (5 × 180 cm, collection volume 30 mL) over silica gel, eluting with CHCl_3_, followed by increasing concentrations of MeOH in CHCl_3_ (between 1% and 100%), gathering nine fractions (A–I). Fraction D (163.3 mg) was purified by RP HPLC with MeOH–H_2_O (7:3) as an eluent to give compounds **18** (2.0 mg, *t*_R_ 16 min), **19** (2.8 mg, *t*_R_ 21 min), **17** (3.0 mg, *t*_R_ 23 min), **20** (1.9 mg, *t*_R_ 28 min), and **3** (1.5 mg, *t*_R_ 35 min). Fractions E (192.7 mg), F (580.0 mg), and G (417.3 mg) were submitted to RP-HPLC with MeOH–H_2_O (3:2) as eluent to yield compounds **1** (2.0 mg, *t*_R_ 14 min) and **2** (3.0 mg, *t*_R_ 27 min) from fraction E; compounds **21** (1.5 mg, *t*_R_ 10 min), **14** (1.3 mg, *t*_R_ 15 min), **22** (4.0 mg, *t*_R_ 19 min), **23** (3.8 mg, *t*_R_ 22 min), **25** (6.2 mg, *t*_R_ 23 min), **4** (2.6 mg, *t*_R_ 27 min), and **24** (2.2 mg, *t*_R_ 29 min) from fraction F; and **14** (1.3 mg, *t*_R_ 15 min), **22** (1.0 mg, *t*_R_ 19 min), **9** (5.0 mg, *t*_R_ 23 min), and **24** (2.0 mg, *t*_R_ 29 min) from fraction G. Fraction H (900 mg) was separated by RP-HPLC eluting with MeOH–H_2_O (58:42) to give **12** (2.0 mg, *t*_R_ 4 min), **13** (1.3 mg, *t*_R_ 5 min), **15** (0.8 mg, *t*_R_ 11 min), **7** (2.3 mg, *t*_R_ 14 min), **16** (3.0 mg, *t*_R_ 20 min), **11** (5.0 mg, *t*_R_ 23 min), **8** (2.0 mg, *t*_R_ 30 min), **26** (3.5 mg, *t*_R_ 40 min), **5** (2.5 mg, *t*_R_ 48 min), **6** (0.5 mg, *t*_R_ 57 min), and **10** (2.0 mg, *t*_R_ 62 min).

#### *ent*-Atisan-6β,16α,17,19-tetraol (**1**):

white amorphous powder; [α]_D_ −4.0 (*c* 0.06, MeOH); ^1^H and ^13^C NMR see Table 1; HRESIMS *m*/*z* 361.2341 [M + Na]^+^ (calcd for C_20_H_34_O_4_Na, 361.2349), 303.23 [M + H – 18 – 18]^+^, 285.22 [M + H – 18 – 18 – 18]^+^.

#### *ent*-Atis-16(17)-en-2α,6β,18,19-tetraol (**2**):

white amorphous powder; [α]_D_ −15.0 (*c* 0.1, MeOH); ^1^H and ^13^C NMR, see Table 1; HRESIMS *m*/*z* 337.2364 [M + H]^+^ (calcd for C_20_H_33_O_4_, 337.2373), 319.23 [M + H – 18]^+^, 301.21 [M + H – 18 – 18]^+^, 283.20 [M + H – 18 – 18 – 18]^+^.

#### *ent*-Atisan-2α,16α,18-triol (**3**):

white amorphous powder; [α]_D_ −23 (*c* 0.1, MeOH); ^1^H and ^13^C NMR, see Table 1; HRESIMS *m*/*z* 345.2396 [M + Na]^+^ (calcd for C_20_H_34_O_3_Na 345.2400), 305.24 [M + H – 18]^+^, 287.23 [M + H – 18 – 18]^+^, 269.22 [M + H – 18 – 18 – 18]^+^.

#### *ent*-18,19-Dihydroxybeyer-15-en-2-one (**4**):

white amorphous powder; [α]_D_ −9.6 (*c* 0.1, MeOH); ^1^H and ^13^C NMR, see Table 2; HRESIMS *m*/*z* 319.2262 [M + H]^+^ (calcd for C_20_H_31_O_3_, 319.2267), 301.21 [M + H – 18]^+^, 283.20 [M + H – 18 – 18]^+^.

#### *ent*-Beyer-15-en-6β,17,19-triol (**5**):

white amorphous powder; [α]_D_ −20 (*c* 0.1, MeOH); ^1^H and ^13^C NMR, see Table 2; HRESIMS *m*/*z* 343.2238 [M + Na]^+^ (calcd for C_20_H_33_O_3_Na 343.2244), 303.23 [M + H – 18]^+^, 285.22 [M + H – 18 – 18]^+^, 267.21 [M + H – 18 – 18 – 18]^+^.

#### *ent*-Beyer-15-en-2α,18,19-triol (**6**):

white amorphous powder; [α]_D_ +16.6 (*c* 0.1, MeOH); ^1^H and ^13^C NMR, see Table 2; HRESIMS *m*/*z* 321.2417 [M + H]^+^ (calcd for C_20_H_33_O_3_, 321.2424), 303.23 [M + H – 18]^+^, 285.22 [M + H – 18 – 18]^+^, 267.21 [M + H – 18 – 18 – 18]^+^, 343.2239 [M + Na]^+^.

#### *ent*-Beyer-15-en-2α,17,18-triol (**7**):

white amorphous powder; [α]_D_ −32.5 (*c* 0.1, MeOH); ^1^H and ^13^C NMR, see Table 3; HRESIMS *m*/*z* 321.2422 [M + H]^+^ (calcd for C_20_H_33_O_3_ 321.2424), 303.23 [M + H – 18]^+^, 285.22 [M + H – 18 – 18]^+^.

#### *ent*-17,19-Dihydroxybeyer-15-en-17-arabinopyranoside (**8**):

[α]_D_ +20.4 (*c* 0.1, MeOH); ^1^H and ^13^C NMR, see Table 3; HRESIMS *m*/*z* 459.2705 [M + Na]^+^ (calcd for C_25_H_40_O_6_Na 459.2717), 437.2888 [M + H]^+^, 305.2469 [M + H – 132]^+^.

#### *ent*-6β,17-Dihydroxybeyer-15-en-2-one (**9**):

white amorphous powder; [α]_D_ −17.0 (*c* 0.1, MeOH); ^1^H and ^13^C NMR, see Table 3; HRESIMS *m*/*z* 319.2260 [M + H]^+^ (calcd for C_20_H_31_O_3_ 319.2268), 301.21 [M + H – 18]^+^, 283.20 [M + H – 18 – 18]^+^.

#### *ent*-Trachyloban-2α,18,19-triol (**10**):

white amorphous powder; [α]_D_ −14.0 (*c* 0.1, MeOH); ^1^H and ^13^C NMR, see Table 4; HRESIMS *m*/*z* 321.2409 [M + H]^+^ (calcd for C_20_H_33_O_3_ 321.2424), 303.2311 [M + H – 18]^+^, 285.2204 [M + H – 18 – 18]^+^, 267.21 [M + H – 18 – 18 – 18]^+^.

#### *ent*-Trachyloban-17,18,19-triol (**11**):

white amorphous powder; [α]_D_ −22.0 (*c* 0.1, MeOH); ^1^H and ^13^C NMR, see Table 4; HRESIMS *m*/*z* 321.2417 [M + H]^+^ (calcd for C_20_H_33_O_3_ 321.2424), 303.23 [M + H – 18]^+^, 285.22 [M + H – 18 – 18]^+^, 267.21 [M + H – 18 – 18 – 18]^+^.

#### *ent*-18,19-Dihydroxytrachyloban-17-oic acid (**12**):

white amorphous powder; [α]_D_ −61.0 (*c* 0.1, MeOH); ^1^H and ^13^C NMR, see Table 4; HRESIMS *m*/*z* 335.2206 [M + H]^+^ (calcd for C_20_H_31_O_4_ 335.2217), 317.22 [M + H – 18]^+^, 299.19 [M + H – 18 – 18]^+^.

#### *ent*-17,18,19-Trihydroxykaur-15-en-2-one (**13**):

white amorphous powder; [α]_D_ −50.0 (*c* 0.1, MeOH); ^1^H and ^13^C NMR, see Table 5; HRESIMS *m*/*z* 335.2212 [M + H]^+^ (calcd for C_20_H_31_O_4_ 335.2222), 317.21 [M + H – 18]^+^, 299.20 [M + H – 18 – 18]^+^.

#### *ent*-15β,16α,17-Trihydroxykauran-19-methyl ester (**14**):

white amorphous powder; [α]_D_ −58.0 (*c* 0.1, MeOH); ^1^H and ^13^C NMR, see Table 5; HRESIMS *m*/*z* 367.2477 [M + H]^+^ (calcd for C_21_H_35_O_5_ 367.2479), 349.23 [M + H – 18]^+^, 331.22 [M + H – 18 – 18]^+^, 313.21 [M + H – 18 – 18 – 18]^+^.

#### 4-Oxo-8,20-dihydroxy-7,11-dihydroretinol (**15**):

white amorphous powder; [α]_D_ −3.7 (*c* 0.1, MeOH); ^1^H and ^13^C NMR, see Table 5; HRESIMS *m*/*z* 359.2188 [M + Na]^+^ (calcd for C_20_H_32_O_4_Na 359.2193).

#### 1,15-Dehydro-2,14-dihydro-3,14,18,19-tetrahydroxygeranylgeraniol (**16**):

white amorphous powder; [α]_D_ −7.0 (*c* 0.1, MeOH); ^1^H and ^13^C NMR, see Table 5; HRESIMS *m*/*z* 339.2523 [M + H]^+^ (calcd for C_20_H_35_O_4_ 339.2530), 321.24 [M + H – 18]^+^, 303.23 [M + H – 18 – 18]^+^, 285.22 [M + H – 18 – 18 – 18]^+^, 267.21 [M + H – 18 – 18 – 18 – 18]^+^.

### Structural Determination by the Quantum Mechanical Approach

The starting 3D chemical structures of all possible diastereomers under study, namely, compounds **1**, **4**, **6**, and **7**, were built with Maestro11.1^46^ (RRID:SCR_016748). Optimizations of the starting 3D structures were performed with MacroModel 11.5^46^ (RRID:SCR_016748) using the OPLS force field^46^ and the Polak-Ribier conjugate gradient algorithm (PRCG, maximum derivative less than 0.001 kcal/mol). For these compounds, exhaustive conformational searches at the empirical molecular mechanics (MM) level with the MCMM method (50 000 steps) and the LMCS method (50 000 steps) were performed to allow a full exploration of the conformational space. Also, molecular dynamics simulations were achieved at 450, 600, 700, and 750 K, with a time step of 2.0 fs, an equilibration time of 0.1 ns, and a simulation time of 10 ns. For each diastereomer, all the conformers obtained from the conformational searches were minimized (PRCG, maximum derivative less than 0.001 kcal/mol) and superimposed. Then, the “redundant conformer elimination” module of Macromodel 11.5^13,46^ was used to select nonredundant conformers, excluding those differing more than 21.0 kJ/mol (5.02 kcal/mol) from the most energetically favored conformation and setting a 0.5 Å RMSD (root-mean-square deviation) minimum cutoff for saving structures. The subsequent QM calculations were performed using Gaussian 09 software (RRID:SCR_014897). The most energetically favored conformers for each diastereomer of each compound identified at the MM level were geometry optimized at the MPW1PW91/6-31G(d) level of theory. After the optimization of the geometries, the conformers were visually inspected to remove further redundant conformers. The computation of the ^13^C and ^1^H NMR chemical shifts was performed on the selected conformers for the different diastereomers of compounds, using the MPW1PW91 functional and the 6-31G(d,p) basis set. Final ^13^C and ^1^H NMR chemical shift sets of data for each of the diastereomers were extracted and computed considering the influence of each conformer on the total Boltzmann distribution considering the relative energies. Calibrations of calculated ^13^C and ^1^H chemical shifts were performed following the multistandard approach (MSTD).^47^ sp^2 13^C and ^1^H NMR chemical shifts were computed using benzene as reference compound,^47^ while tetramethylsilane was used for computing sp^3 13^C and ^1^H chemical shift data.

Experimental and calculated ^13^C and ^1^H NMR chemical shifts were compared computing the Δδ parameter:
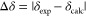
where δ_exp_ (ppm) and δ_calc_ (ppm) are the ^13^C/^1^H experimental and calculated chemical shifts, respectively.

The MAEs for all the considered diastereomers were computed using the following equation:
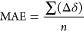
defined as the summation (∑) of the *n* computed absolute error values (Δδ), normalized to the number of chemical shifts considered (*n*). Furthermore, DP4+ probabilities^14^ related to all the stereoisomers for each compounds were computed considering both ^1^H and ^13^C NMR chemical shifts and comparing them with the related experimental data.

### Antimicrobial Experiments

*Staphylococcus aureus* ATCC 23235, *Streptococcus mutans* Clarke ATCC 25175, *Treponema denticola* ATCC 35405, and *Lactobacillus plantarum* (Orla-Jensen) Bergey et al. ATCC 8014 were purchased from ATCC (American Type Culture Collection). *E. coli* JM109 competent cells were obtained by Promega Italia S.r.l. MICs were determined. *S. aureus* and *S. mutans* were grown aerobically in brain heart infusion broth (BHI) rich medium at 37 °C. *T. denticola* was grown in BHI rich medium, while *L. plantarum* was amplified in Man, Rogosa & Sharpe broth (MRS broth), following the ATCC guidelines. *T. denticola* and *L. plantarum* were cultured in anaerobic conditions in a 3.5 L anaerobic jars (Oxoid) using AnaeroGen (ThermoFisher Scientific) for the generation of anaerobic conditions and incubated at 37 °C for 48 h. The analysis of antibacterial activity of the extract, fractions, and pure compounds, obtained following a bioguided chromatographic separation, was carried out in BHI for *S. aureus*, *S. mutans*, and *T. denticola* and in MRS for *L. plantarum*. The samples were dissolved in 100% dimethyl sulfoxide (DMSO) at different concentrations (extract: from 500 to 1500 μg/mL; fractions: from 20 to 200 μg/mL; pure compounds: from 10 to 200 μg/mL), added to each well and bacterial suspensions (0.5 × 10^5^ CFU/mL), and then incubated at 37 °C for 24 and 48 h for anaerobic bacteria *T. denticola* and *L. plantarum*. Cell absorbance was measured at 600 nm using a Tecan Infinite 200 Pro spectrophotometer. A blank control (sterile culture medium, without compounds and suspensions of microorganisms) and a vehicle control (sterile culture medium with DMSO) were used. The MIC was determined as the lowest drug concentration that inhibited visible bacterial growth. All determinations were done in triplicate.

### Determination of Interaction between Psiadin and Chlorhexidine

A checkerboard assay using psiadin at 0, 25, 50, 100, and 200 μg/mL combined with chlorhexidine at 5, 2.5, 1.25, 0.6, 0.3, and 0 μg/mL was performed. The MIC was determined as previously described. Then, the FIC indexes were determined using the following formula:
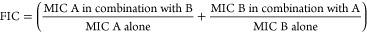


### Crystal Violet Assay

An overnight culture adjusting the OD_600_ nm to 0.1 (10^8^ CFU/mL) was added to 100 μL of fresh BHI liquid medium supplemented with 0.2% sucrose in each flat-bottom well with different concentrations of psiadin (0, 50, 100 μg/mL). These were tested alone and in combination with chlorhexidine (1.25, 0.6, 0.3, and 0 μg/mL respectively). The combination experiment was performed to evaluate the synergistic effects of psiadin and chlorhexidine on biofilm formation. Chlorhexidine (0.2%) was set as the positive control. The plates were then incubated at 37 °C for 24 h without agitation. After incubation, the growth medium was gently removed, washed three times with sterile phosphate-buffered saline (PBS), and replaced with 100 μL of crystal violet (CV). The plates were incubated for 10 min at room temperature. The excess CV solution was removed, the wells were rinsed three times with PBS, and the bound CV was dissolved by adding 100 μL of 95% ethanol. The absorbance of the solution was measured at a wavelength of 595 nm by a microplate reader. Each experiment was performed with triplicate samples at each time point.

### Efflux Pump Assay

An ethidium bromide (EtBr) accumulation assay for *S. mutans* was adapted following the procedure of Rodrigues et al.^48^ One colony of *S. mutans* was inoculated in 10 mL of BHI medium and grown overnight with shaking at 37 °C. Cells were washed twice in PBS buffer to remove the rich BHI medium and resuspended in PBS buffer at 0.6 OD_600_/mL. Cells were incubated in PBS supplemented with glucose at a final concentration of 0.4%. Psiadin (**25**) was tested at two subinhibitory concentrations (50 and 25 μg/mL) together with 0.4% glucose and EtBr at a final concentration of 2 μg/mL. Psiadin at 25 μg/mL was also incubated in combination with subinhibitory concentrations of chlorhexidine (0.6 μg/mL). Negative controls were performed using DMSO and MeOH, 0.5% each. Samples were prepared in separate wells of a 96-well plate. Ethidium bromide was added to each well to a final concentration of 2 μg/mL, allowing an accumulation into cells without causing significant inhibition of growth. The 96-well plate was placed in a Tecan Infinite 200 Pro spectrophotometer spectrofluorimeter, and fluorescence data were recorded every 60 s for 60 min at 37 °C using an excitation wavelength of 525 nm and an emission wavelength of 605 nm. Fluorescence intensity was monitored over time, measuring EtBr accumulation.

### Statistical Analysis

All determinations were done in triplicate, Student’s *t* test was used, and the data were considered statistically significant at *p* ≤ 0.05.

## Supporting Information Available

NMR and MS spectra of compounds **1**–**15**; experimental and calculated NMR data of compounds **1**, **4**, **6**, and **7** (PDF)
