# Supplementary material for: Diterpenoid Constituents of Psiadiapunctulata and Evaluation of Their Antimicrobial Activity
Source: J Nat Prod. 2022 Jun 24;85(7):1667–80. doi: 10.1021/acs.jnatprod.1c01093 (PMC9315948; doi:10.1021/acs.jnatprod.1c01093)
Supplement: Supplementary file 1 — np1c01093_si_001.pdf [file np1c01093_si_001.pdf]

**Supporting Information for:**

# Labdane-Related Diterpenoids from Psiadia punctulata: Structural Insights and Evaluation of Their Antimicrobial Activity

Giuliana Donadio,[†,#] Maria Giovanna Chini,[‡,#] Valentina Parisi,[†,§] Francesca Mensitieri,[⊥] Nicola Malafronte,[†] Giuseppe Bifulco,[†] Angela Bisio,[∥,*] Nunziatina De Tommasi,[†,*] Ammar Bader[∇]

[†]Department of Pharmacy, University of Salerno, Via Giovanni Paolo II 132, 84084, Fisciano, Salerno, Italy.

[‡]Department of Biosciences and Territory, University of Molise, Contrada Fonte Lappone, I-86090, Pesche, Isernia, Italy.

[§]PhD Program in Drug Discovery and Development, Department of Pharmacy, University of Salerno, Via Giovanni Paolo II 132, 84084, Fisciano, Salerno, Italy.

[⊥]Department of Medicine, Surgery and Dentistry "Scuola Medica Salernitana", University of Salerno, Via Salvador Allende, 84081, Baronissi, Italy

[∥]Department of Pharmacy, University of Genova, Viale Cembrano 4, 16148, Genova, Italy

[∇]Department of Pharmacognosy, Umm Al-Qura University, 21955 Makkah, Saudi Arabia.

[#]These authors have contributed equally to this work

**Figure S 1.** $^{1}$H NMR spectrum of compound **1** ($CD_3OD$, 600 MHz)

**Figure S 2.** COSY spectrum of compound **1** ($CD_3OD$, 600 MHz)

**Figure S 3.** HSQC spectrum of compound **1** ($CD_3OD$, 600 MHz)

**Figure S 4.** HMBC spectrum of compound **1** ($CD_3OD$, 600 MHz)

**Figure S 5.** $^{13}$C NMR spectrum of compound **1** ($CD_3OD$, 600 MHz)

**Figure S 6.** HRESIMS of compound **1**

**Figure S 7.** $^{1}$H NMR spectrum of compound **2** ($CD_3OD$, 600 MHz)

**Figure S 8.** COSY spectrum of compound **2** ($CD_3OD$, 600 MHz)

**Figure S 9.** HSQC spectrum of compound **2** ($CD_3OD$, 600 MHz)

**Figure S 10.** HMBC spectrum of compound **2** ($CD_3OD$, 600 MHz)

**Figure S 11.** $^{13}$C NMR spectrum of compound **2** ($CD_3OD$, 600 MHz)

**Figure S 12.** HRESIMS of compound **2**

**Figure S 13.** $^{1}$H NMR spectrum of compound **3** ($CD_3OD$, 600 MHz)

**Figure S 14.** COSY spectrum of compound **3** ($CD_3OD$, 600 MHz)

**Figure S 15.** HSQC spectrum of compound **3** ($CD_3OD$, 600 MHz)
**Figure S 16.** HMBC spectrum of compound **3** ($CD_3OD$, 600 MHz)
**Figure S 17.** $^{13}C$ NMR spectrum of compound **3** ($CD_3OD$, 600 MHz)
**Figure S 18.** HRESIMS of compound **3**
**Figure S 19.** $^{1}H$ NMR spectrum of compound **4** ($CD_3OD$, 600 MHz)
**Figure S 20.** COSY spectrum of compound **4** ($CD_3OD$, 600 MHz)
**Figure S 21.** HSQC spectrum of compound **4** ($CD_3OD$, 600 MHz)
**Figure S 22.** HMBC spectrum of compound **4** ($CD_3OD$, 600 MHz)
**Figure S 23.** $^{13}C$ NMR spectrum of compound **4** ($CD_3OD$, 600 MHz)
**Figure S 24.** HRESIMS of compound **4**
**Figure S 25.** $^{1}H$ NMR spectrum of compound **5** ($CD_3OD$, 600 MHz)
**Figure S 26.** HSQC spectrum of compound **5** ($CD_3OD$, 600 MHz)
**Figure S 27.** HMBC spectrum of compound **5** ($CD_3OD$, 600 MHz)
**Figure S 28.** $^{13}C$ NMR spectrum of compound **5** ($CD_3OD$, 600 MHz)
**Figure S 29.** HRESIMS of compound **5**
**Figure S 30.** $^{1}H$ NMR spectrum of compound **6** ($CD_3OD$, 600 MHz)
**Figure S 31.** COSY spectrum of compound **6** ($CD_3OD$, 600 MHz)
**Figure S 32.** HSQC spectrum of compound **6** ($CD_3OD$, 600 MHz)
**Figure S 33.** HMBC spectrum of compound **6** ($CD_3OD$, 600 MHz)
**Figure S 34.** HRESIMS of compound **6**
**Figure S 35.** $^{1}H$ NMR spectrum of compound **7** ($CD_3OD$, 600 MHz)
**Figure S 36.** COSY spectrum of compound **7** ($CD_3OD$, 600 MHz)
**Figure S 37.** HSQC spectrum of compound **7** ($CD_3OD$, 600 MHz)
**Figure S 38.** HMBC spectrum of compound **7** ($CD_3OD$, 600 MHz)
**Figure S 39.** $^{13}C$ NMR spectrum of compound **7** ($CD_3OD$, 600 MHz)
**Figure S 40.** HRESIMS of compound **7**
**Figure S 41.** $^{1}H$ NMR spectrum of compound **8** ($CD_3OD$, 600 MHz)
**Figure S 42.** COSY spectrum of compound **8** ($CD_3OD$, 600 MHz)
**Figure S 43.** HSQC spectrum of compound **8** ($CD_3OD$, 600 MHz)
**Figure S 44.** HMBC spectrum of compound **8** ($CD_3OD$, 600 MHz)
**Figure S 45.** $^{13}C$ NMR spectrum of compound **8** ($CD_3OD$, 600 MHz)
**Figure S 46.** HRESIMS of compound **8**
**Figure S 47.** $^{1}H$ NMR spectrum of compound **9** ($CD_3OD$, 600 MHz).
**Figure S 48.** COSY spectrum of compound **9** ($CD_3OD$, 600 MHz)

**Figure S 49.** HSQC spectrum of compound **9** ($CD_3OD$, 600 MHz)
**Figure S 50.** HMBC spectrum of compound **9** ($CD_3OD$, 600 MHz)
**Figure S 51.** $^{13}C$ NMR spectrum of compound **9** ($CD_3OD$, 600 MHz)
**Figure S 52.** HRESIMS of compound **9**
**Figure S 53.** $^{1}H$ NMR spectrum of compound **10** ($CD_3OD$, 600 MHz)
**Figure S 54.** COSY spectrum of compound **10** ($CD_3OD$, 600 MHz)
**Figure S 55.** HSQC spectrum of compound **10** ($CD_3OD$, 600 MHz)
**Figure S 56.** HMBC spectrum of compound **10** ($CD_3OD$, 600 MHz)
**Figure S 57.** $^{13}C$ NMR spectrum of compound **10** ($CD_3OD$, 600 MHz)
**Figure S 58.** HRESIMS of compound **10**
**Figure S 59.** $^{1}H$ NMR spectrum of compound **11** ($CD_3OD$, 600 MHz)
**Figure S 60.** COSY spectrum of compound **11** ($CD_3OD$, 600 MHz)
**Figure S 61.** HSQC spectrum of compound **11** ($CD_3OD$, 600 MHz)
**Figure S 62.** HMBC spectrum of compound **11** ($CD_3OD$, 600 MHz)
**Figure S 63.** $^{13}C$ NMR spectrum of compound **11** ($CD_3OD$, 600 MHz)
**Figure S 64.** HRESIMS of compound **11**
**Figure S 65.** $^{1}H$ NMR spectrum of compound **12** ($CD_3OD$, 600 MHz)
**Figure S 66.** COSY spectrum of compound **12** ($CD_3OD$, 600 MHz)
**Figure S 67.** HSQC spectrum of compound **12** ($CD_3OD$, 600 MHz)
**Figure S 68.** HMBC spectrum of compound **12** ($CD_3OD$, 600 MHz)
**Figure S 69.** $^{13}C$ NMR spectrum of compound **12** ($CD_3OD$, 600 MHz)
**Figure S 70.** HRESIMS of compound **12**
**Figure S 71.** $^{1}H$ NMR spectrum of compound **13** ($CD_3OD$, 600 MHz)
**Figure S 72.** COSY spectrum of compound **13** ($CD_3OD$, 600 MHz)
**Figure S 73.** HSQC spectrum of compound **13** ($CD_3OD$, 600 MHz)
**Figure S 74.** HMBC spectrum of compound **13** ($CD_3OD$, 600 MHz)
**Figure S 75.** $^{13}C$ NMR spectrum of compound **13** ($CD_3OD$, 600 MHz)
**Figure S 76.** HRESIMS of compound **13**
**Figure S 77.** $^{1}H$ NMR spectrum of compound **14** ($CD_3OD$, 600 MHz)
**Figure S 78.** COSY spectrum of compound **14** ($CD_3OD$, 600 MHz)
**Figure S 79.** HSQC spectrum of compound **14** ($CD_3OD$, 600 MHz)
**Figure S 80.** HMBC spectrum of compound **14** ($CD_3OD$, 600 MHz)
**Figure S 81.** $^{13}C$ NMR spectrum of compound **14** ($CD_3OD$, 600 MHz)
**Figure S 82.** HRESIMS of compound **14**

**Figure S 83.** $^1$H NMR spectrum of compound **15** ($CD_3OD$, 600 MHz)

**Figure S 84.** COSY spectrum of compound **15** ($CD_3OD$, 600 MHz)

**Figure S 85.** HSQC spectrum of compound **15** ($CD_3OD$, 600 MHz)

**Figure S 86.** HMBC spectrum of compound **15** ($CD_3OD$, 600 MHz)

**Figure S 87.** $^{13}$C NMR spectrum of compound **15** ($CD_3OD$, 600 MHz)

**Figure S 88.** HRESIMS of compound **15**

**Figure S 89.** $^1$H NMR spectrum of compound **16** ($CD_3OD$, 600 MHz)

**Figure S 90.** COSY spectrum of compound **16** ($CD_3OD$, 600 MHz)

**Figure S 91.** HSQC spectrum of compound **16** ($CD_3OD$, 600 MHz)

**Figure S 92.** HMBC spectrum of compound **16** ($CD_3OD$, 600 MHz)

**Figure S 93.** $^{13}$C NMR spectrum of compound **16** ($CD_3OD$, 600 MHz)

**Figure S 94.** HRESIMS of compound **16**

**Table S95**. $^{13}$C experimental and calculated NMR chemical shifts for **1a-d**, with [a]$|\Delta\delta|(^{13}C)$ and [b]MAE values.

**Table S96**. $^1$H experimental and calculated NMR chemical shifts for **1a-d**, with [a]$|\Delta\delta|(^{1}H)$ and [b]MAE values.

**Table S97**. $^{13}$C experimental and calculated NMR chemical shifts for **4a-b**, with [a]$|\Delta\delta|(^{13}C)$ and [b]MAE values.

**Table S98**. $^1$H experimental and calculated NMR chemical shifts for **4a-b**, with [a]$|\Delta\delta|(^{1}H)$ and [b]MAE values.

**Table S99**. $^{13}$C experimental and calculated NMR chemical shifts for **6a-b**, with [a]$|\Delta\delta|(^{13}C)$ and [b]MAE values.

**Table S100**. $^1$H experimental and calculated NMR chemical shifts for **6a-b**, with [a]$|\Delta\delta|(^{1}H)$ and [b]MAE values.

**Table S101**. $^{13}$C experimental and calculated NMR chemical shifts for **7a-b**, with [a]$|\Delta\delta|(^{13}C)$ and [b]MAE values.

**Table S102**. $^1$H experimental and calculated NMR chemical shifts for **7a-b**, with [a]$|\Delta\delta|(^{1}H)$ and [b]MAE values.

**Table S103.** $^{13}$C/$^1$H MAE (ppm) Values and DP4+ Data Reported for All the Possible Relative Stereoisomers for Compounds **1**, **4**, **6** and **7**.

**Figure S 1.** $^1H$ NMR spectrum of compound **1** ($CD_3OD$, 600 MHz)

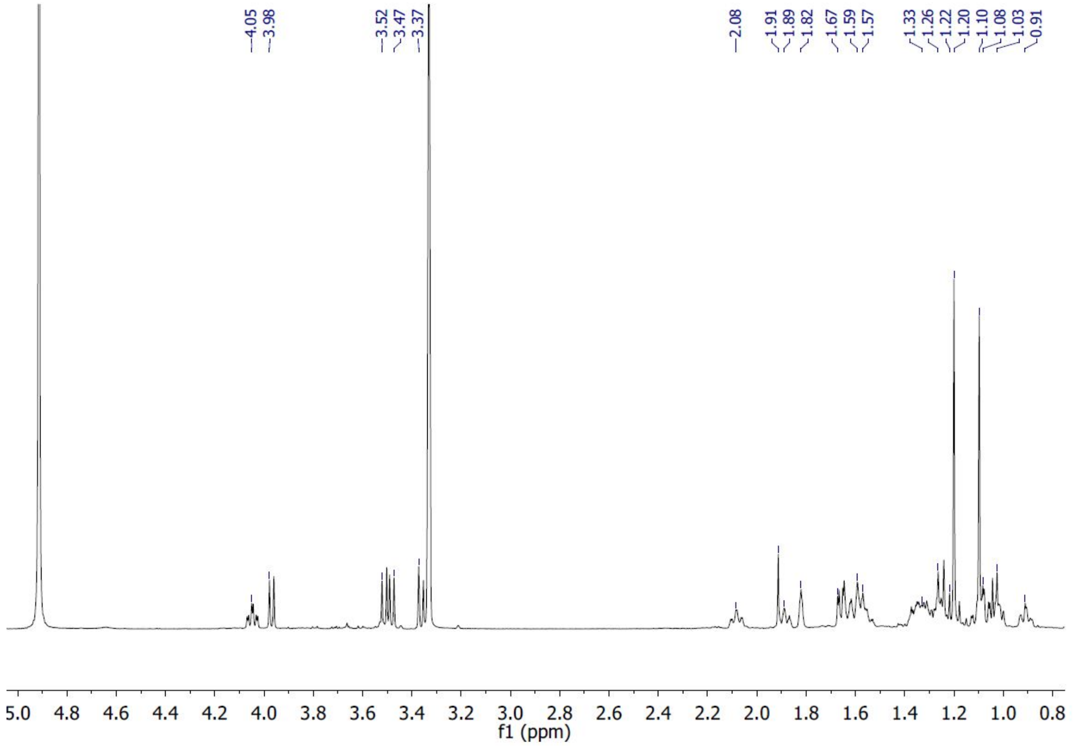


**Figure S 2.** COSY spectrum of compound **1** ($CD_3OD$, 600 MHz)

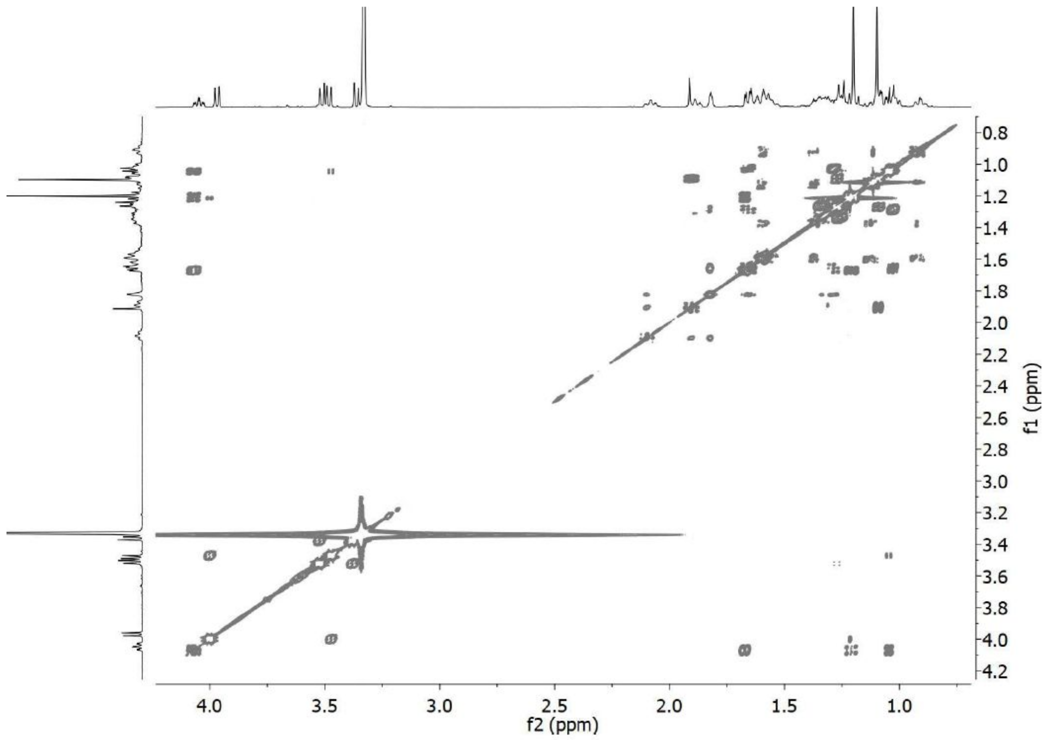

**Figure S 3.** HSQC spectrum of compound **1** ($CD_3OD$, 600 MHz)

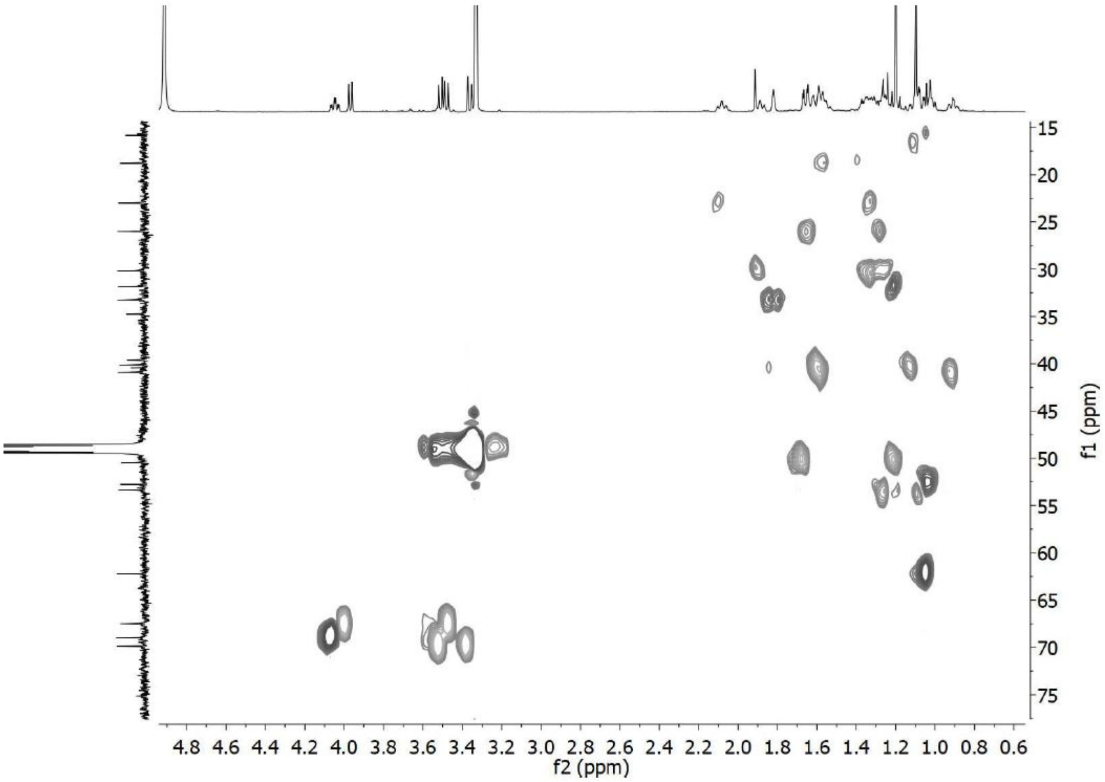


**Figure S 4.** HMBC spectrum of compound **1** ($CD_3OD$, 600 MHz)

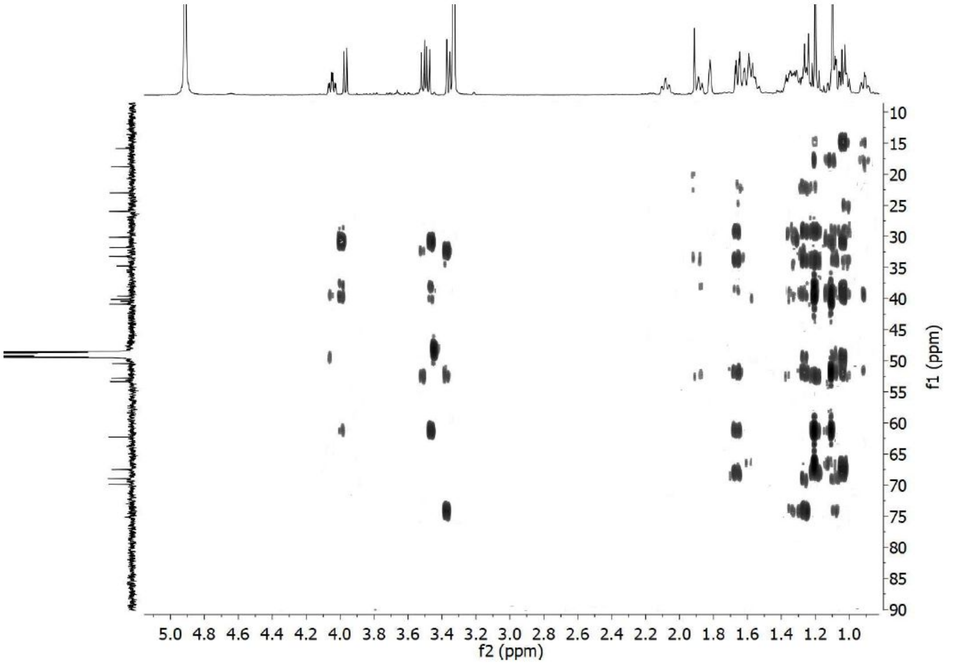

**Figure S 5.** $^{13}C$ NMR spectrum of compound **1** ($CD_3OD$, 600 MHz)

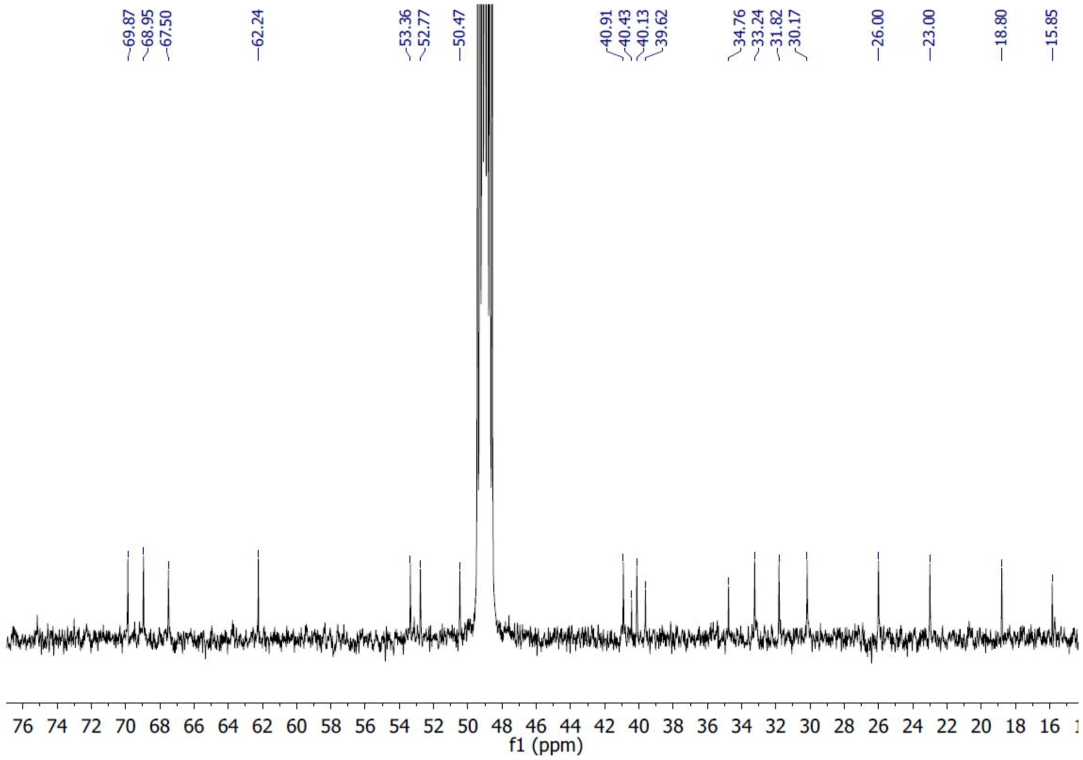


**Figure S 6.** HRESIMS of compound **1**

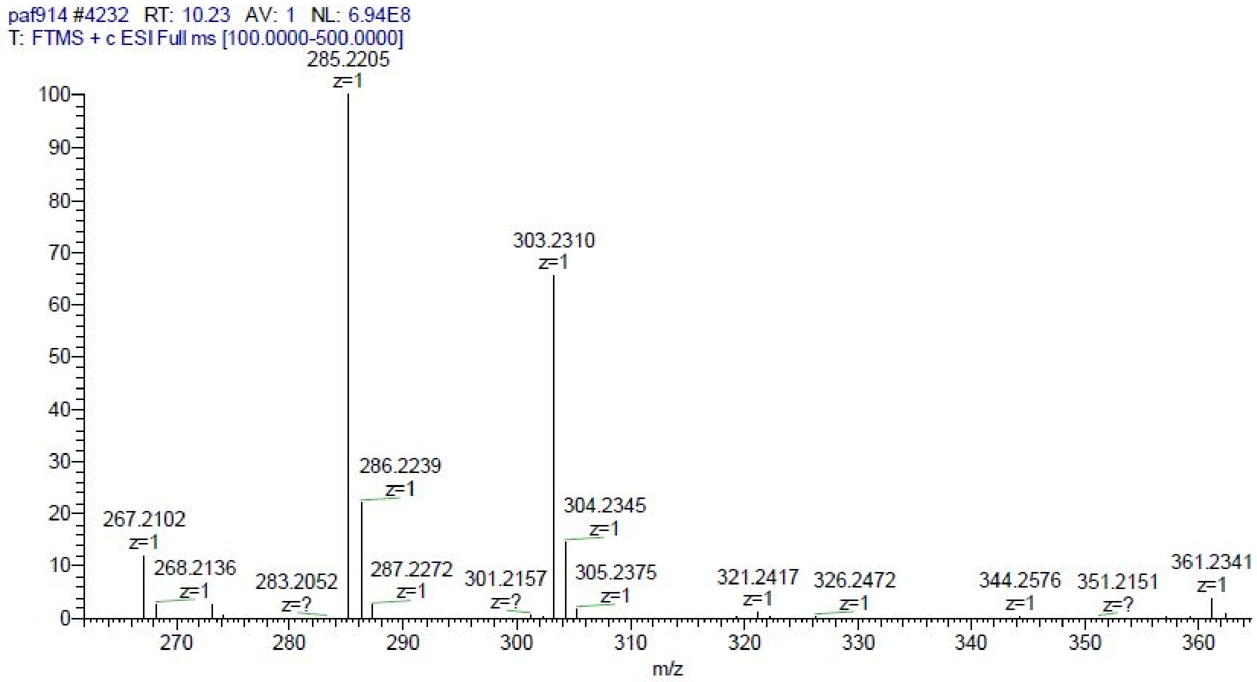

**Figure S 7.** $^{1}$H NMR spectrum of compound **2** ($CD_3OD$, 600 MHz)

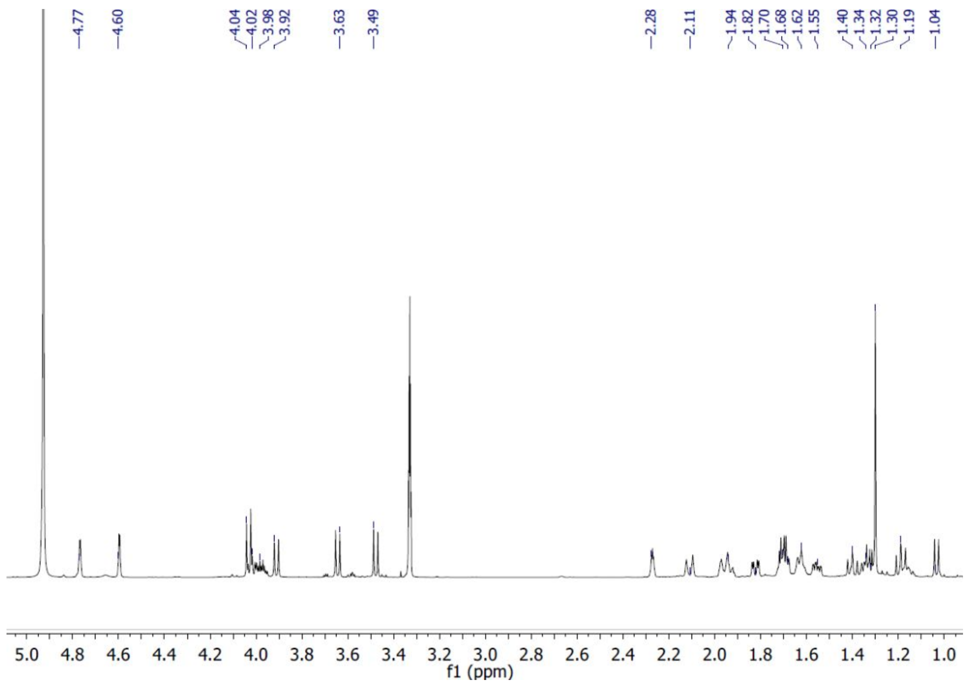


**Figure S 8.** COSY spectrum of compound **2** ($CD_3OD$, 600 MHz)

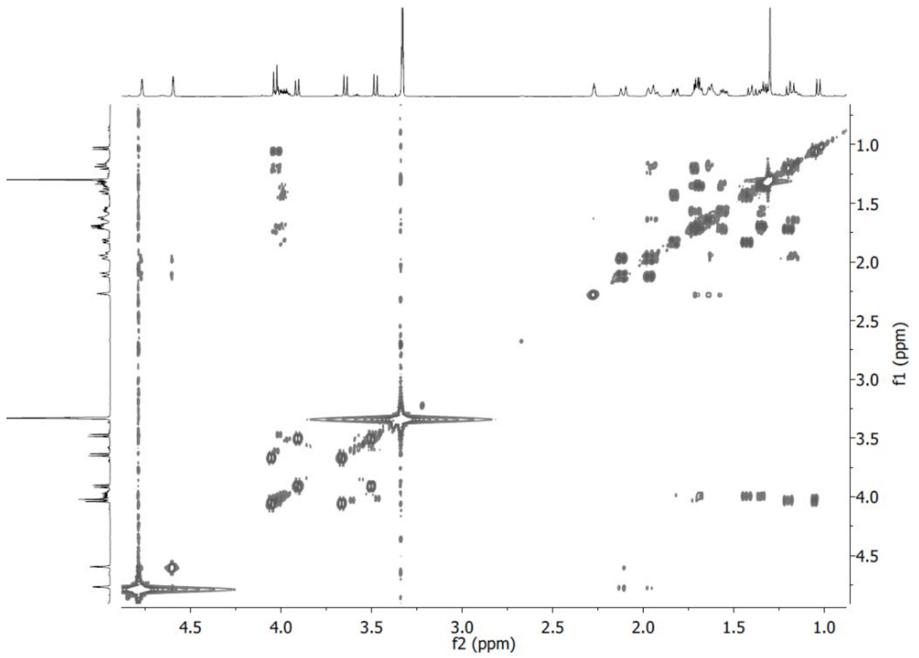

**Figure S 9.** HSQC spectrum of compound **2** ($CD_3OD$, 600 MHz)

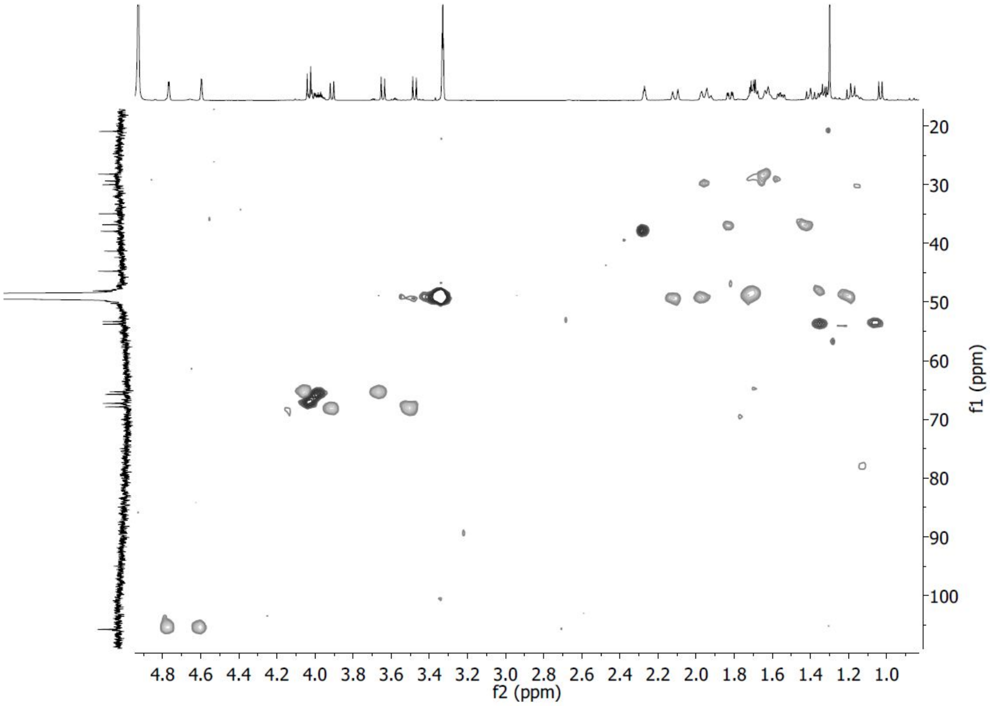


**Figure S 10.** HMBC spectrum of compound **2** ($CD_3OD$, 600 MHz)

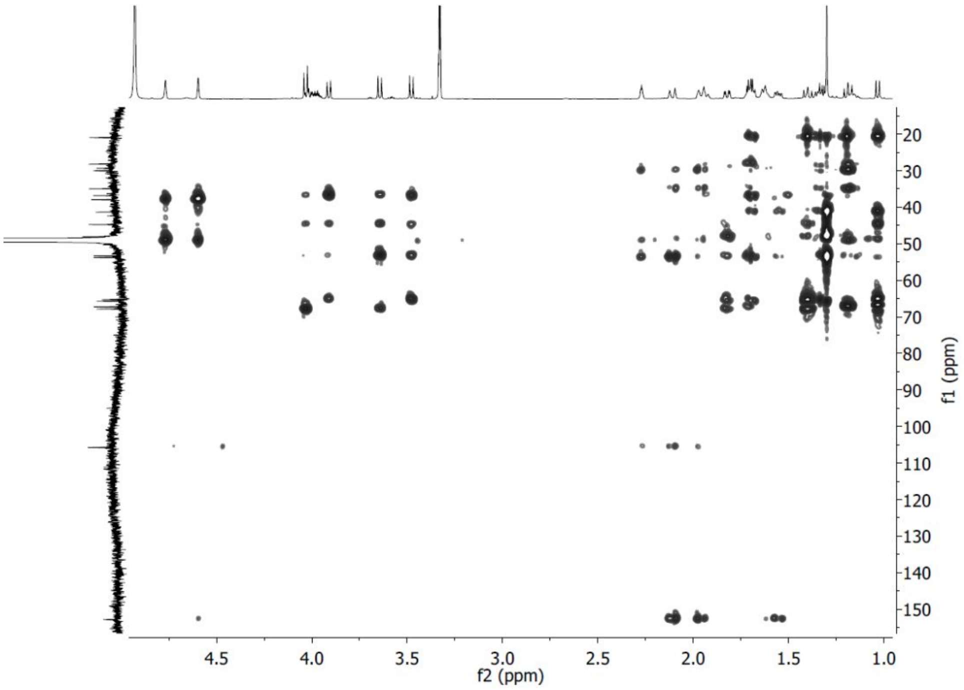

**Figure S 11.** $^{13}$C NMR spectrum of compound **2** ($CD_3OD$, 600 MHz)

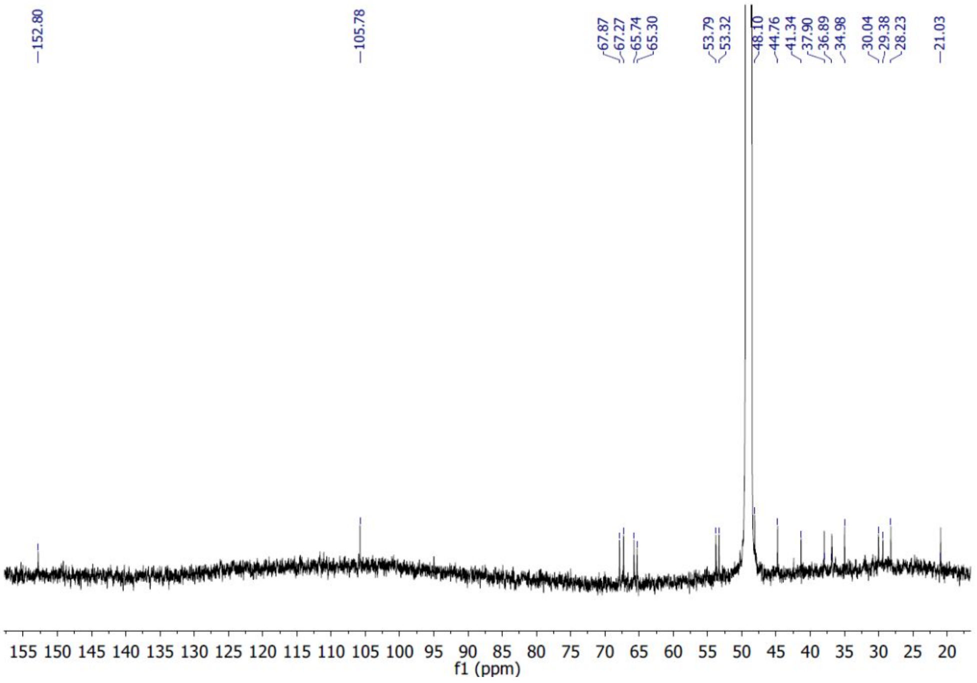


**Figure S 12.** HRESIMS of compound **2**

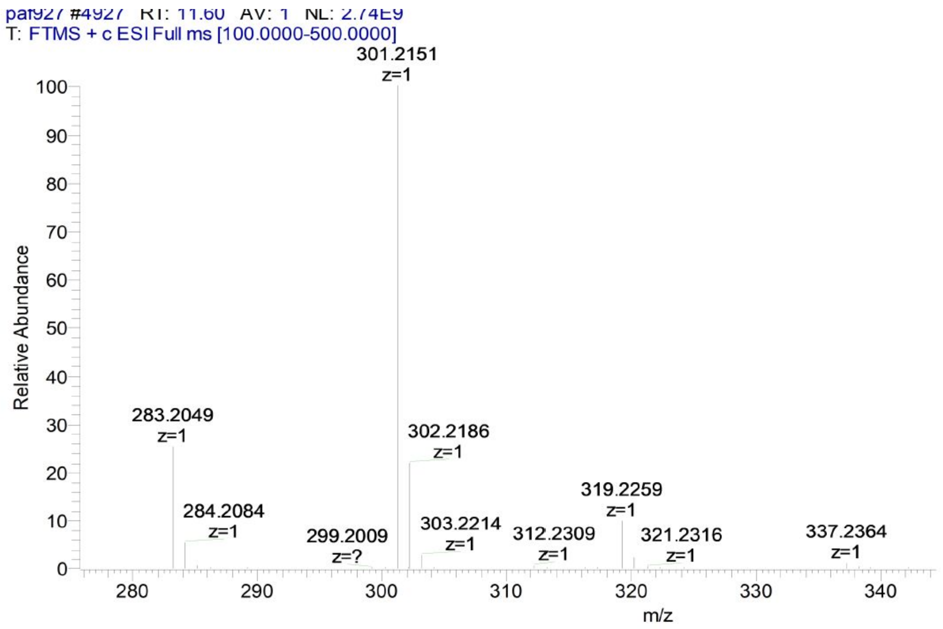

**Figure S 13.** $^{1}$H NMR spectrum of compound **3** ($CD_3OD$, 600 MHz)

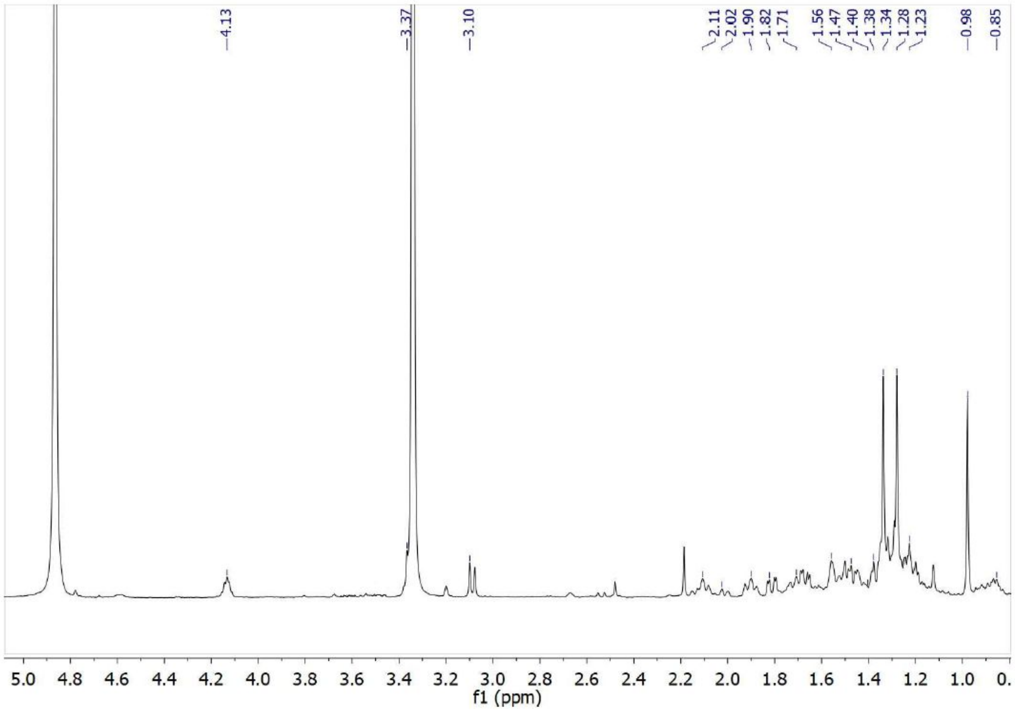


**Figure S 14.** COSY spectrum of compound **3** ($CD_3OD$, 600 MHz)

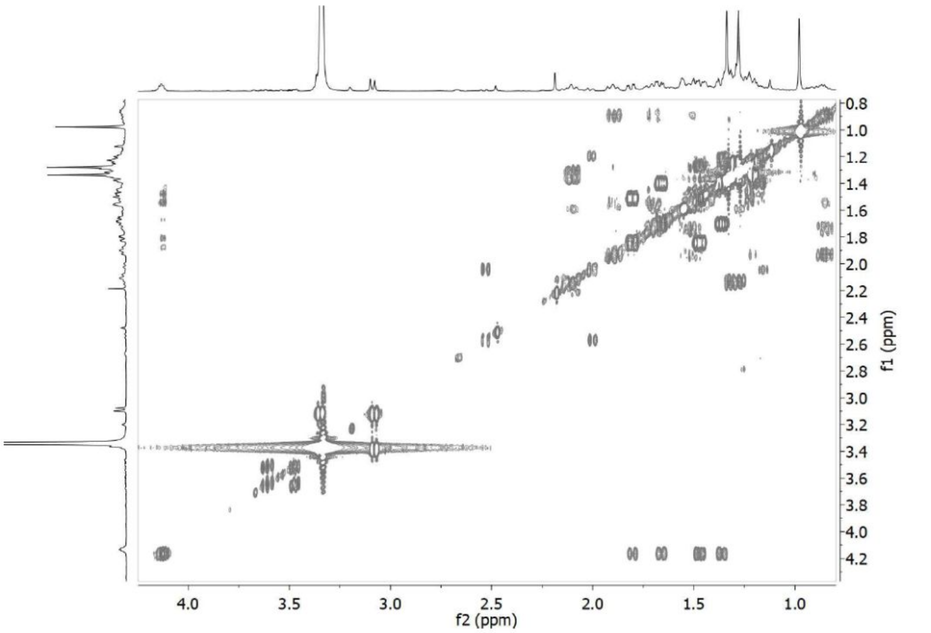

**Figure S 15.** HSQC spectrum of compound **3** ($CD_3OD$, 600 MHz)

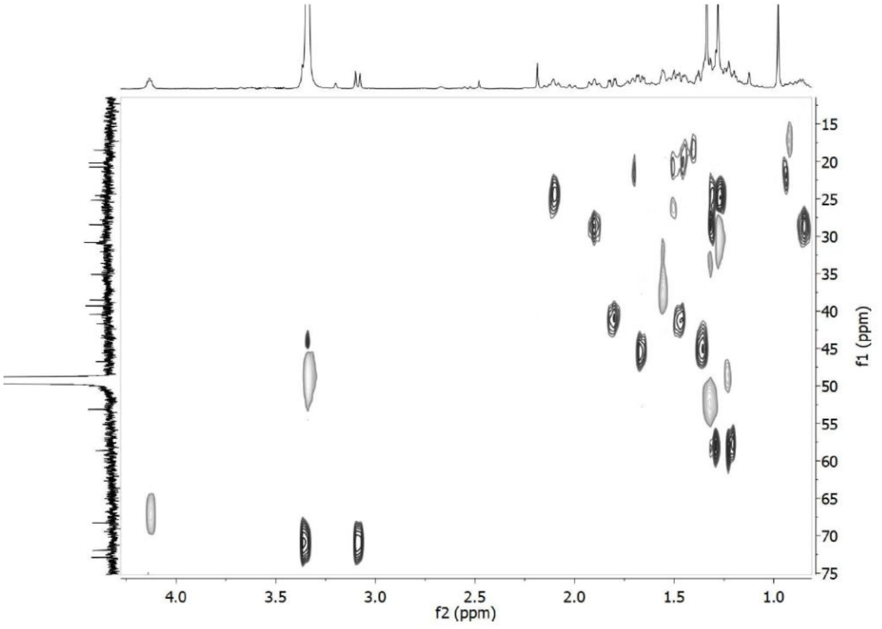


**Figure S 16.** HMBC spectrum of compound **3** ($CD_3OD$, 600 MHz)

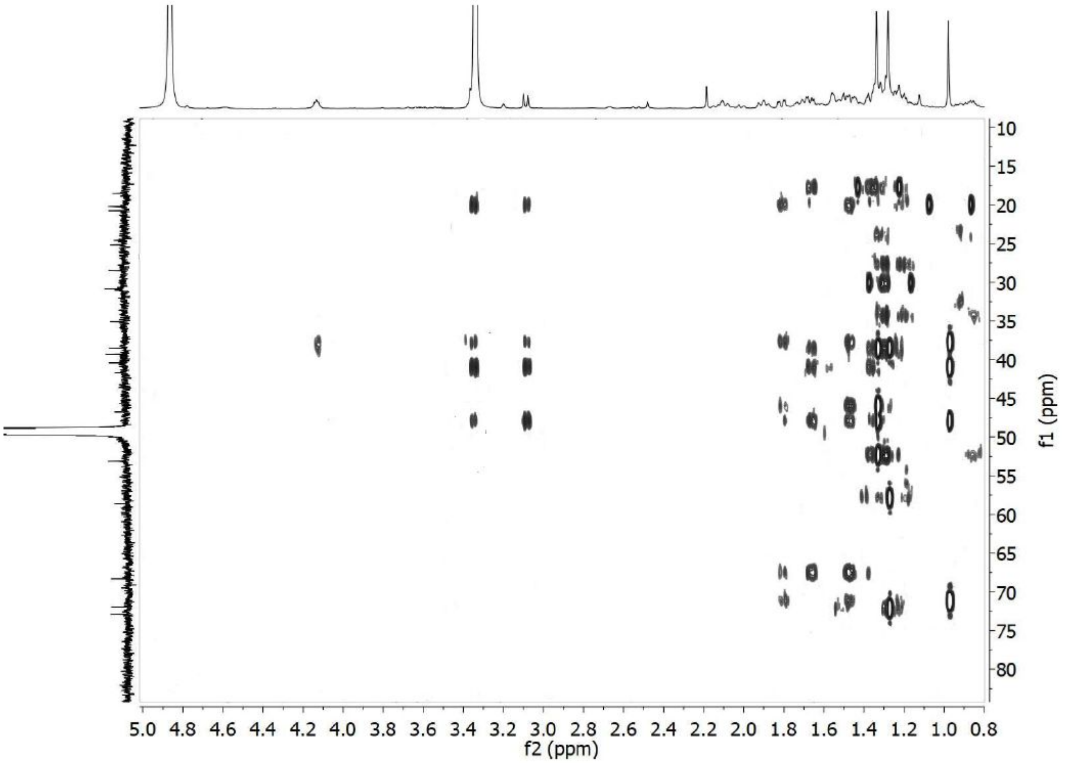

**Figure S 17.** $^{13}C$ NMR spectrum of compound **3** ($CD_3OD$, 600 MHz)

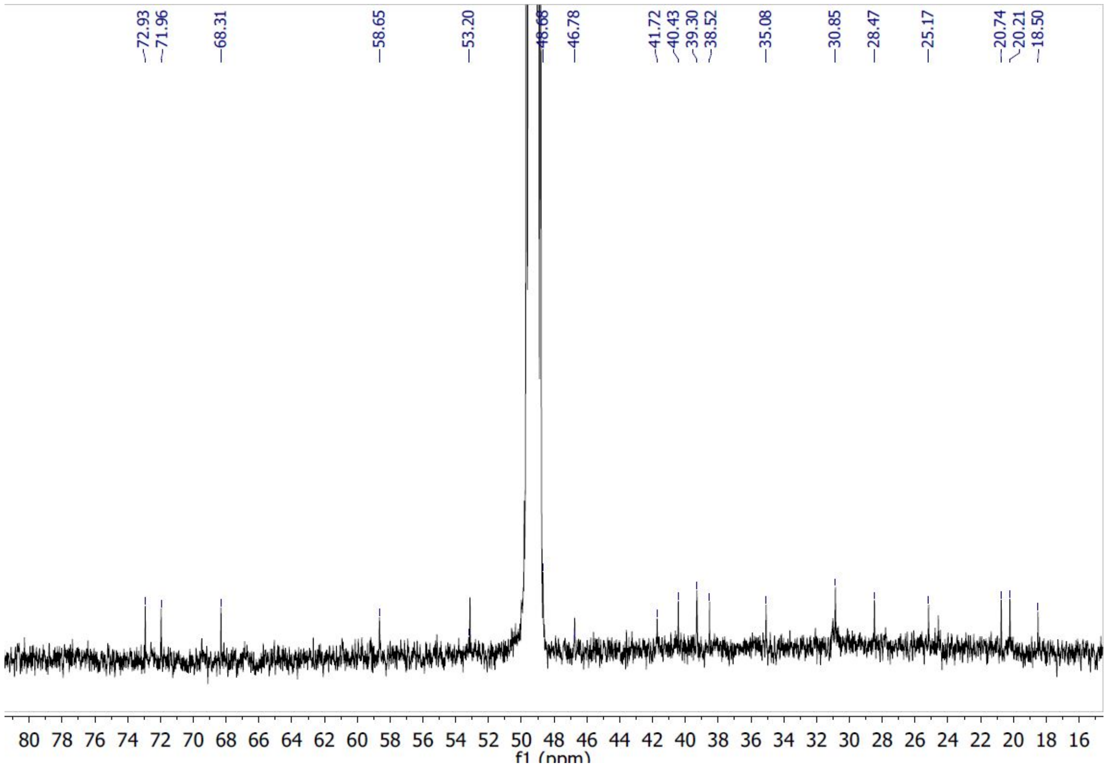


**Figure S 18.** HRESIMS of compound **3**

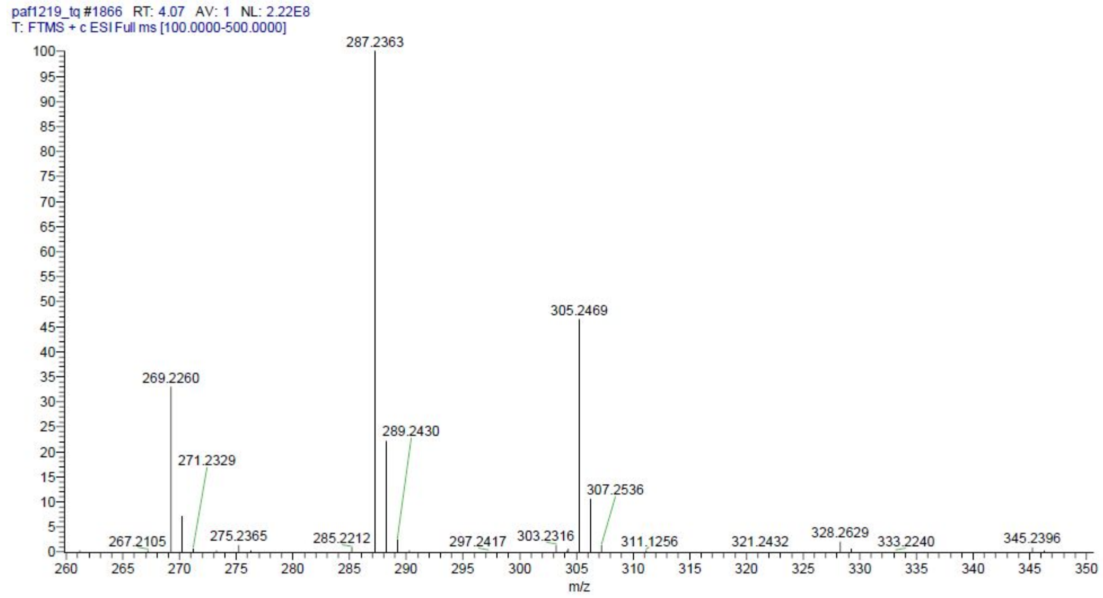

**Figure S 19.** $^{1}$H NMR spectrum of compound **4** ($CD_3OD$, 600 MHz)

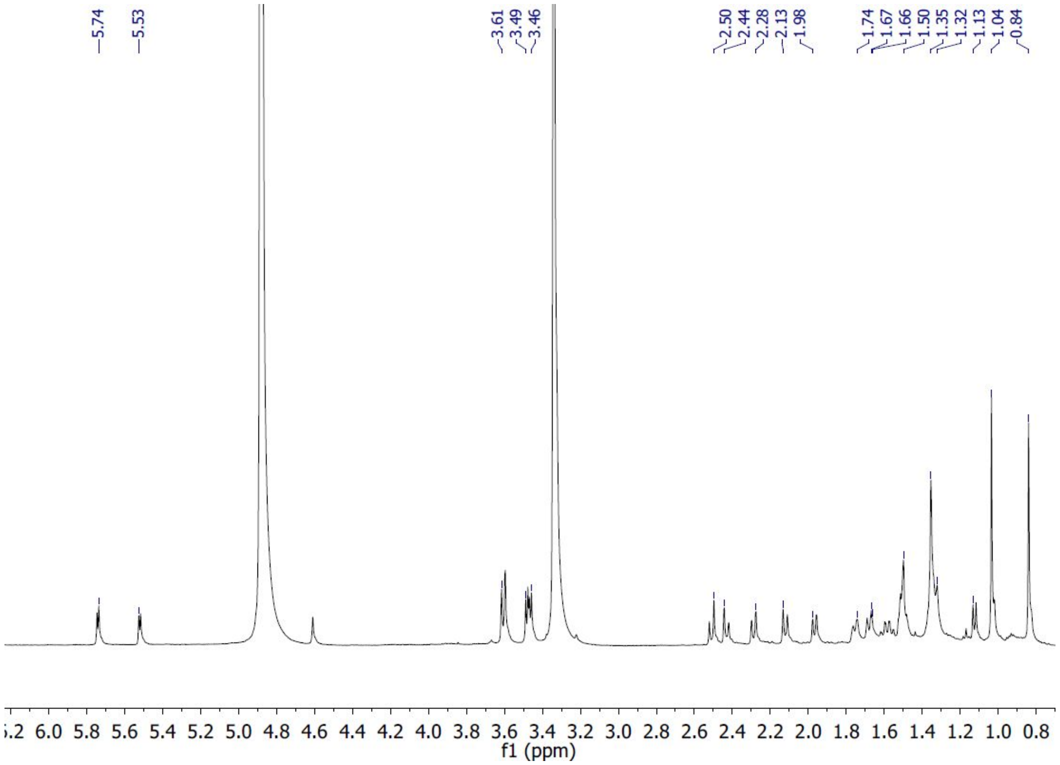


**Figure S 20.** COSY spectrum of compound **4** ($CD_3OD$, 600 MHz)

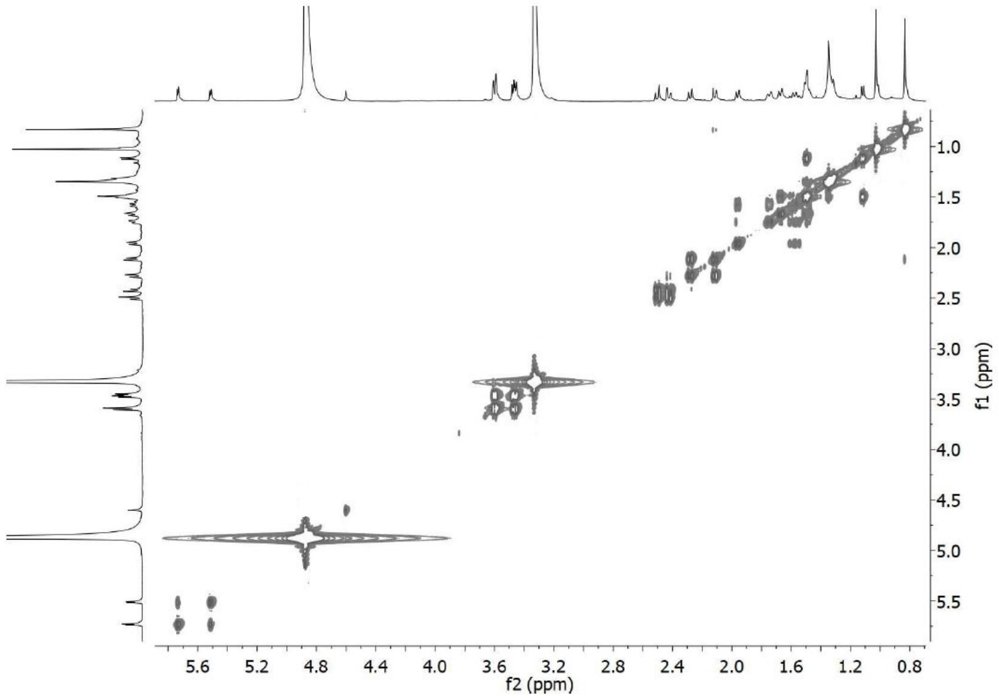

**Figure S 21.** HSQC spectrum of compound **4** ($CD_3OD$, 600 MHz)

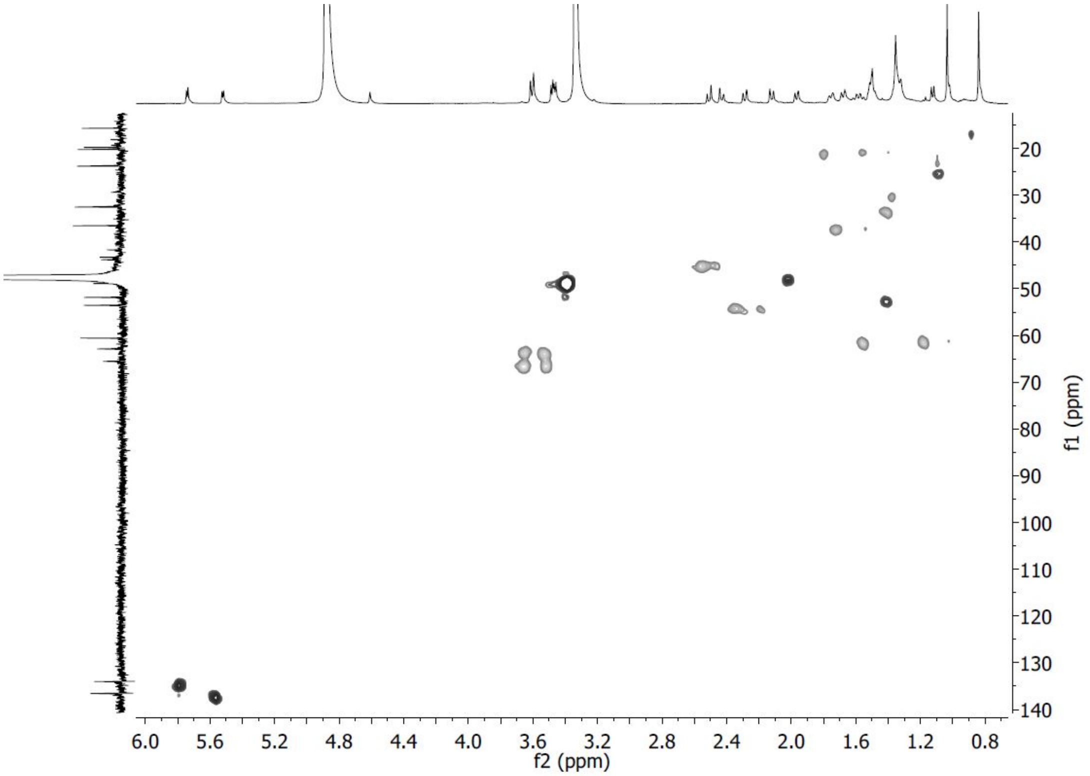


**Figure S 22.** HMBC spectrum of compound **4** ($CD_3OD$, 600 MHz)

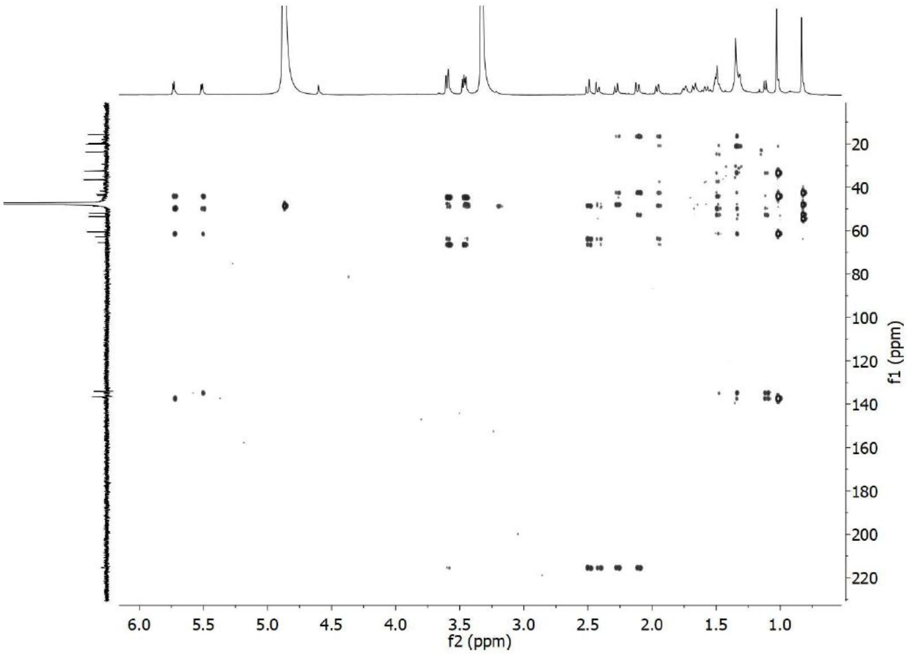

**Figure S 23.** $^{13}$C NMR spectrum of compound **4** ($CD_3OD$, 600 MHz)

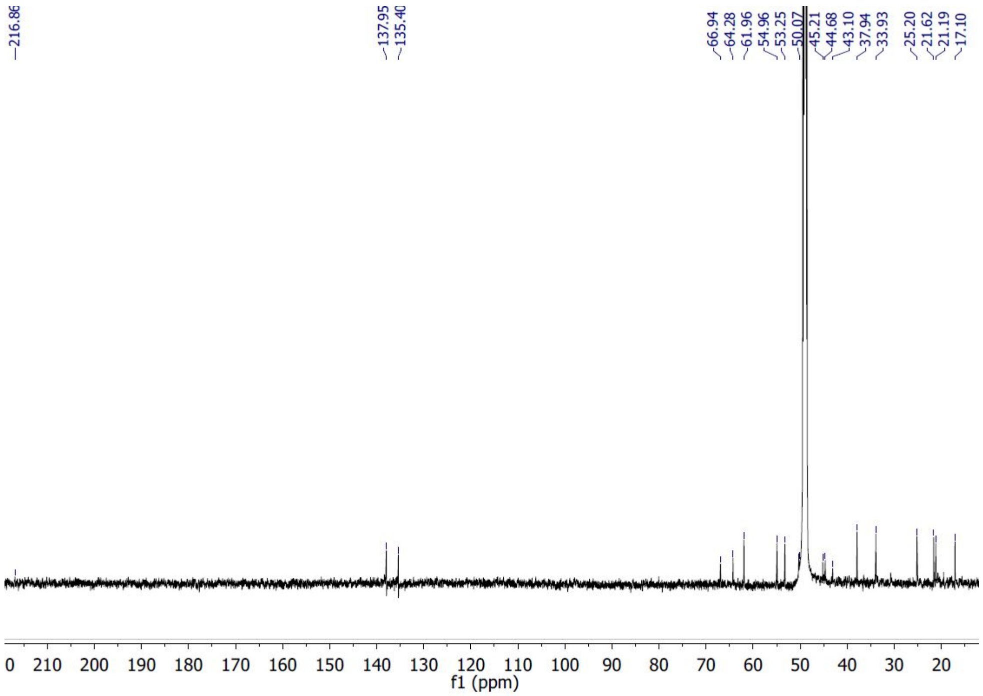


**Figure S 24.** HRESIMS of compound **4**

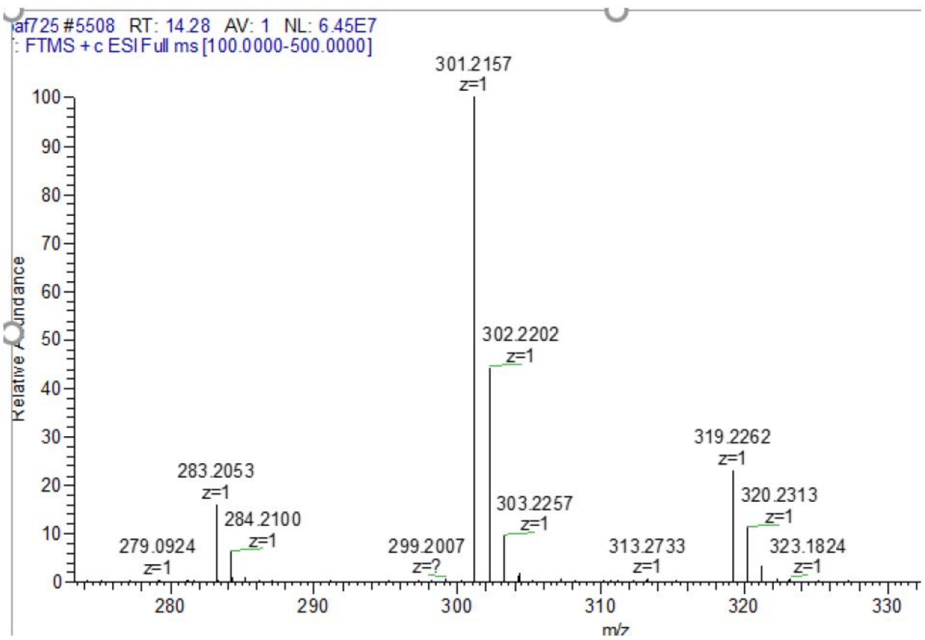

**Figure S 25.** $^{1}H$ NMR spectrum of compound **5** ($CD_3OD$, 600 MHz)

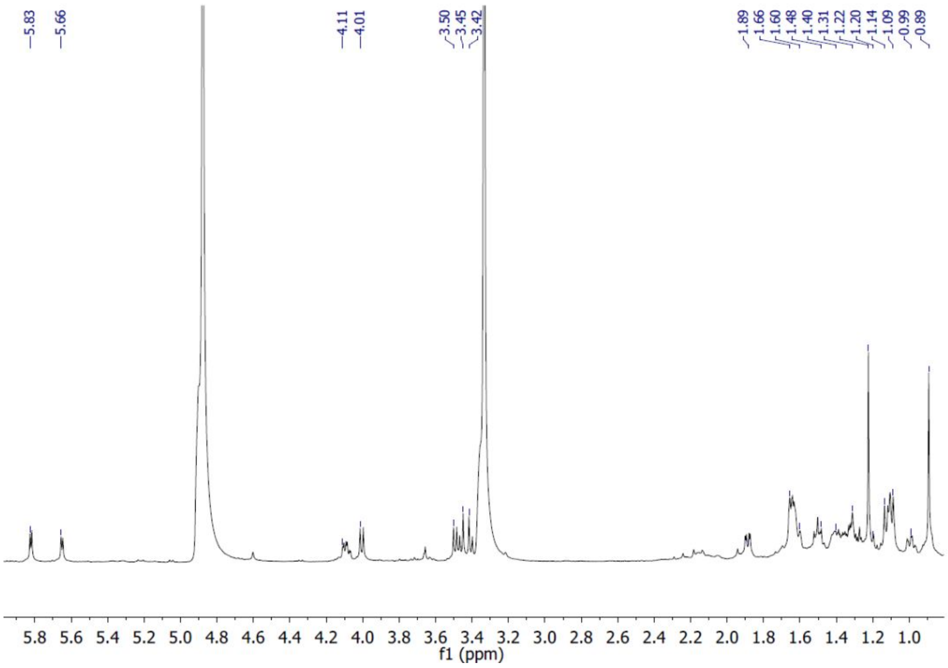


**Figure S 26.** HSQC spectrum of compound **5** ($CD_3OD$, 600 MHz)

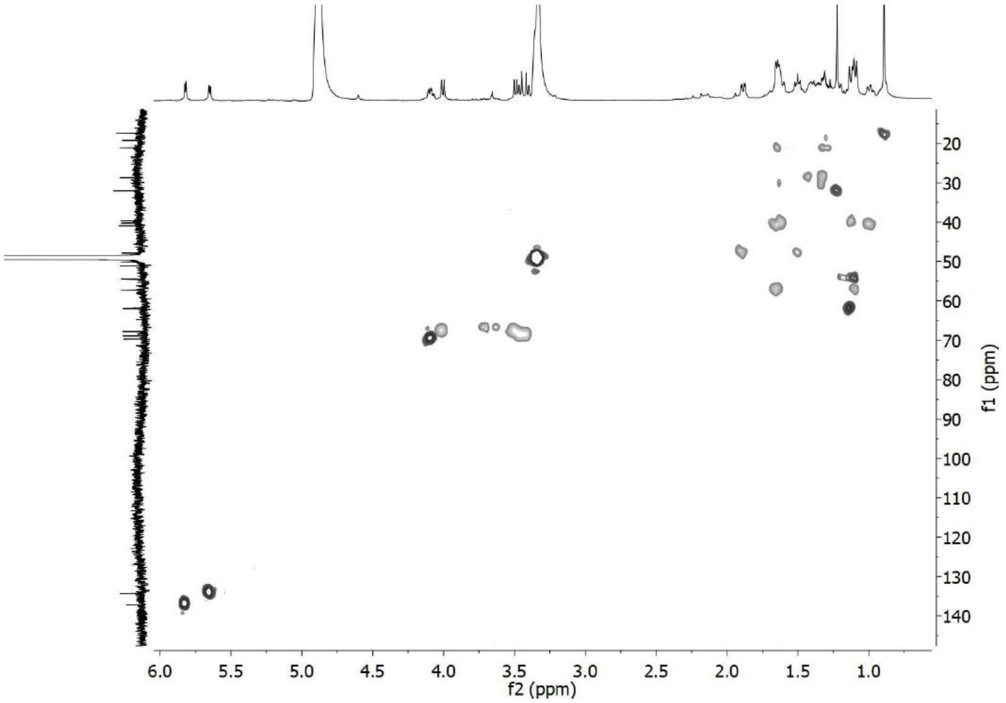

**Figure S 27.** HMBC spectrum of compound **5** ($CD_3OD$, 600 MHz)

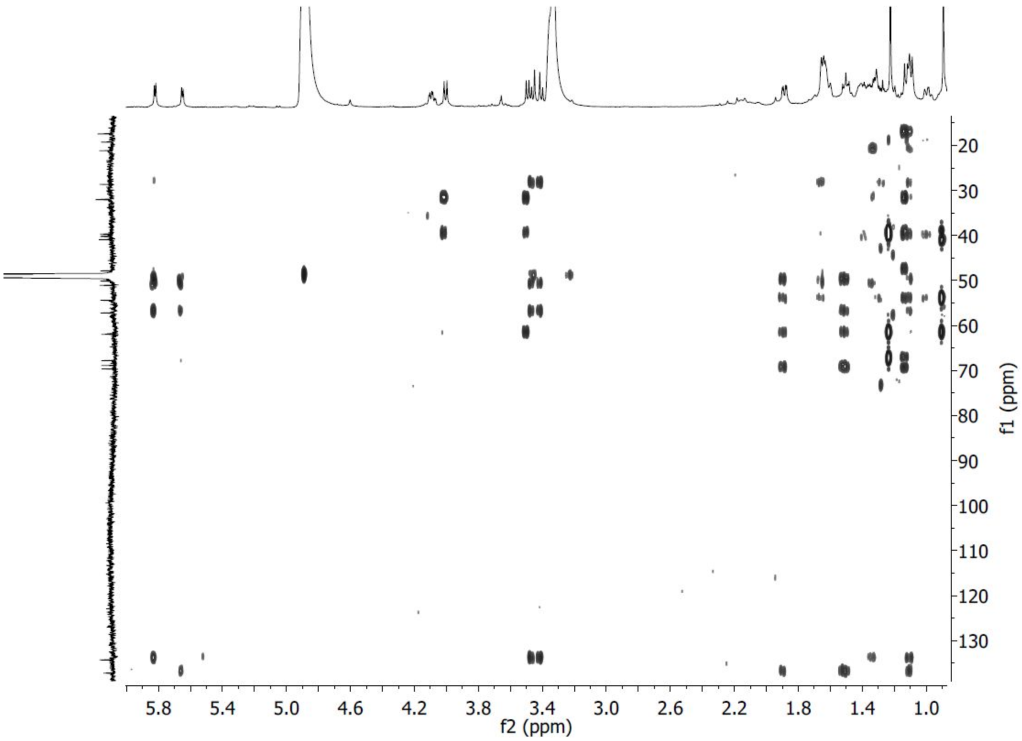


**Figure S 28.** $^{13}$C NMR spectrum of compound **5** ($CD_3OD$, 600 MHz)

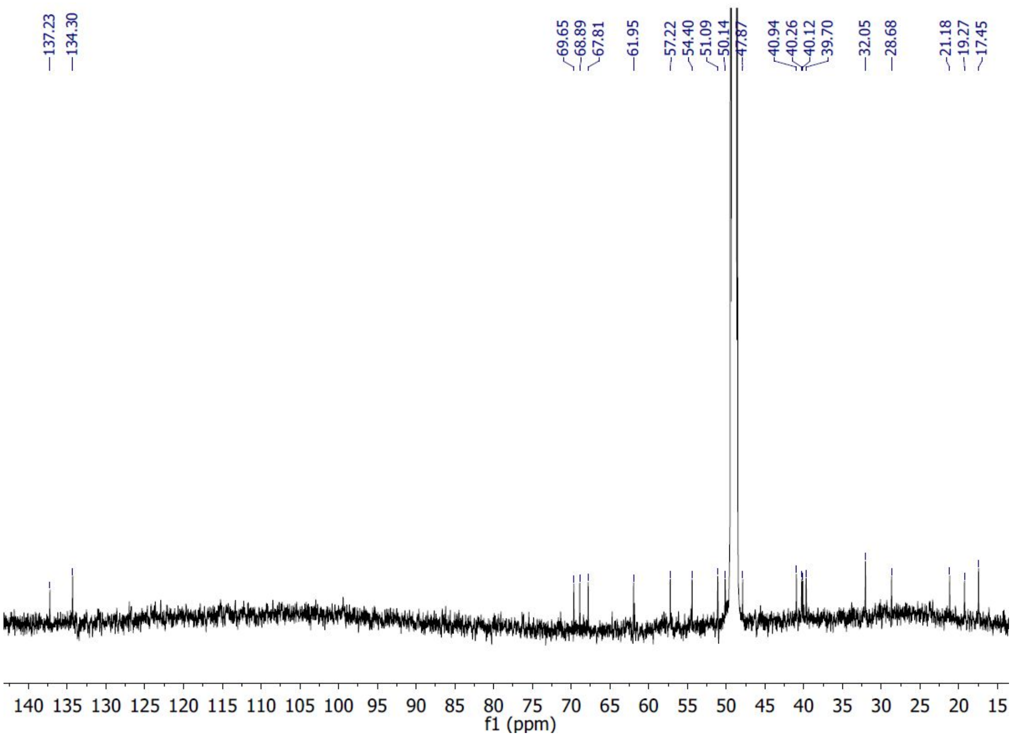

**Figure S 29.** HRESIMS of compound **5**

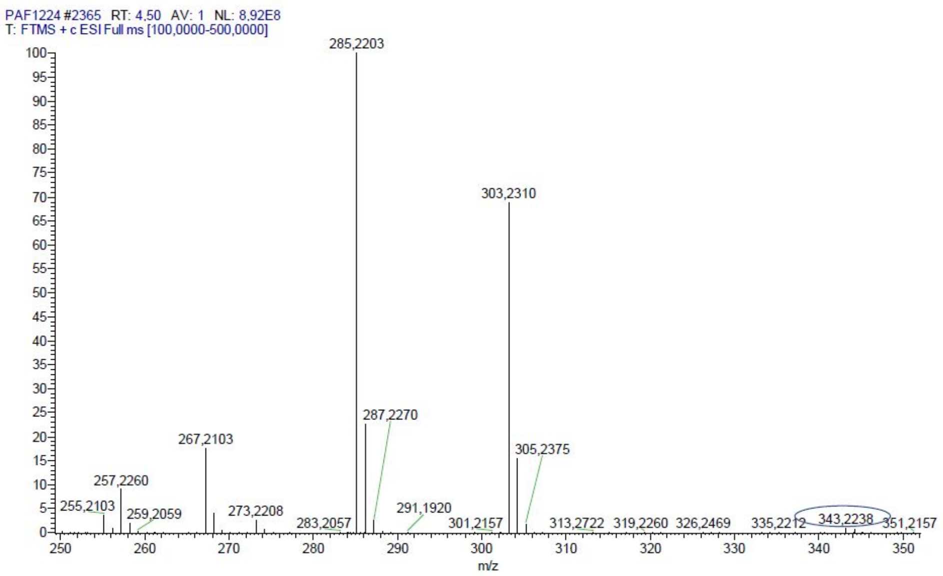


**Figure S 30.** $^{1}$H NMR spectrum of compound **6** ($CD_3OD$, 600 MHz)

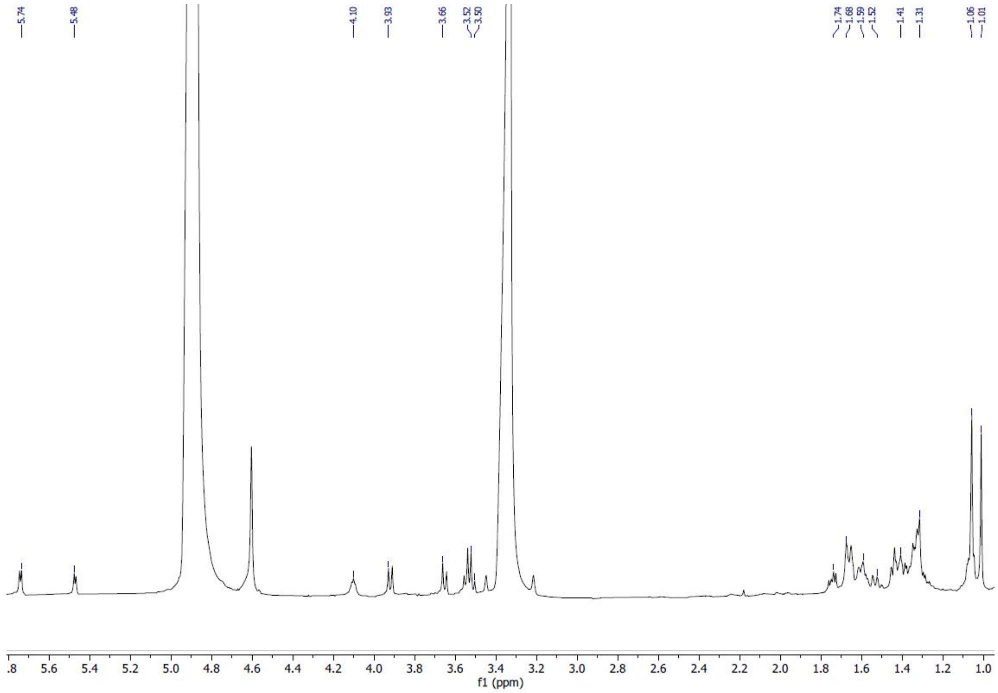

**Figure S 31.** COSY spectrum of compound **6** ($CD_3OD$, 600 MHz)

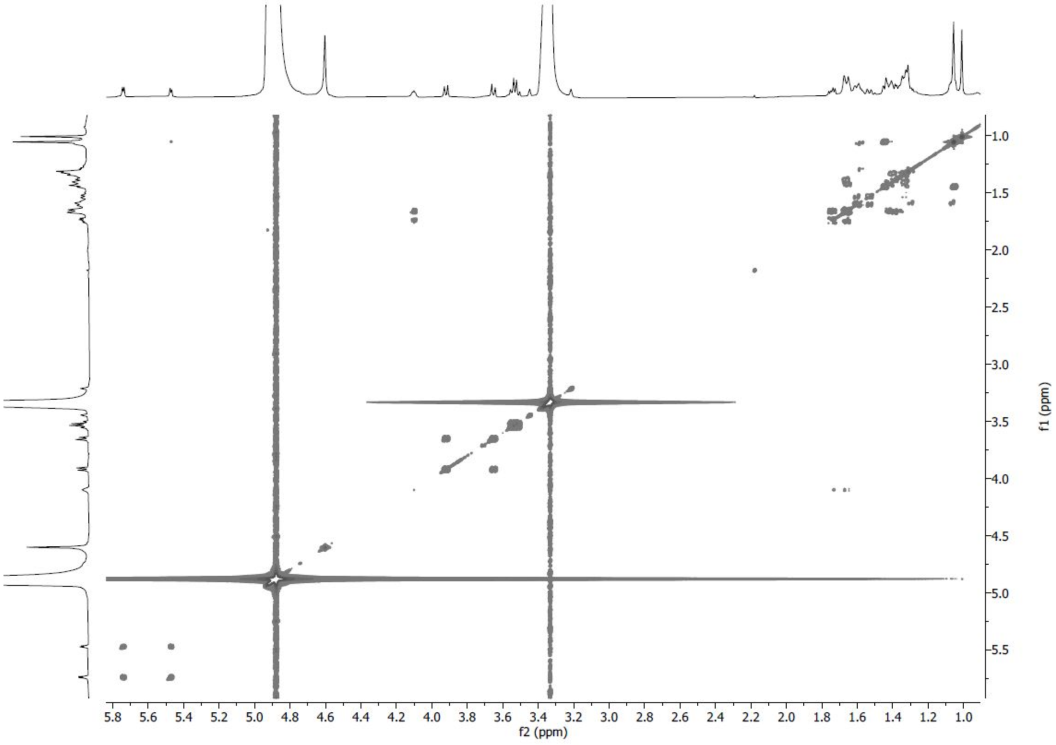


**Figure S 32.** HSQC spectrum of compound **6** ($CD_3OD$, 600 MHz)

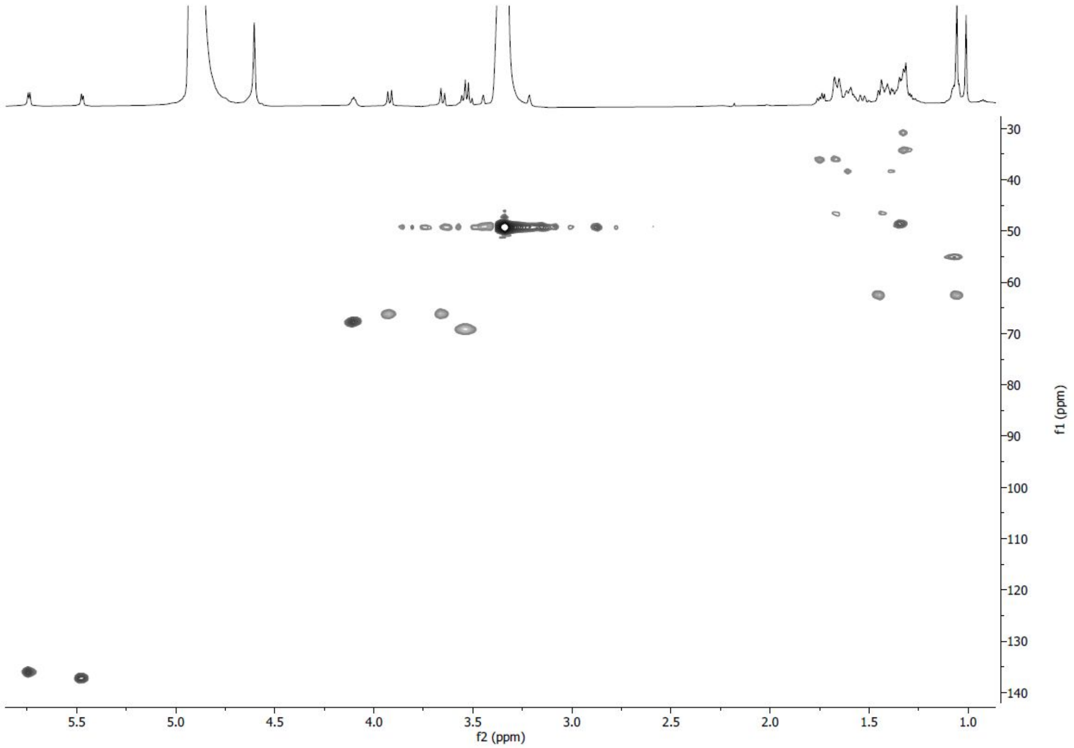

**Figure S 33.** HMBC spectrum of compound **6** ($CD_3OD$, 600 MHz)

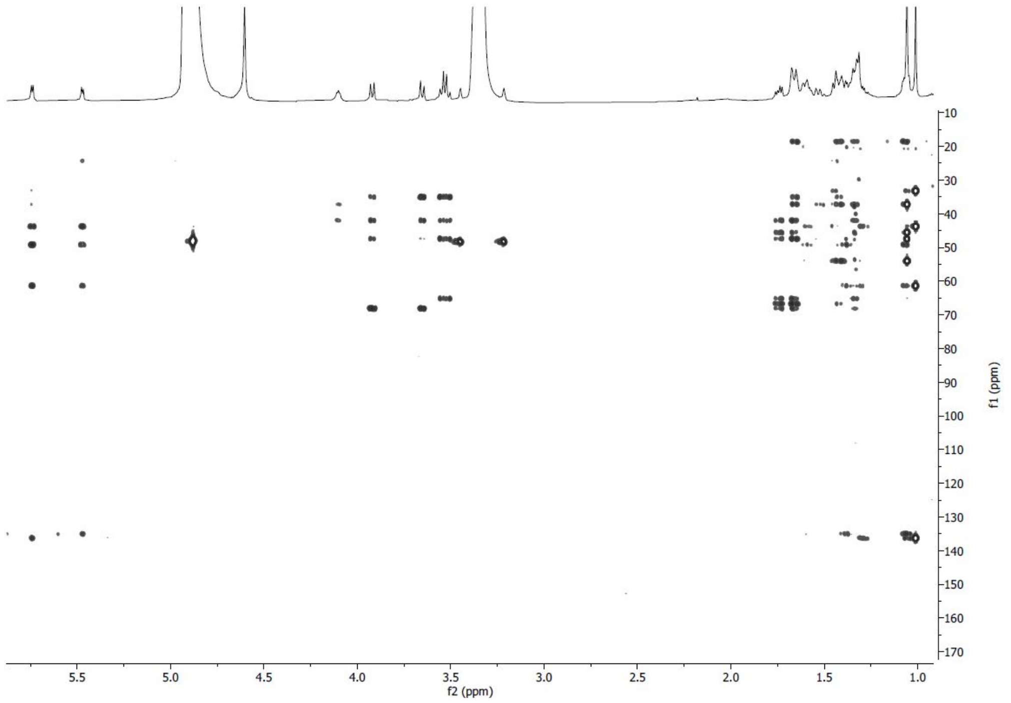


**Figure S 34.** HRESIMS of compound **6**

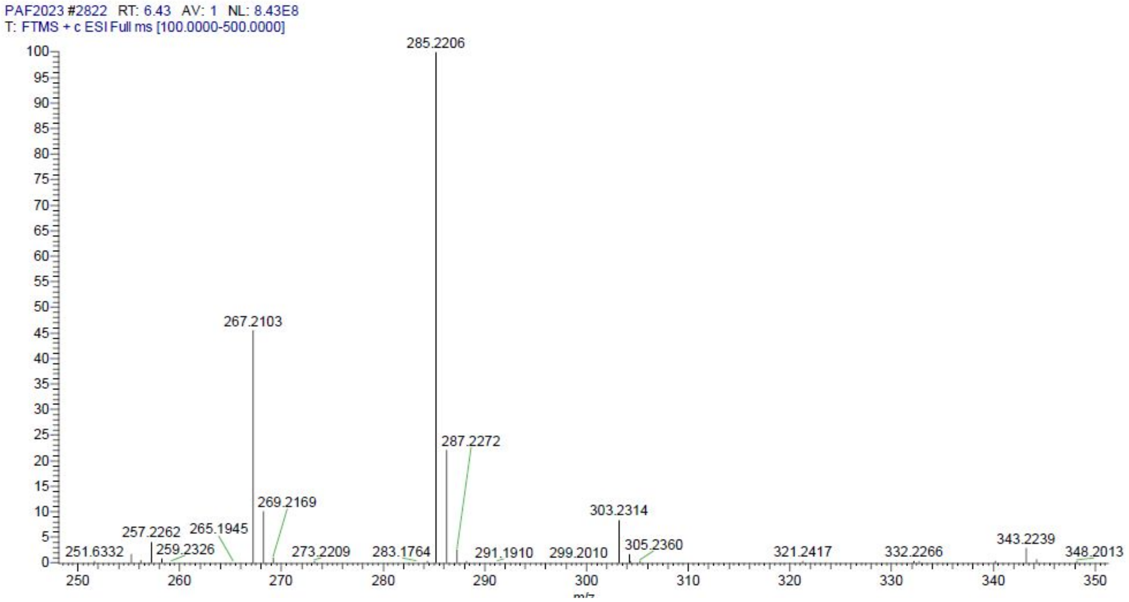

**Figure S 35.** $^{1}H$ NMR spectrum of compound **7** ($CD_3OD$, 600 MHz)

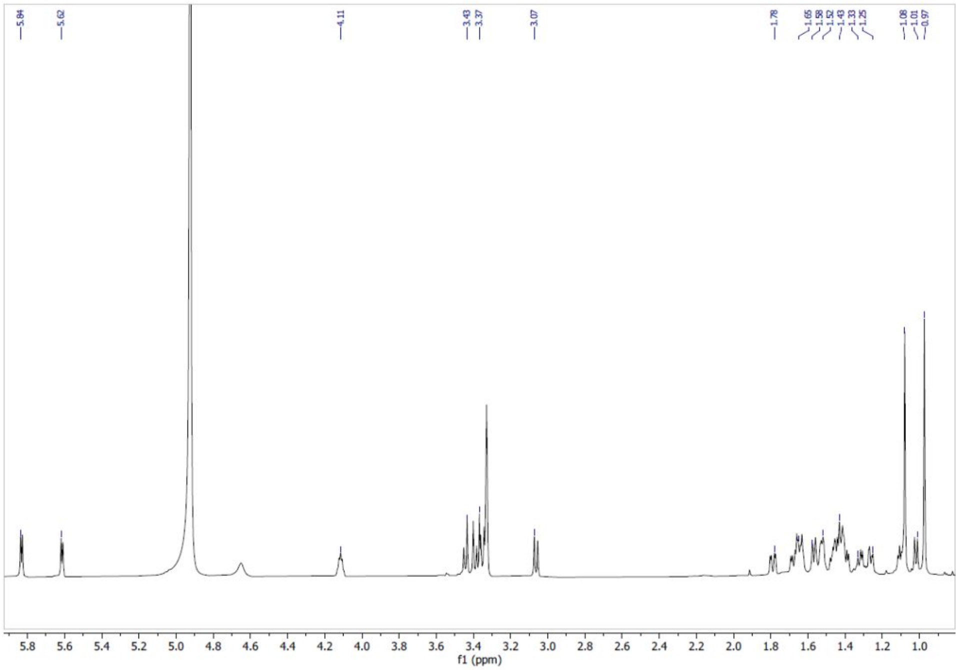

**Figure S 36.** COSY spectrum of compound **7** ($CD_3OD$, 600 MHz)

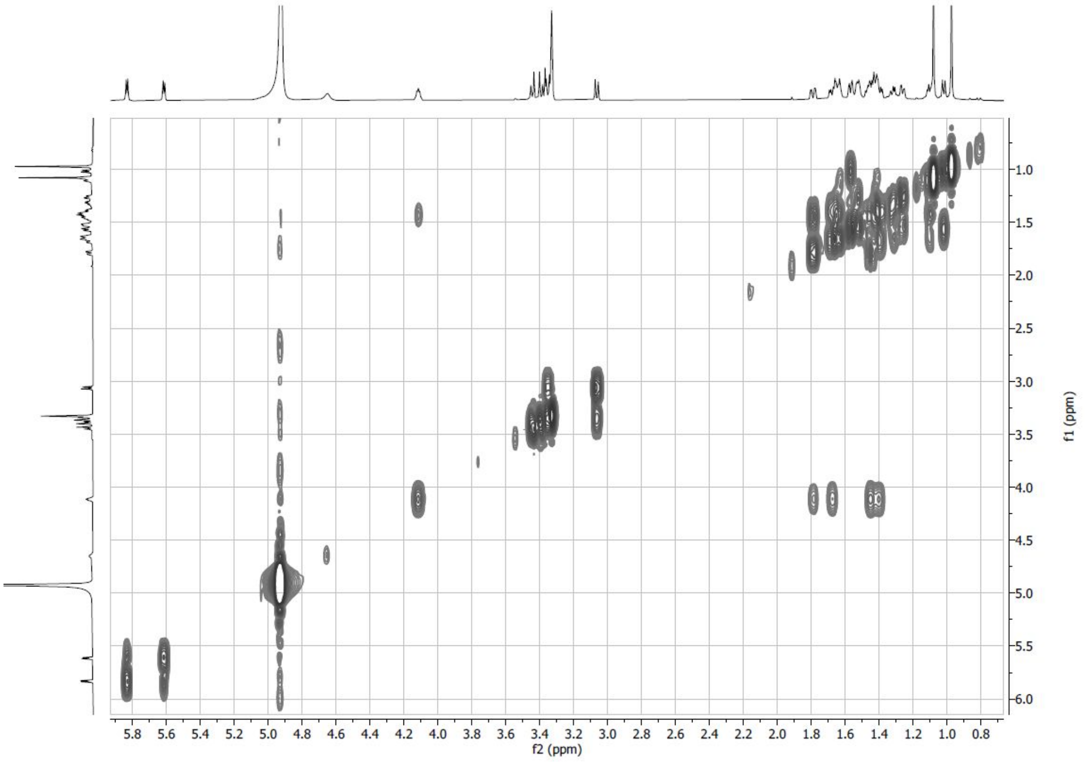

**Figure S 37.** HSQC spectrum of compound **7** ($CD_3OD$, 600 MHz)

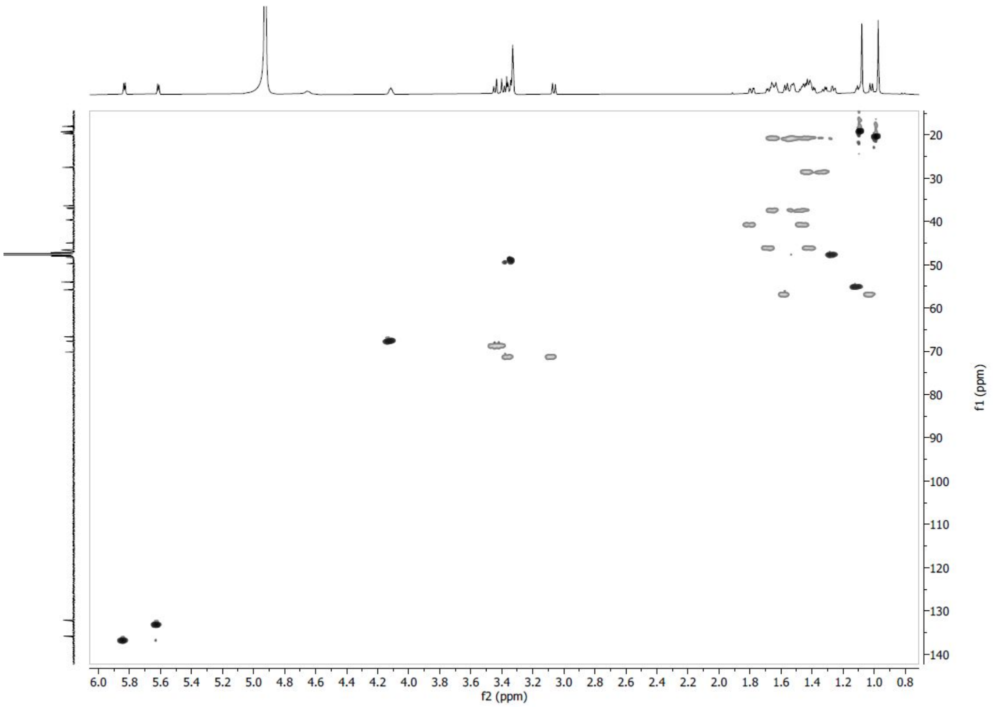


**Figure S 38.** HMBC spectrum of compound **7** ($CD_3OD$, 600 MHz)

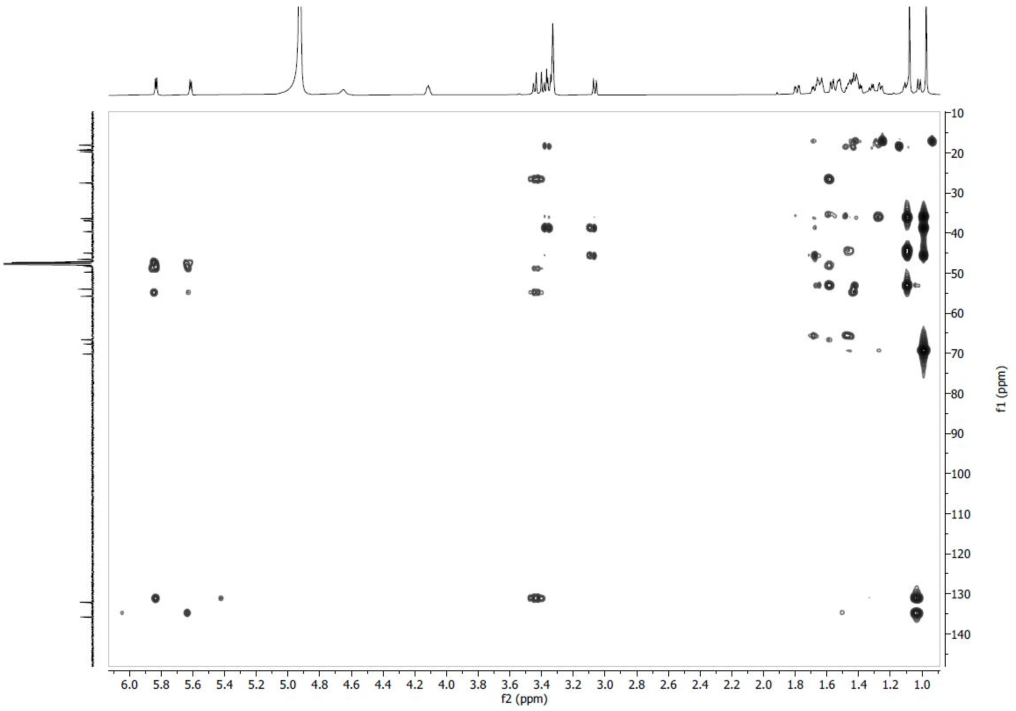

**Figure S 39.** $^{13}C$ NMR spectrum of compound **7** ($CD_3OD$, 600 MHz)

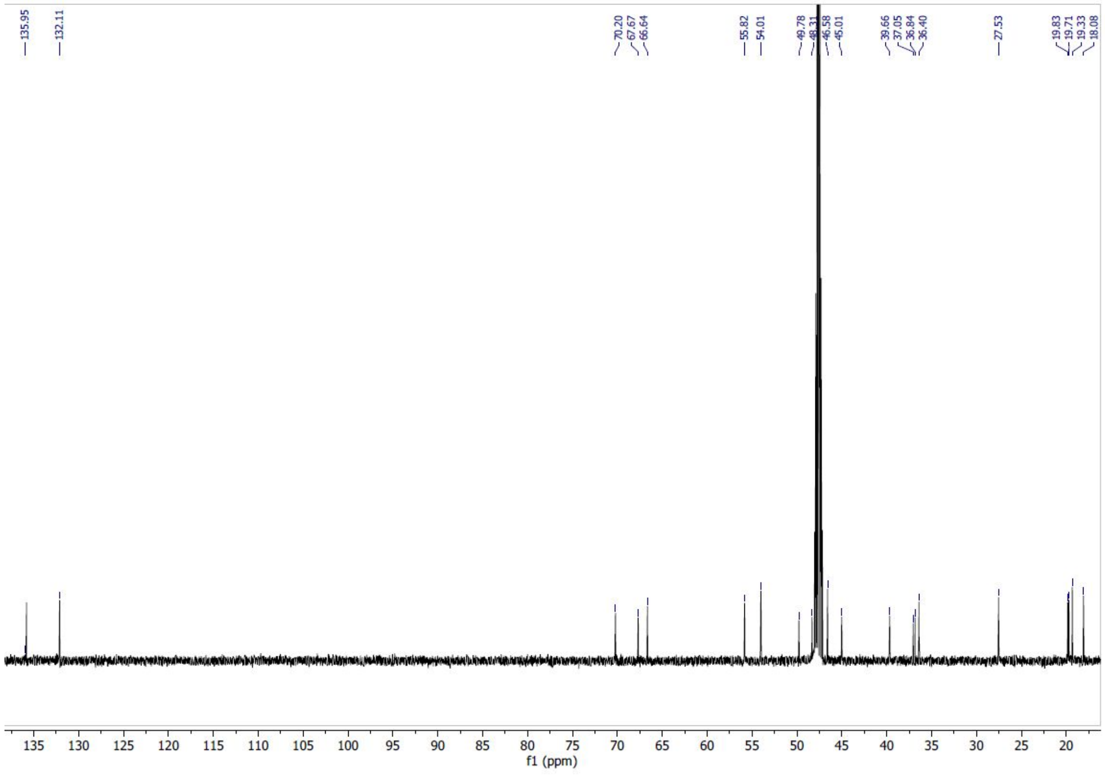

**Figure S 40.** HRESIMS of compound **7**

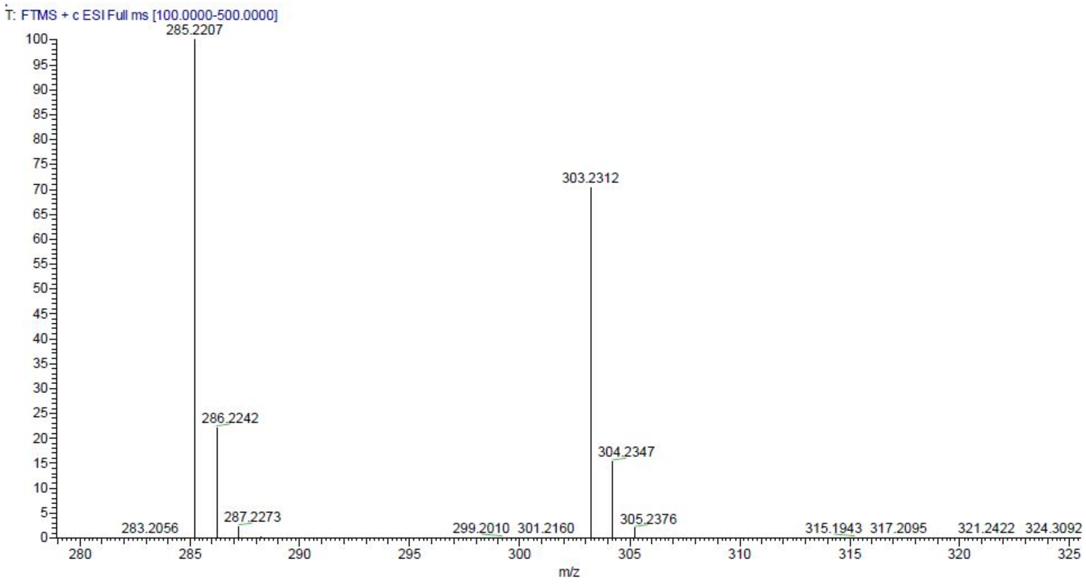

**Figure S 41.** $^{1}$H NMR spectrum of compound **8** ($CD_3OD$, 600 MHz)

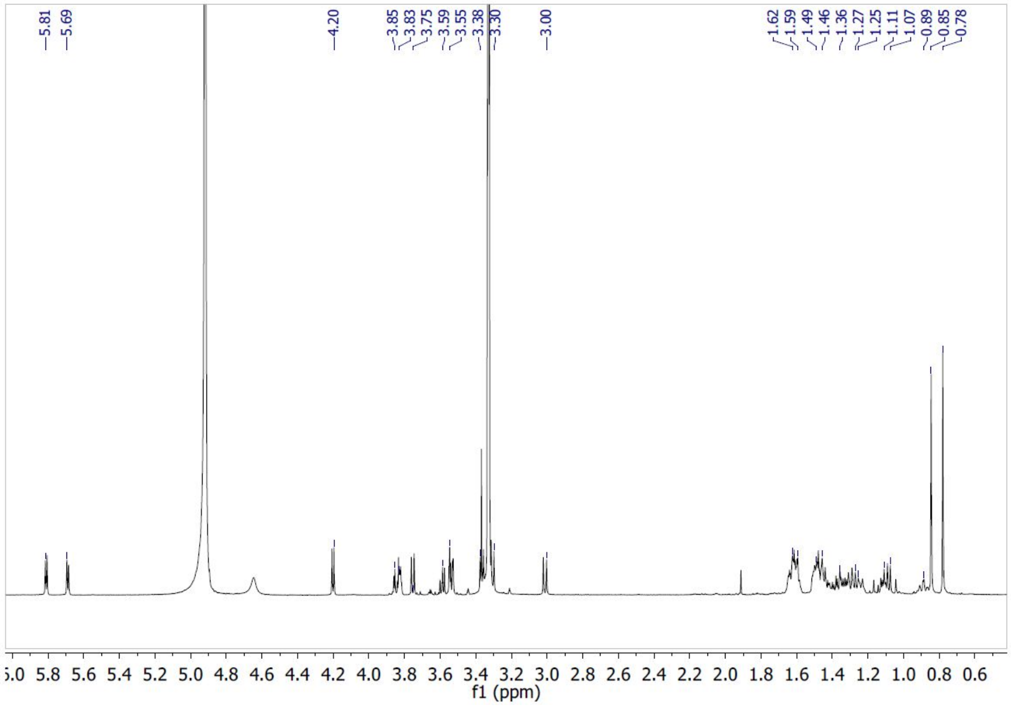


**Figure S 42.** COSY spectrum of compound **8** ($CD_3OD$, 600 MHz)

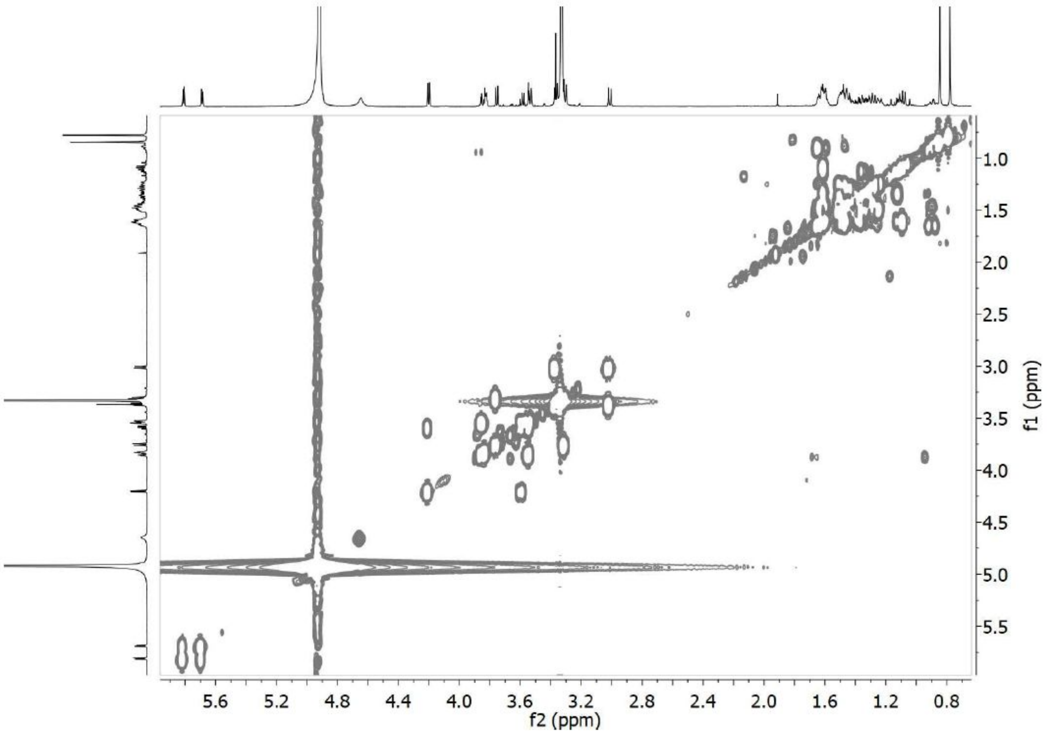

**Figure S 43.** HSQC spectrum of compound **8** ($CD_3OD$, 600 MHz)

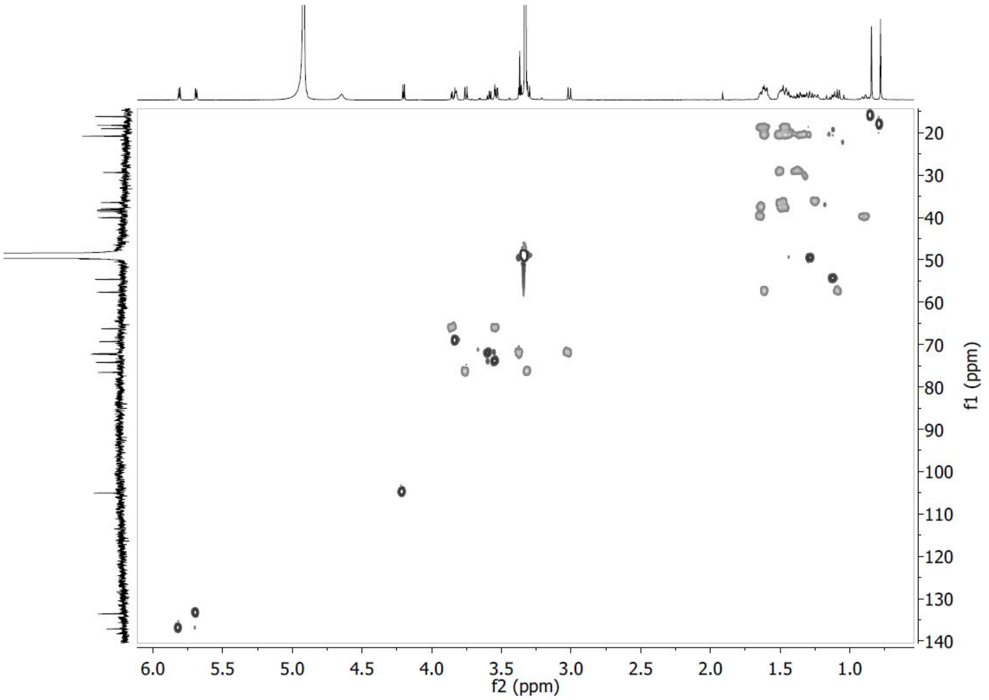


**Figure S 44.** HMBC spectrum of compound **8** ($CD_3OD$, 600 MHz)

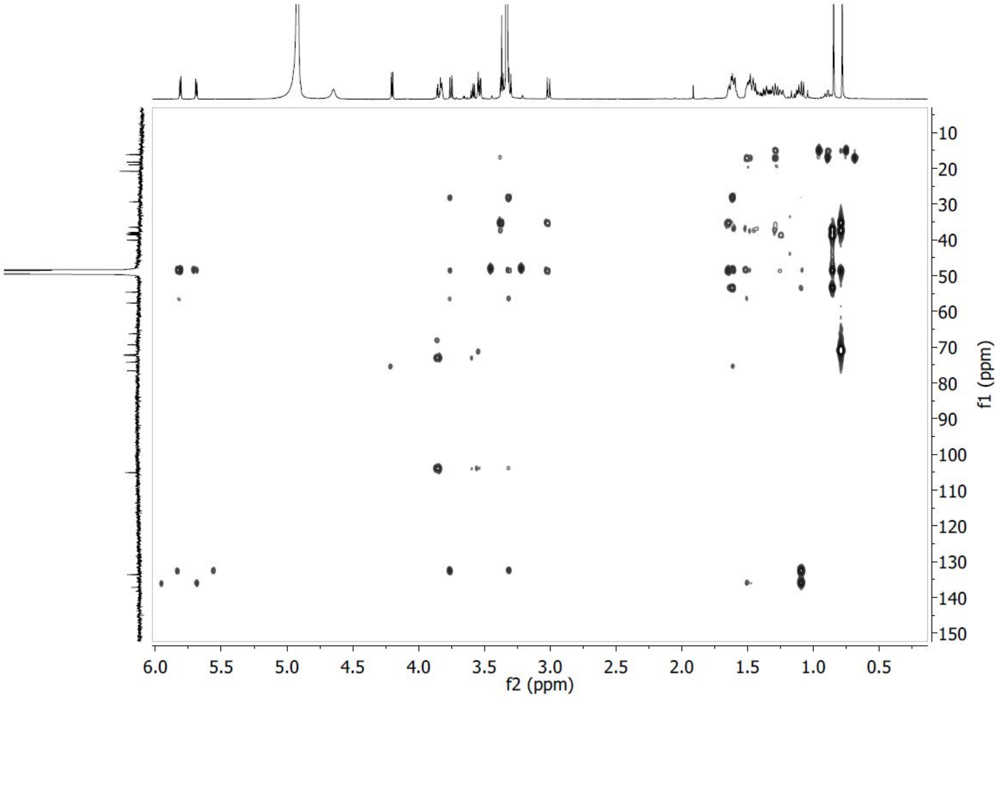

**Figure S 45.** $^{13}C$ NMR spectrum of compound **8** ($CD_3OD$, 600 MHz)

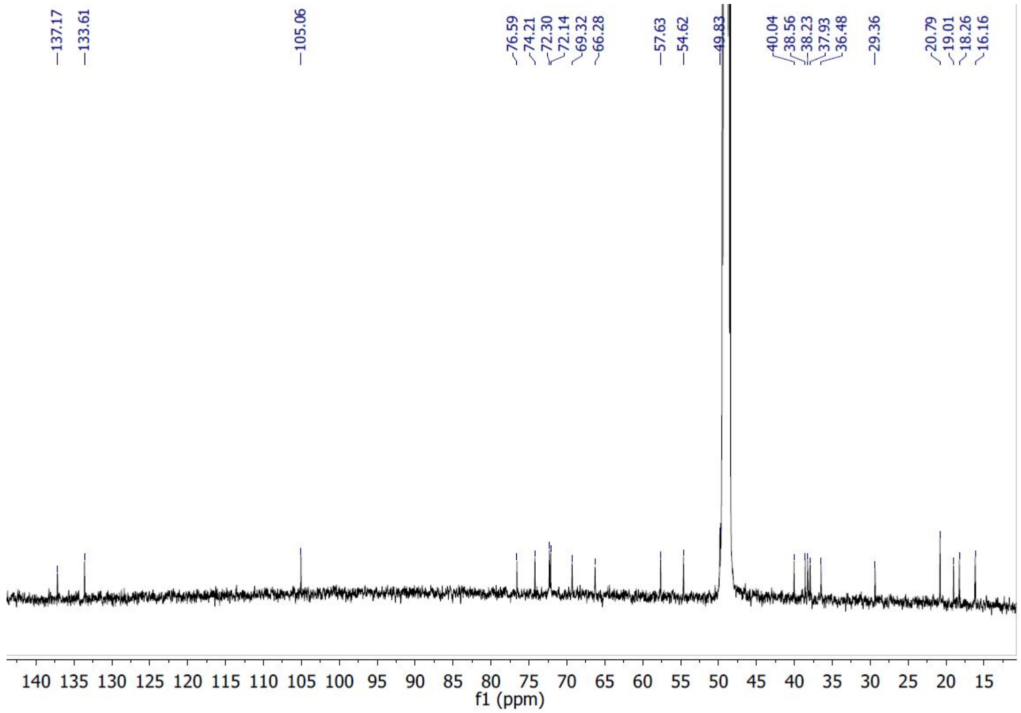


**Figure S 46.** HRESIMS of compound **8**

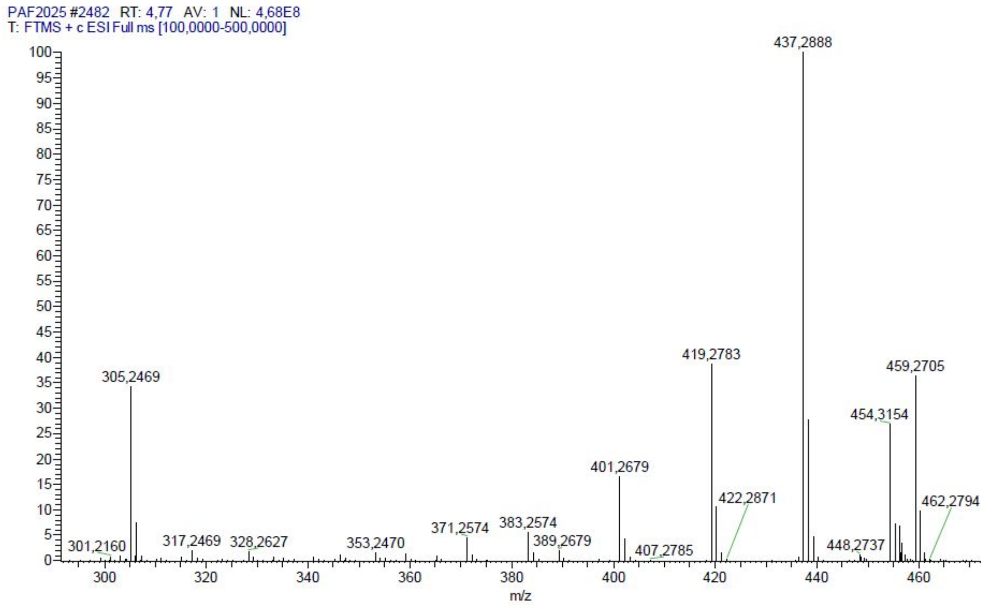

**Figure S 47.** $^1$H NMR spectrum of compound **9** ($CD_3OD$, 600 MHz).

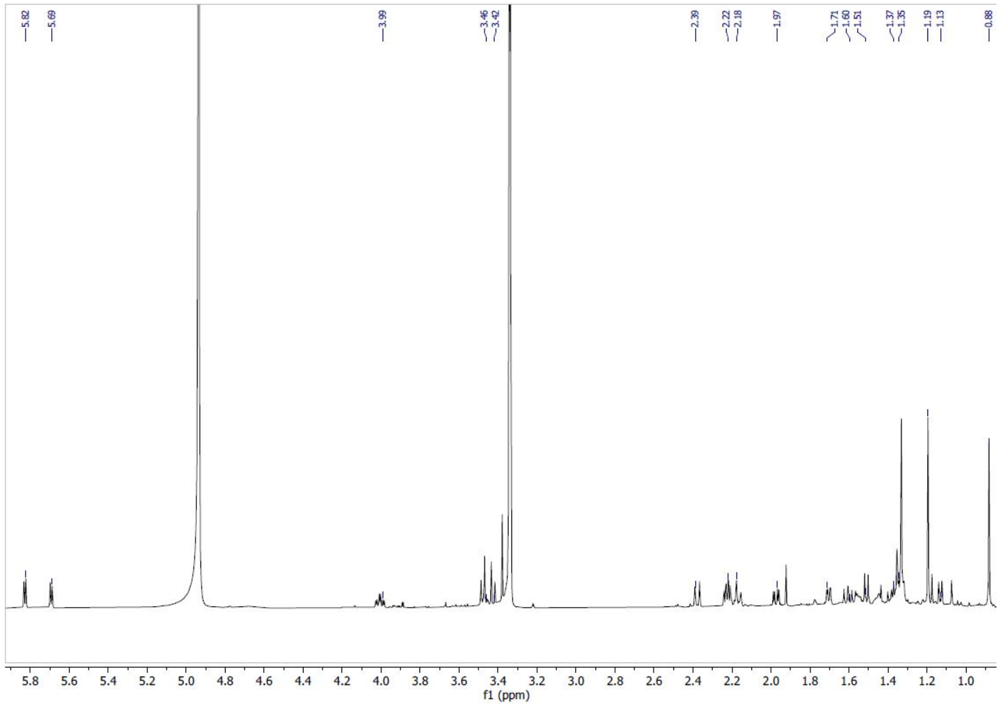


**Figure S 48.** COSY spectrum of compound **9** ($CD_3OD$, 600 MHz)

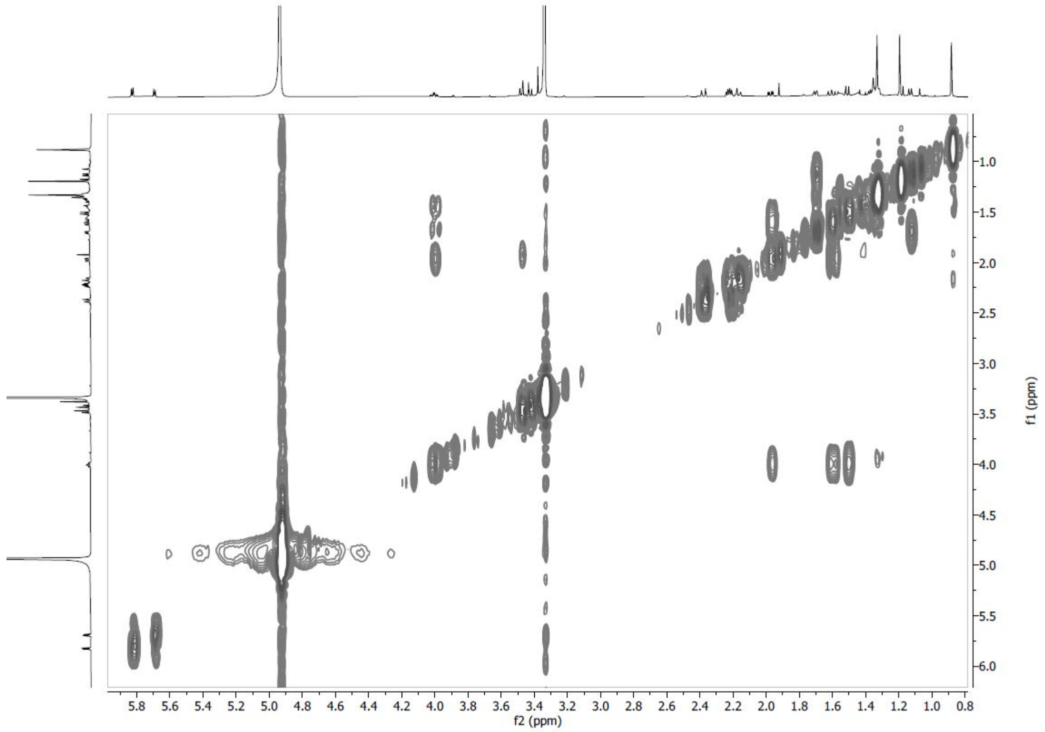

**Figure S 49.** HSQC spectrum of compound **9** ($CD_3OD$, 600 MHz)

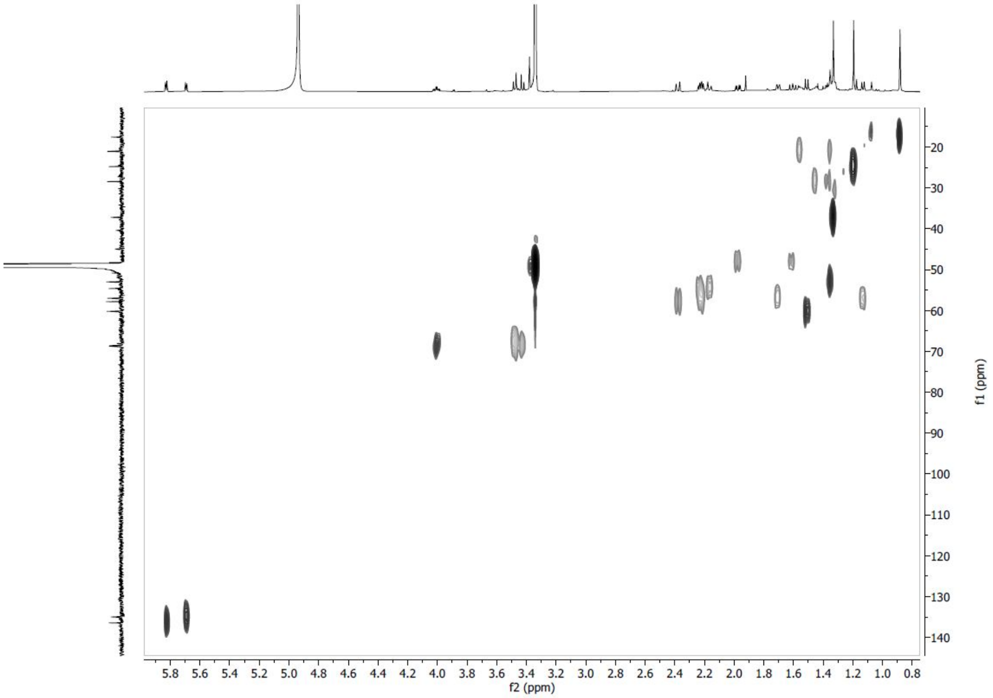


**Figure S 50.** HMBC spectrum of compound **9** ($CD_3OD$, 600 MHz)

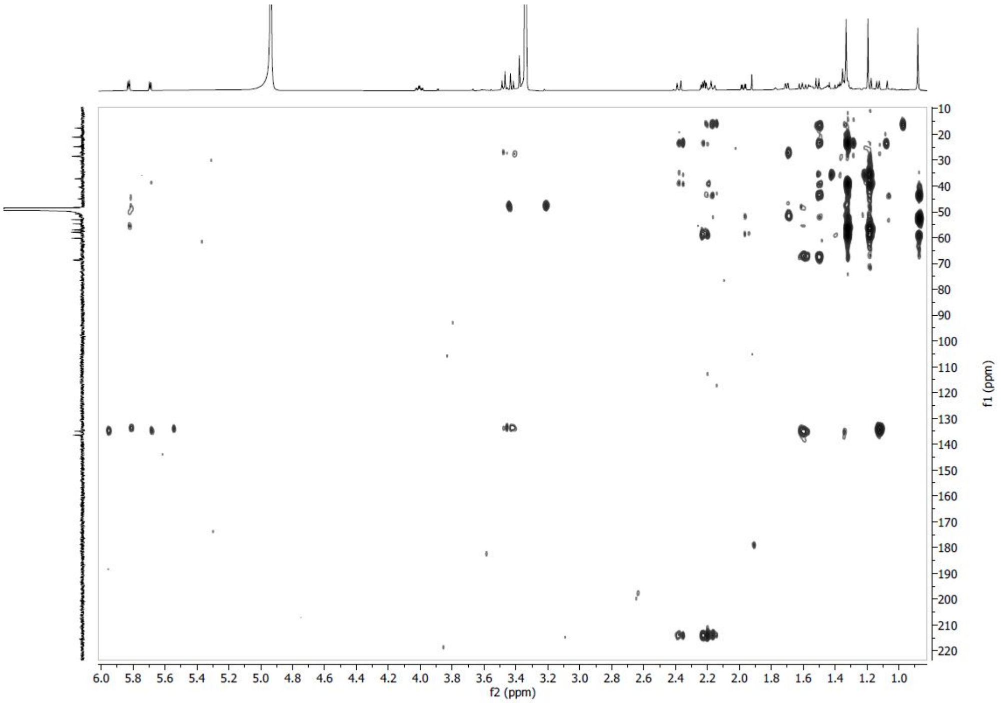

**Figure S 51.** $^{13}$C NMR spectrum of compound **9** ($CD_3OD$, 600 MHz)

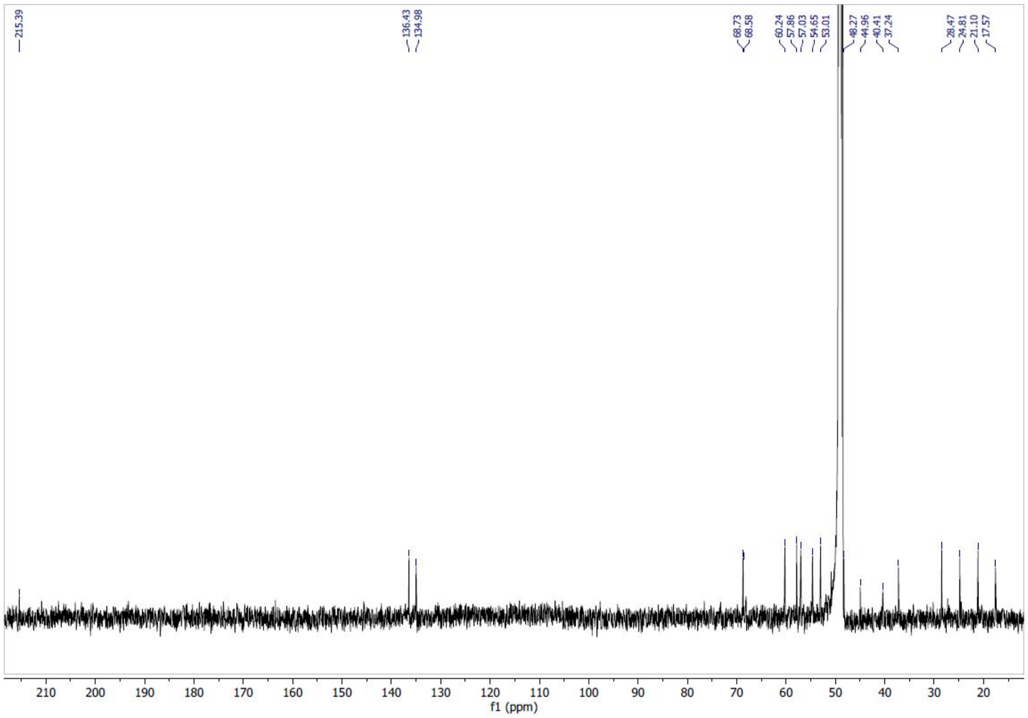


**Figure S 52.** HRESIMS of compound **9**

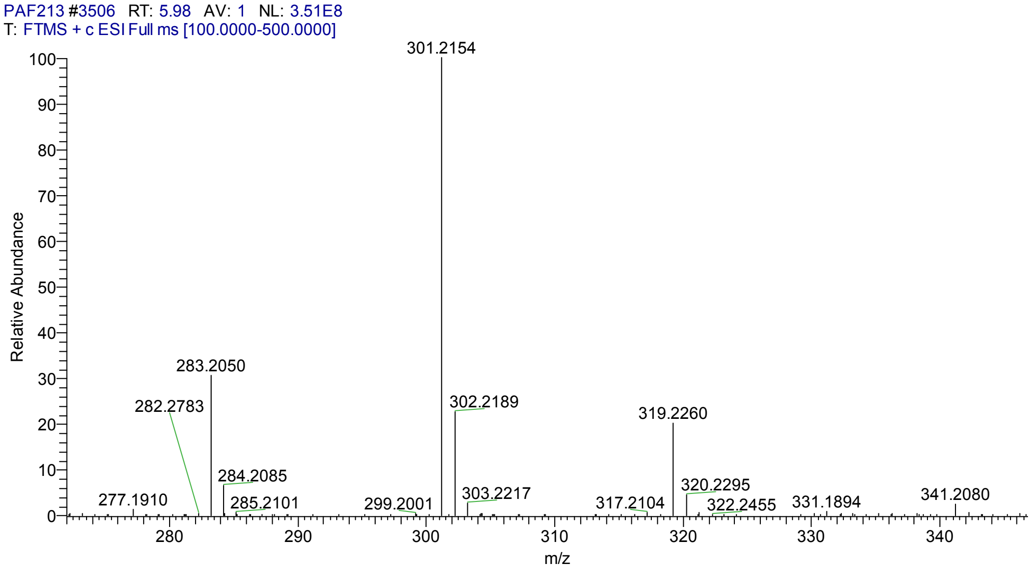

**Figure S 53.** $^{1}H$ NMR spectrum of compound **10** ($CD_3OD$, 600 MHz)

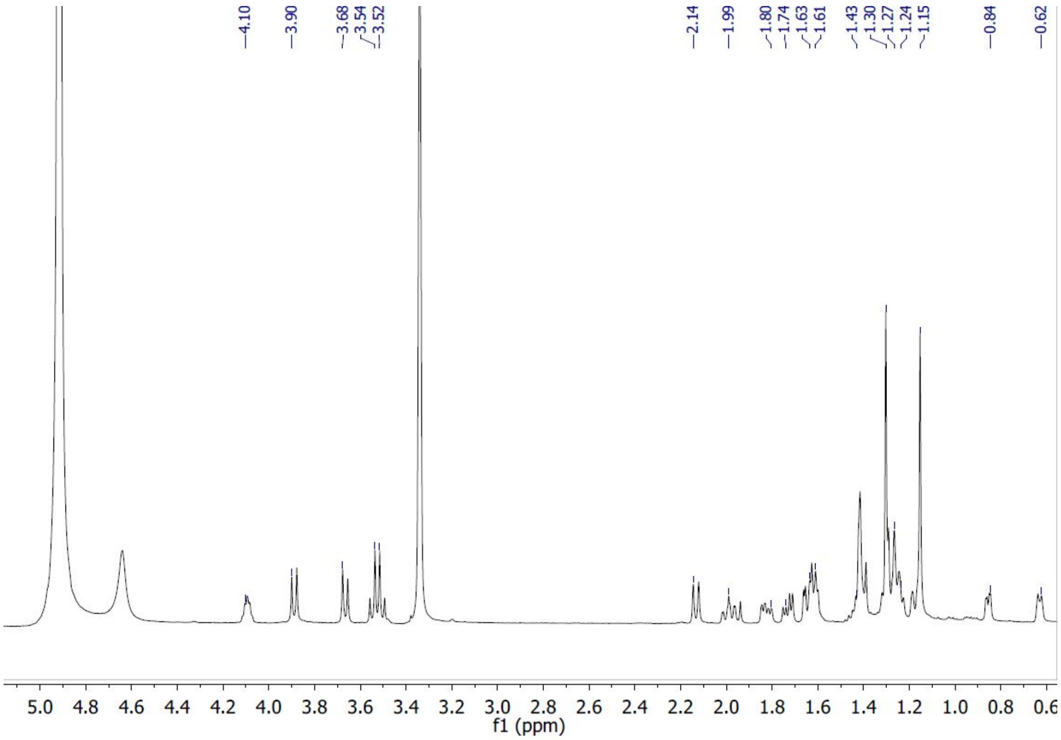


**Figure S 54.** COSY spectrum of compound **10** ($CD_3OD$, 600 MHz)

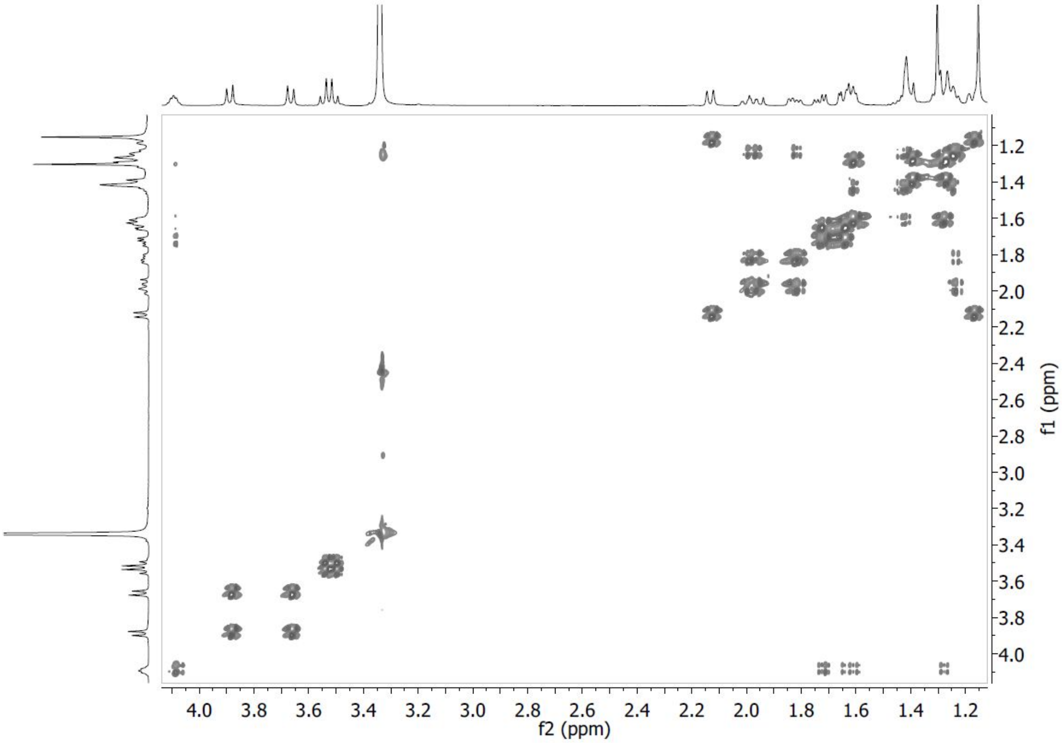

**Figure S 55.** HSQC spectrum of compound **10** ($CD_3OD$, 600 MHz)

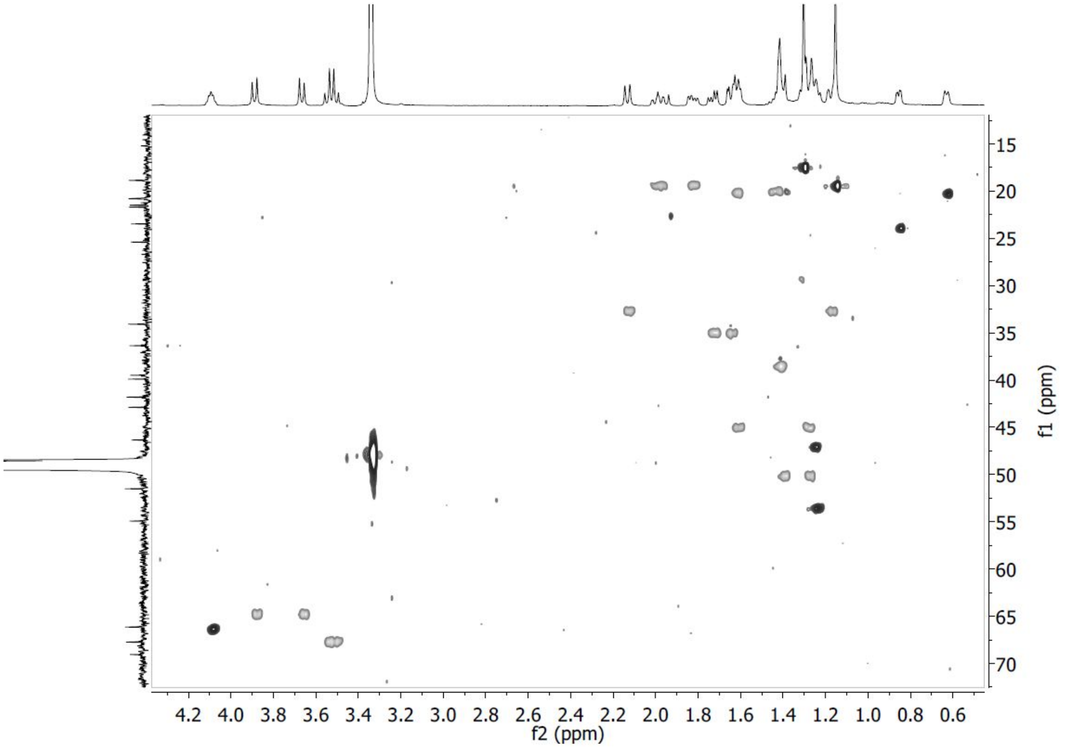

**Figure S 56.** HMBC spectrum of compound **10** ($CD_3OD$, 600 MHz)

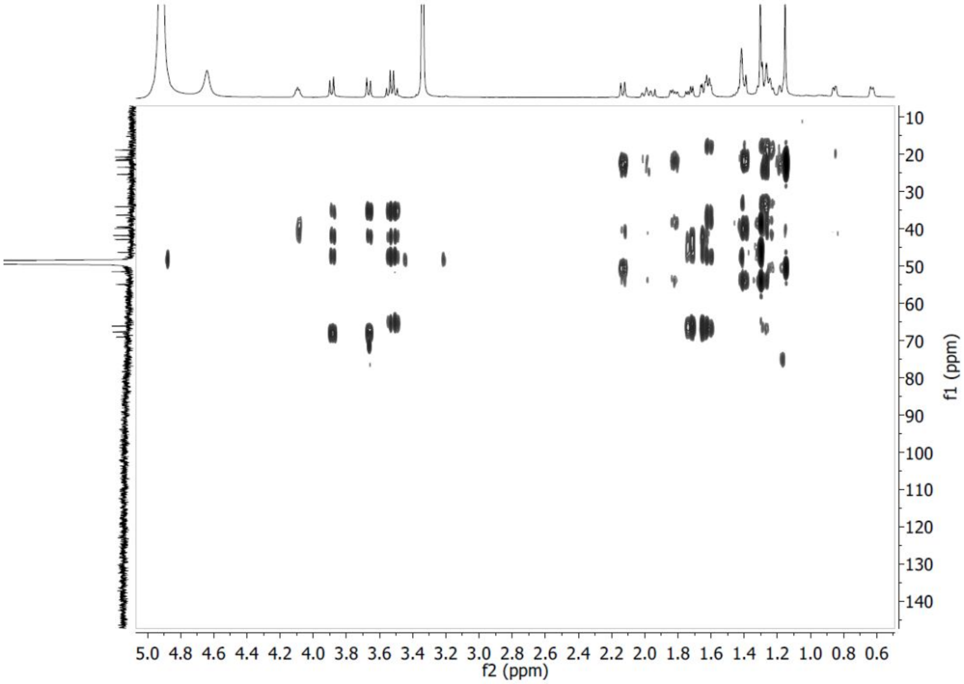

**Figure S 57.** $^{13}C$ NMR spectrum of compound **10** ($CD_3OD$, 600 MHz)

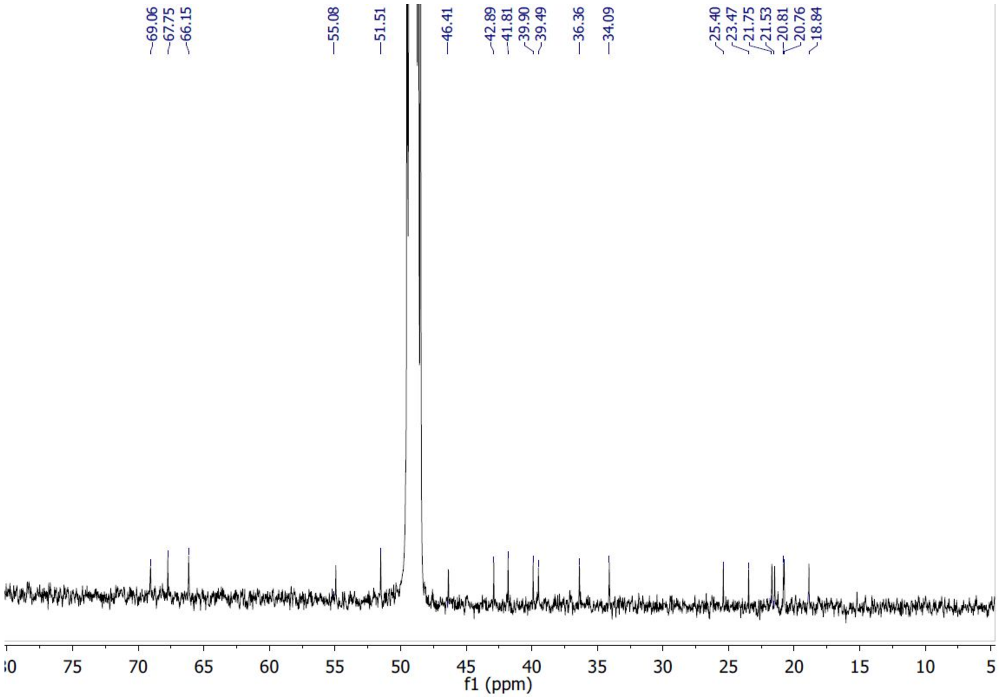


**Figure S 58.** HRESIMS of compound **10**

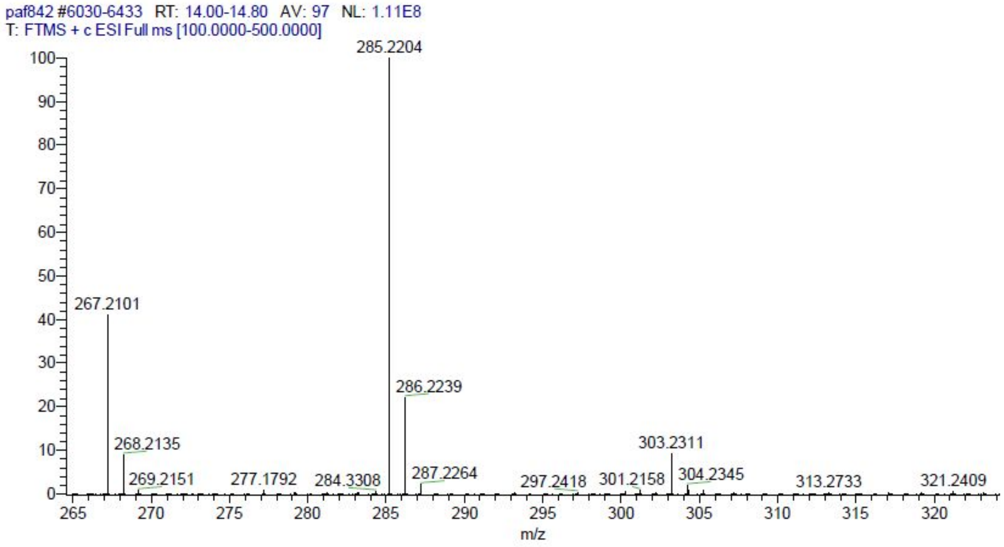

**Figure S 59.** $^{1}H$ NMR spectrum of compound **11** ($CD_3OD$, 600 MHz)

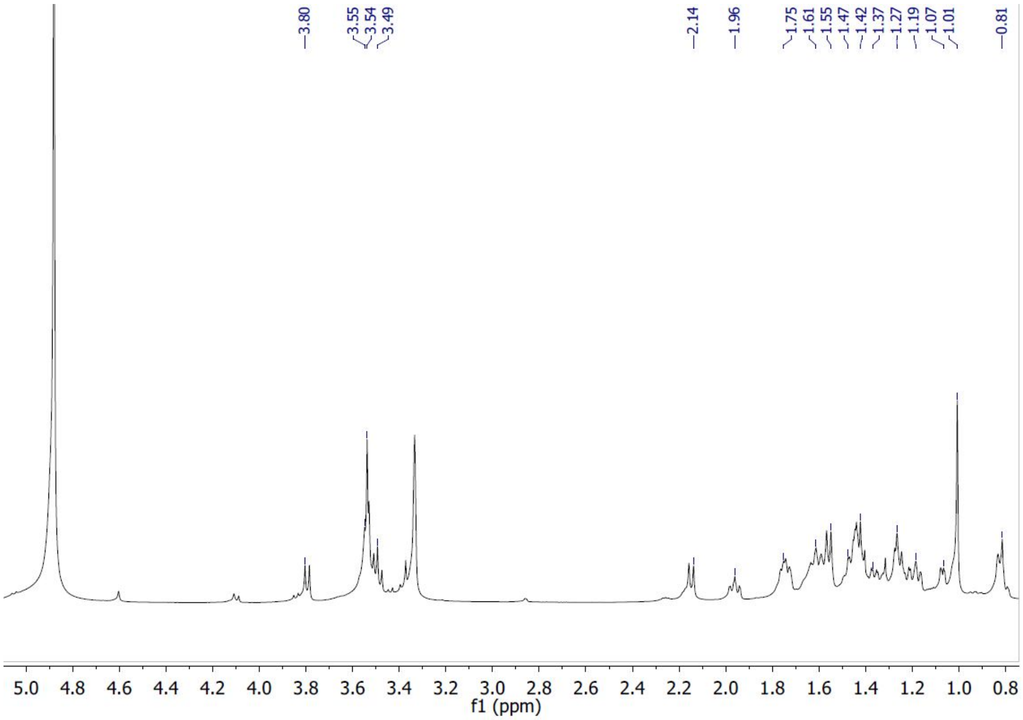


**Figure S 60.** COSY spectrum of compound **11** ($CD_3OD$, 600 MHz)

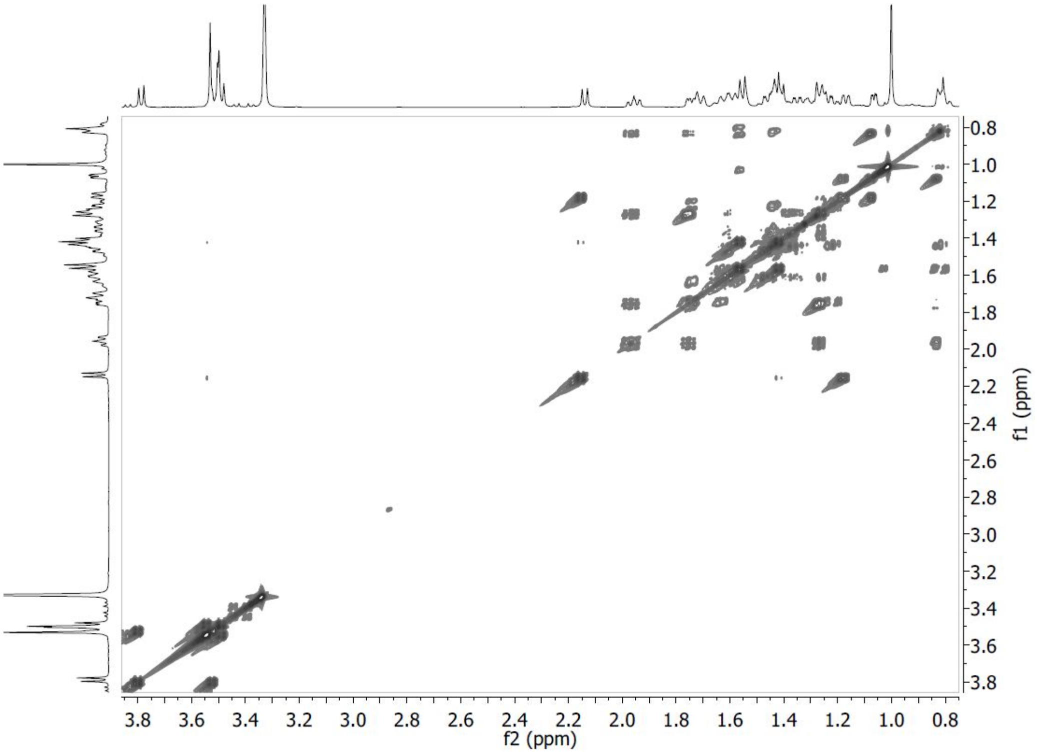

**Figure S 61.** HSQC spectrum of compound **11** ($CD_3OD$, 600 MHz)

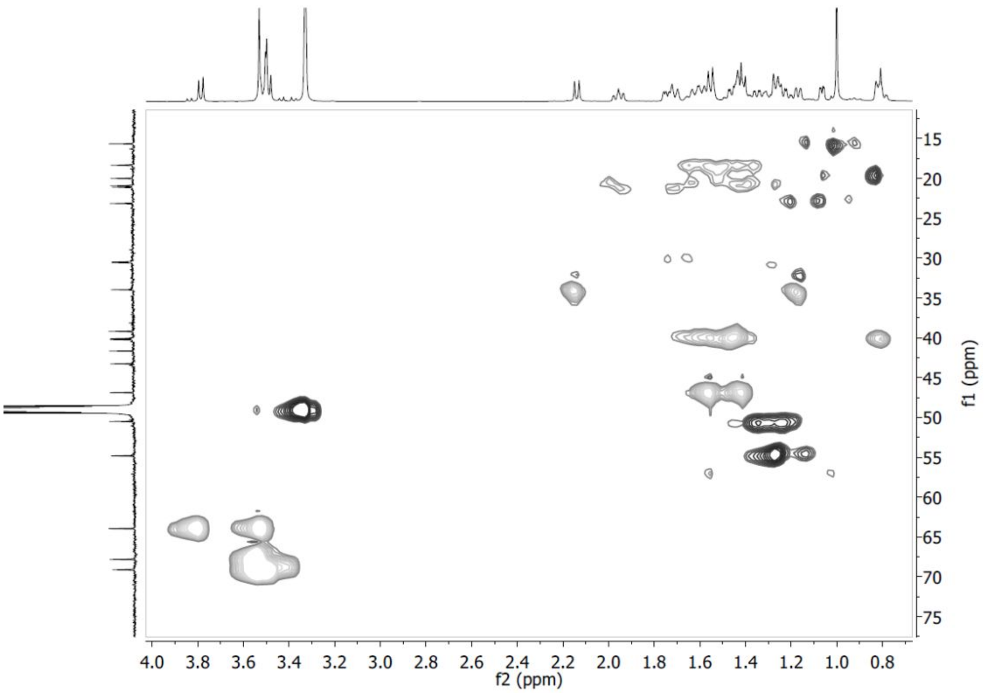


**Figure S 62.** HMBC spectrum of compound **11** ($CD_3OD$, 600 MHz)

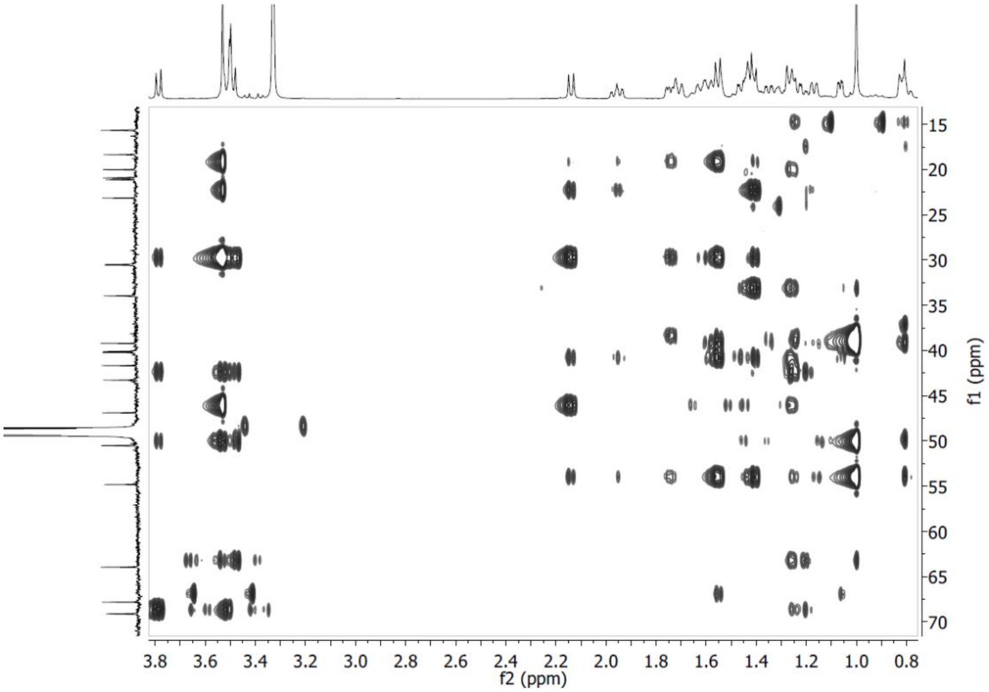

**Figure S 63.** $^{13}$C NMR spectrum of compound **11** ($CD_3OD$, 600 MHz)

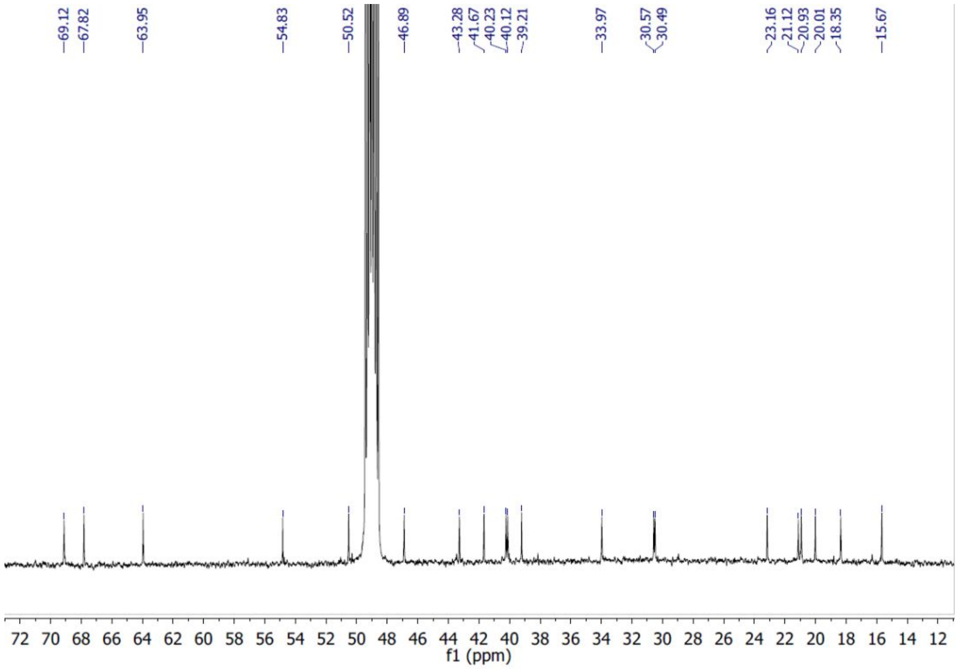


**Figure S 64.** HRESIMS of compound **11**

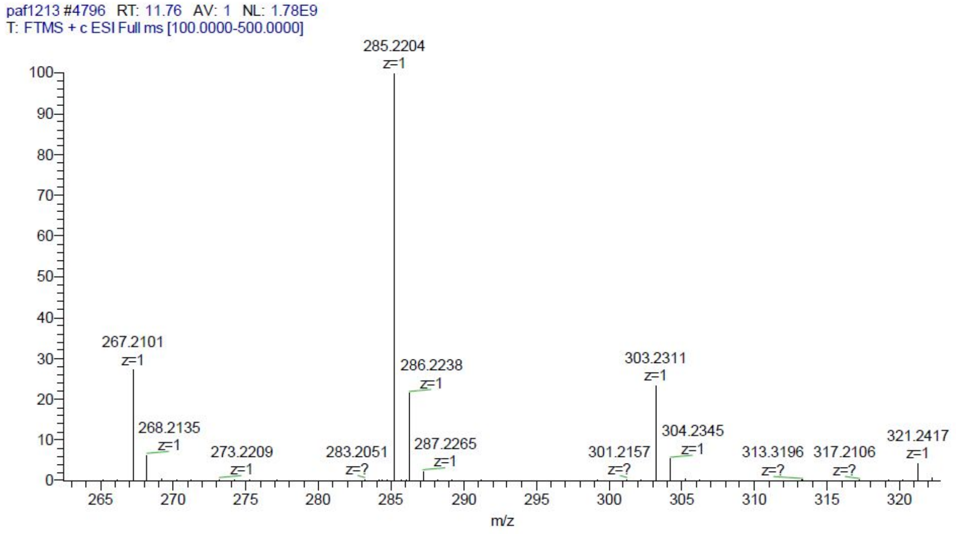

**Figure S 65.** $^{1}$H NMR spectrum of compound **12** ($CD_3OD$, 600 MHz)

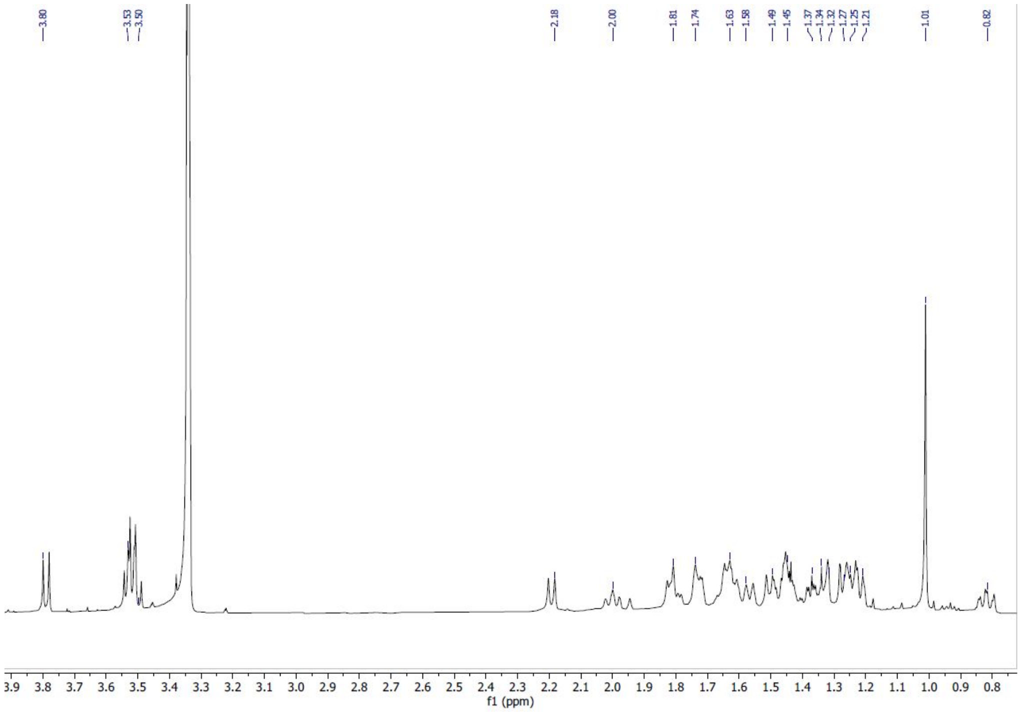

**Figure S 66.** COSY spectrum of compound **12** ($CD_3OD$, 600 MHz)

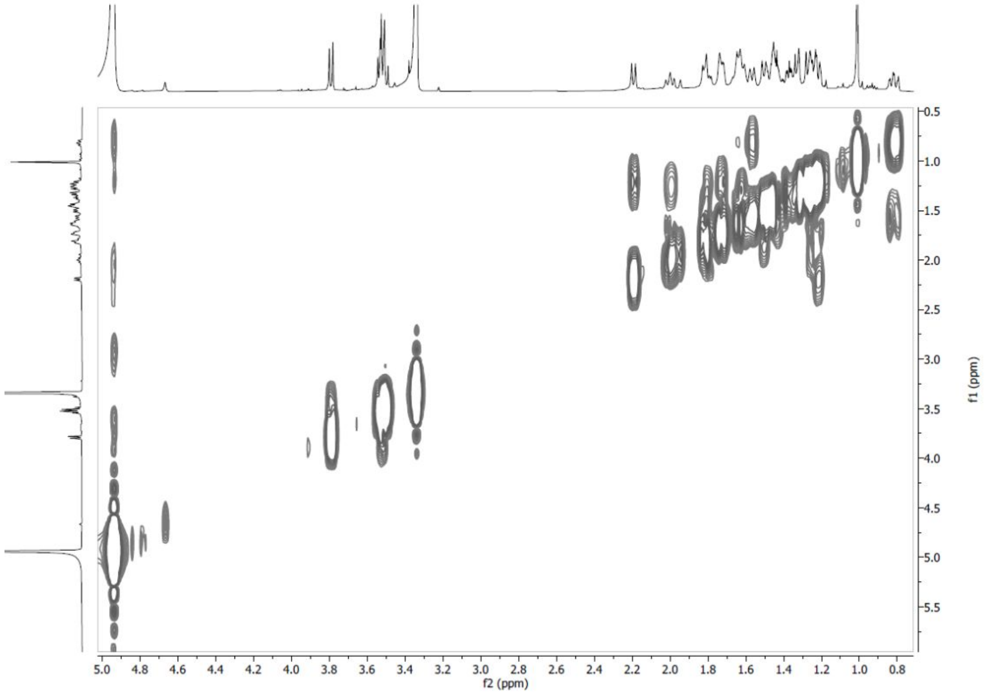

**Figure S 67.** HSQC spectrum of compound **12** ($CD_3OD$, 600 MHz)

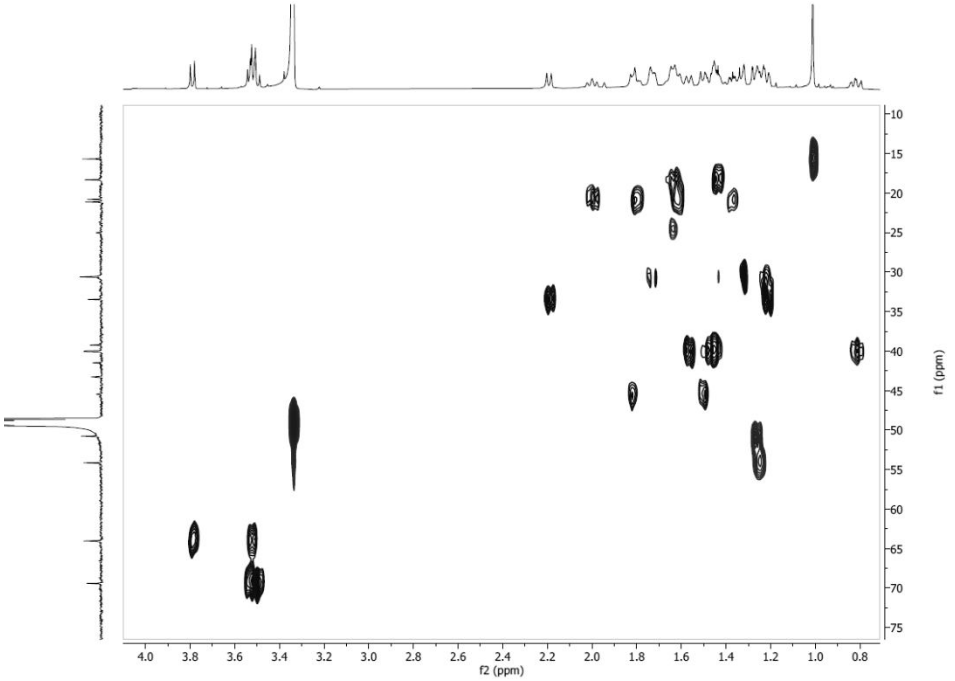


**Figure S 68.** HMBC spectrum of compound **12** ($CD_3OD$, 600 MHz)

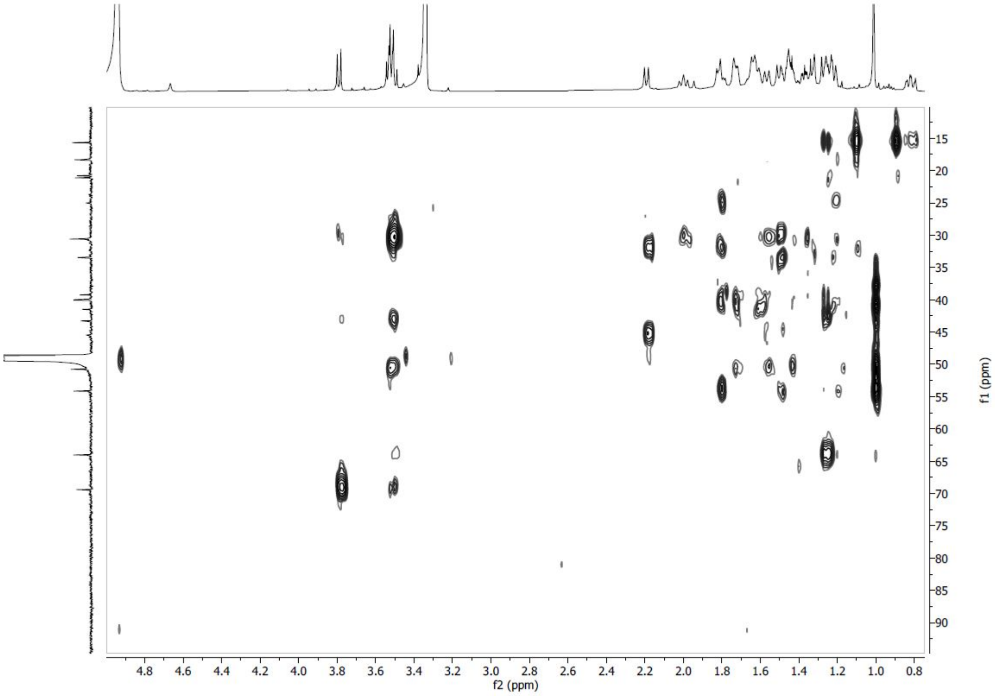

**Figure S 69.** $^{13}C$ NMR spectrum of compound **12** ($CD_3OD$, 600 MHz)

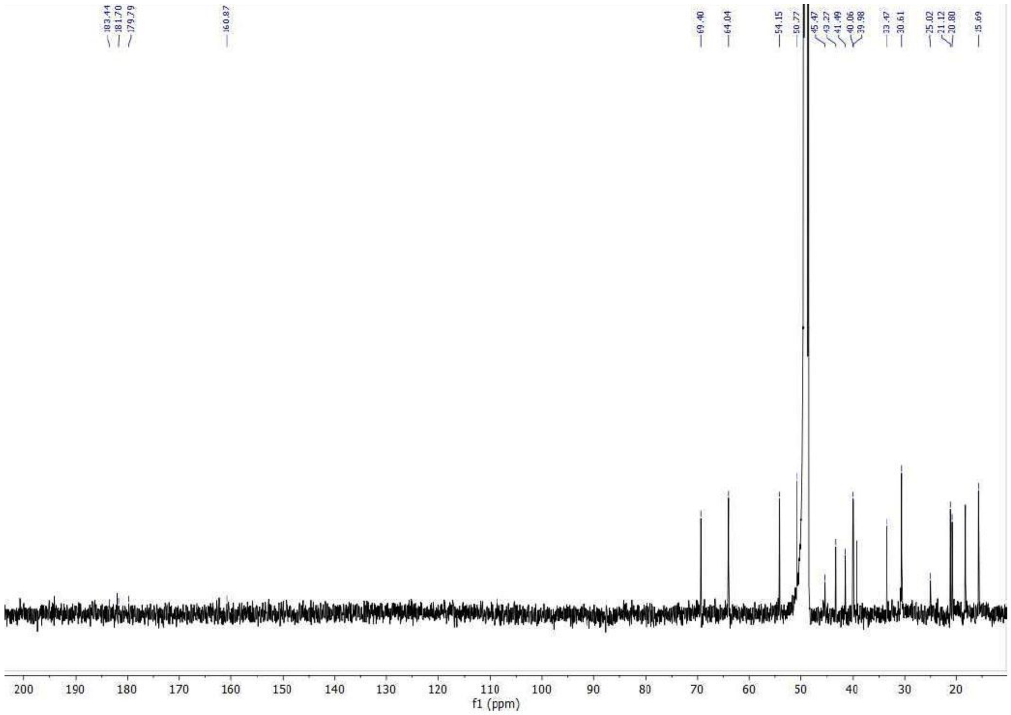


**Figure S 70.** HRESIMS of compound **12**

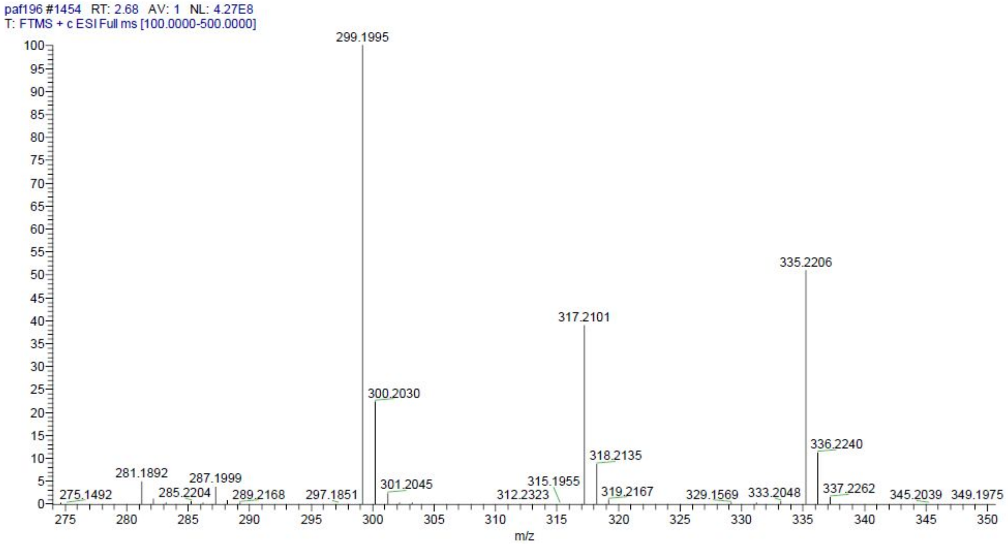

**Figure S 71.** $^{1}H$ NMR spectrum of compound **13** ($CD_3OD$, 600 MHz)

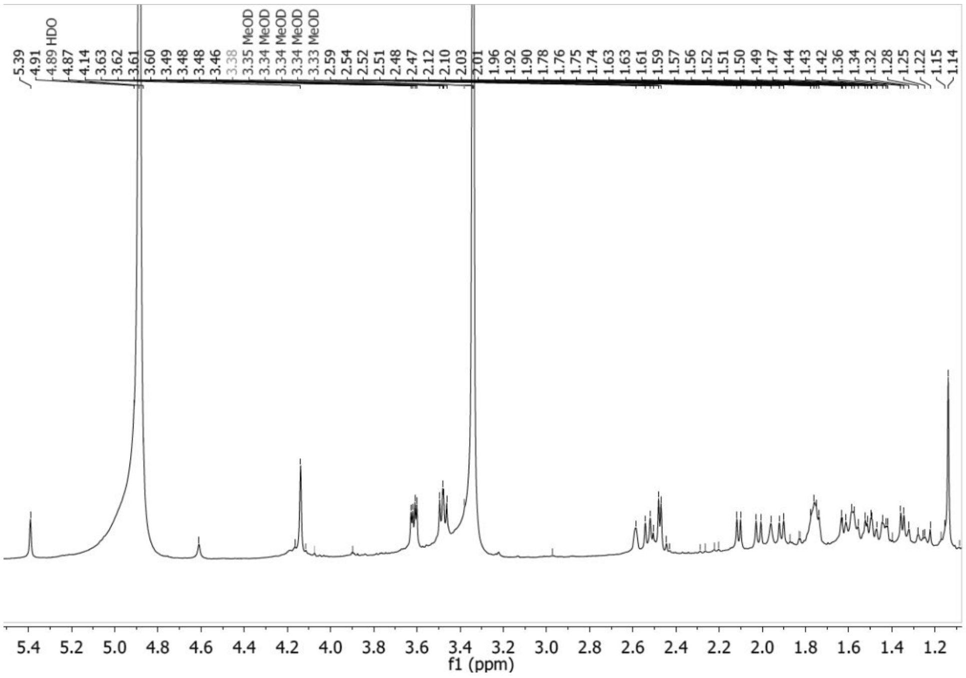

**Figure S 72.** COSY spectrum of compound **13** ($CD_3OD$, 600 MHz)

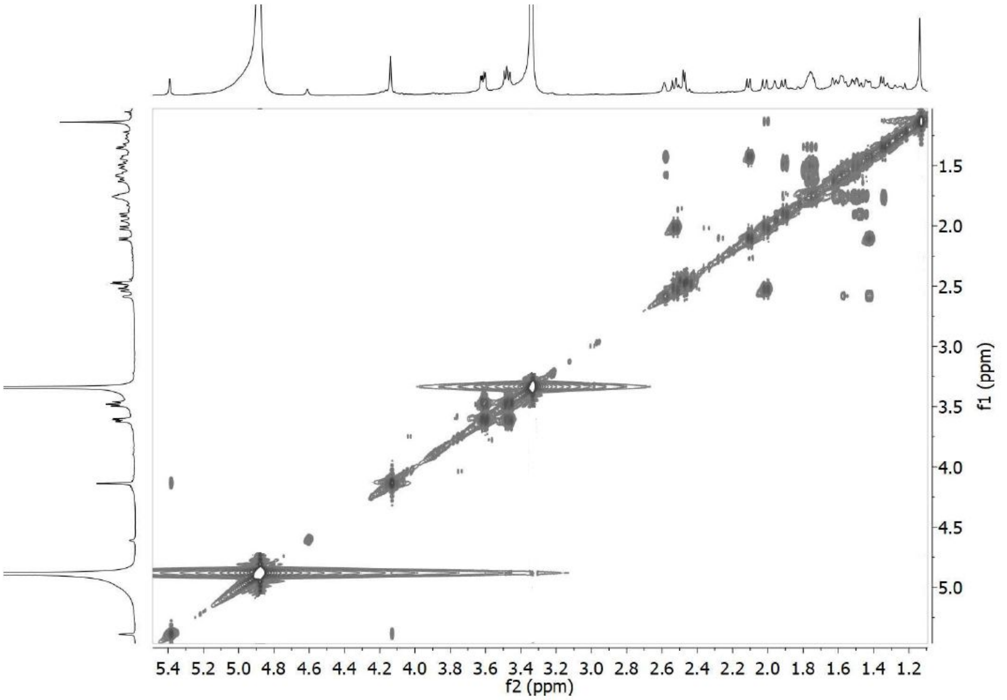

**Figure S 73.** HSQC spectrum of compound **13** ($CD_3OD$, 600 MHz)

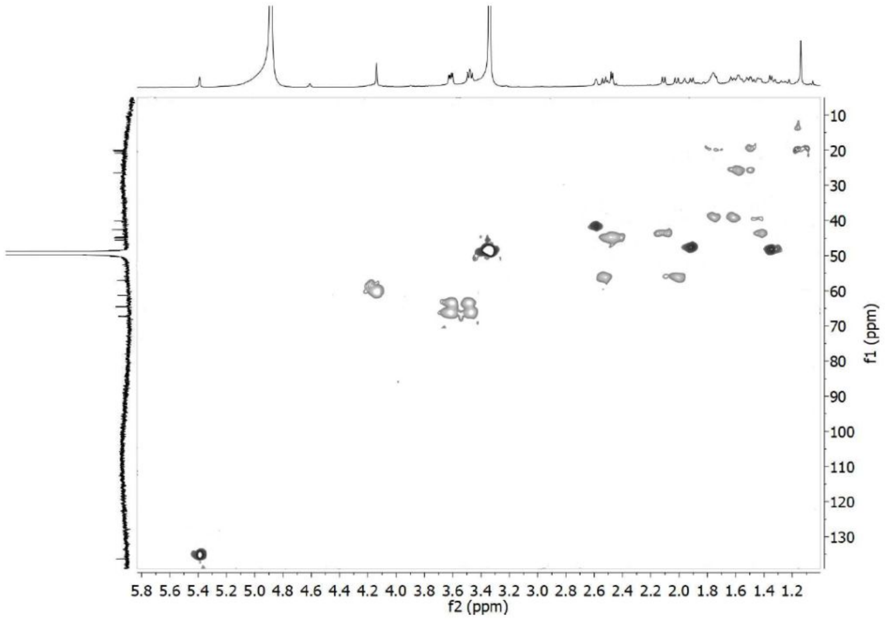


**Figure S 74.** HMBC spectrum of compound **13** ($CD_3OD$, 600 MHz)

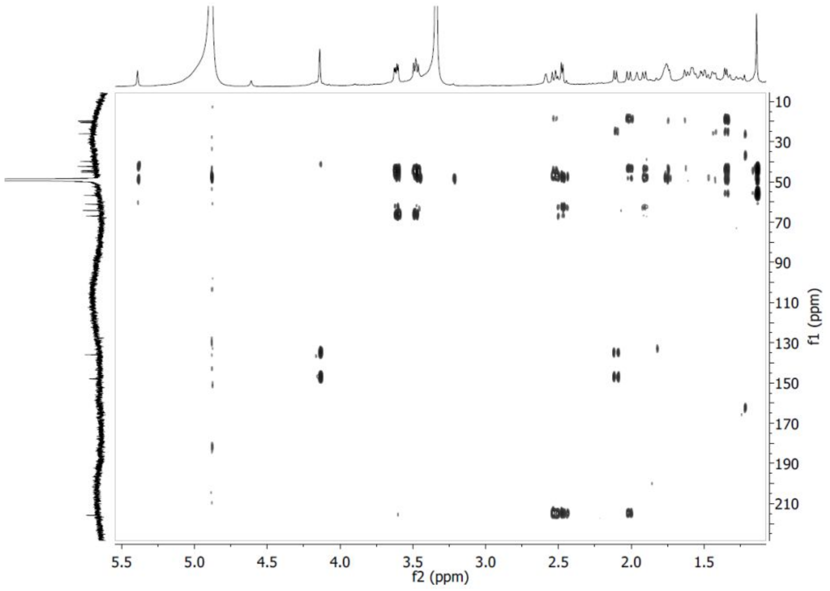

**Figure S 75.** $^{13}C$ NMR spectrum of compound **13** ($CD_3OD$, 600 MHz)

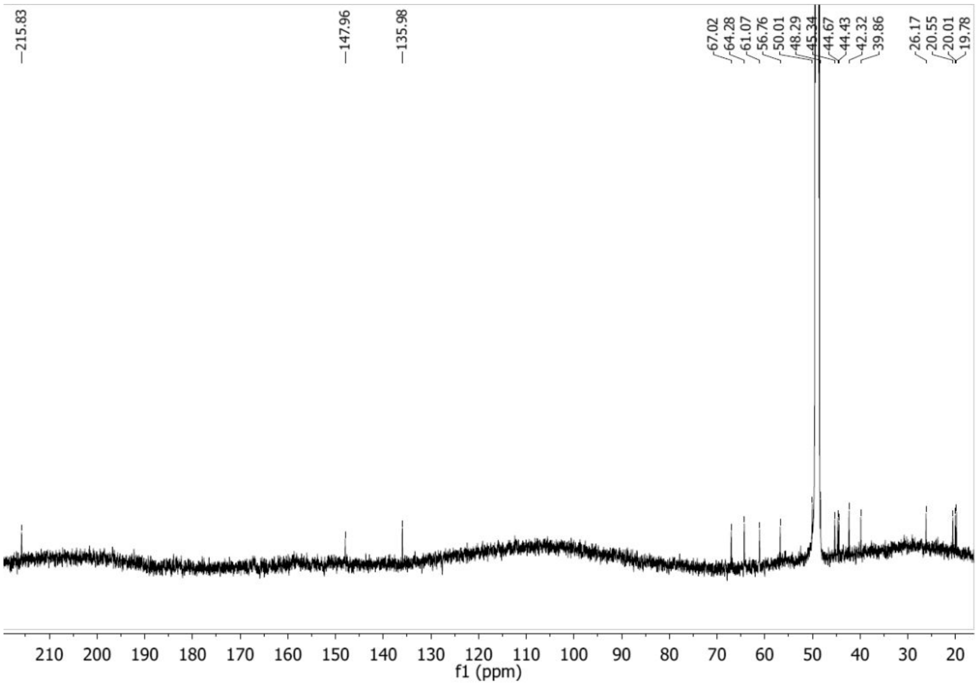


**Figure S 76.** HRESIMS of compound **13**

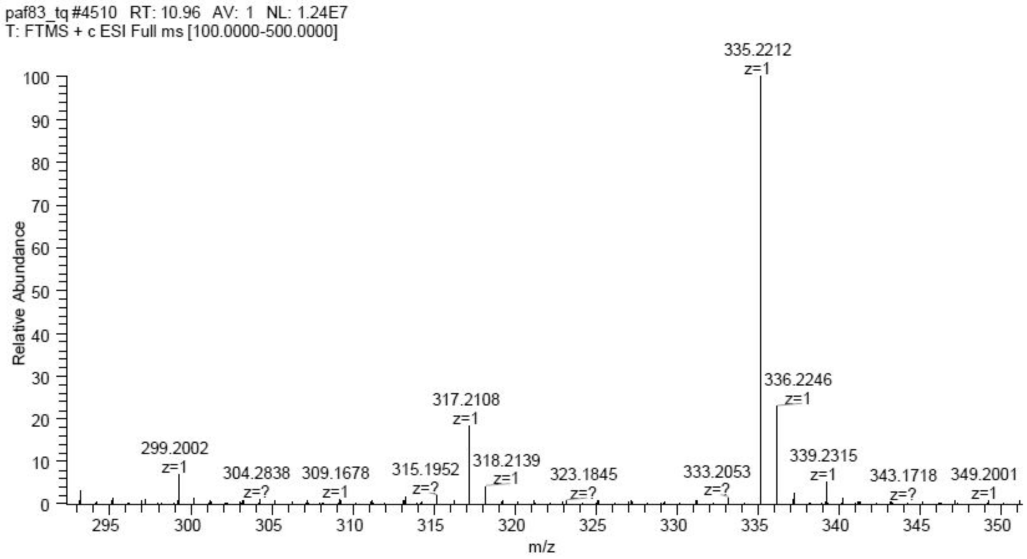

**Figure S 77.** $^{1}H$ NMR spectrum of compound **14** ($CD_3OD$, 600 MHz)

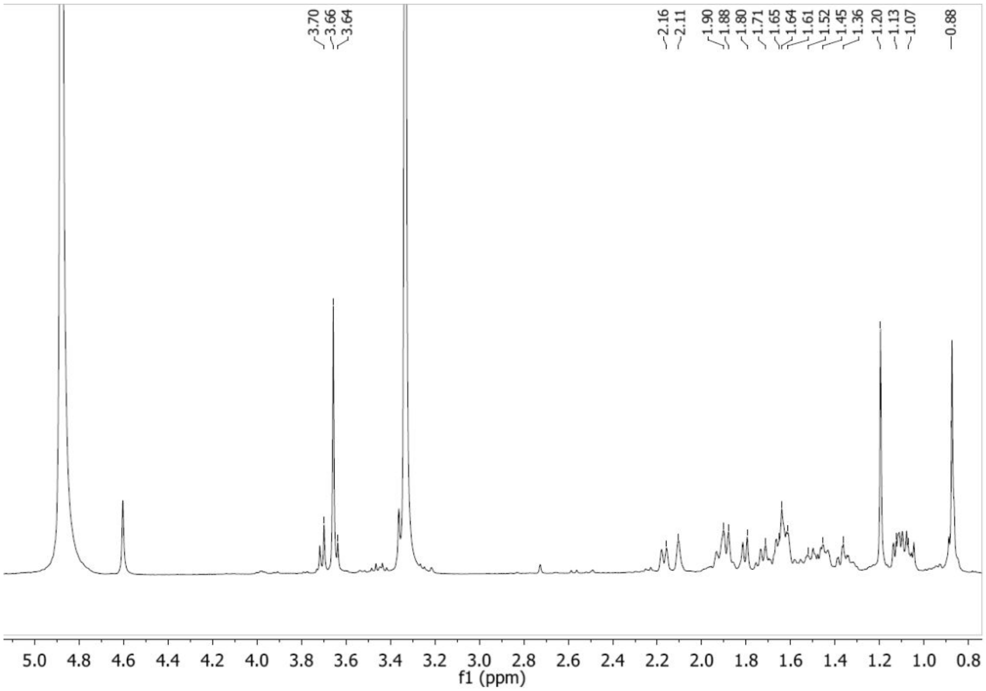


**Figure S 78.** COSY spectrum of compound **14** ($CD_3OD$, 600 MHz)

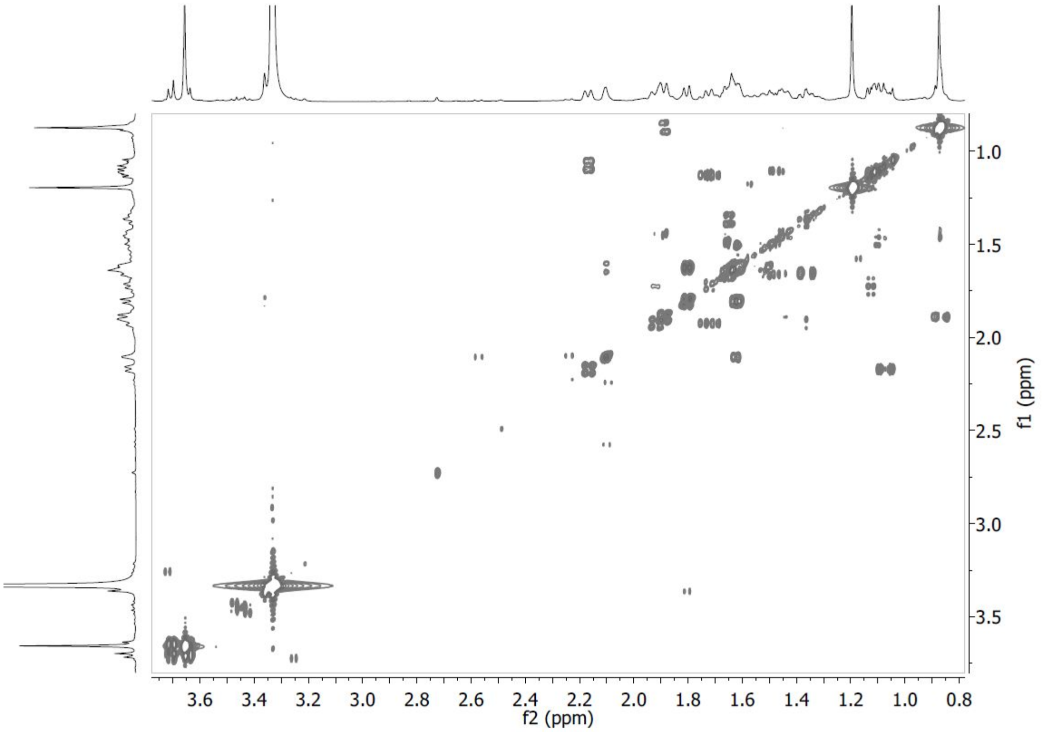

**Figure S 79.** HSQC spectrum of compound **14** ($CD_3OD$, 600 MHz)

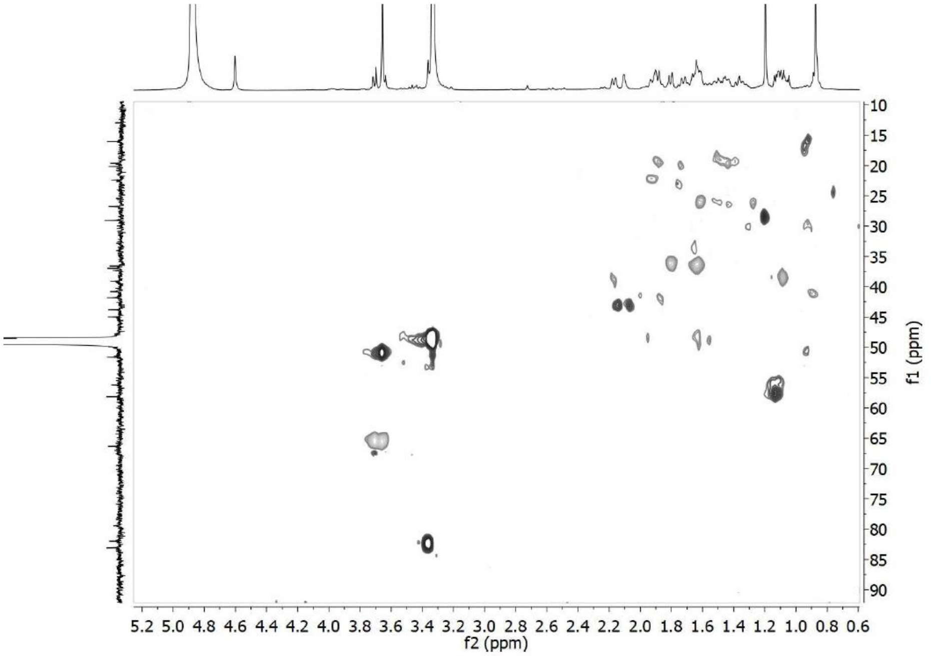


**Figure S 80.** HMBC spectrum of compound **14** ($CD_3OD$, 600 MHz)

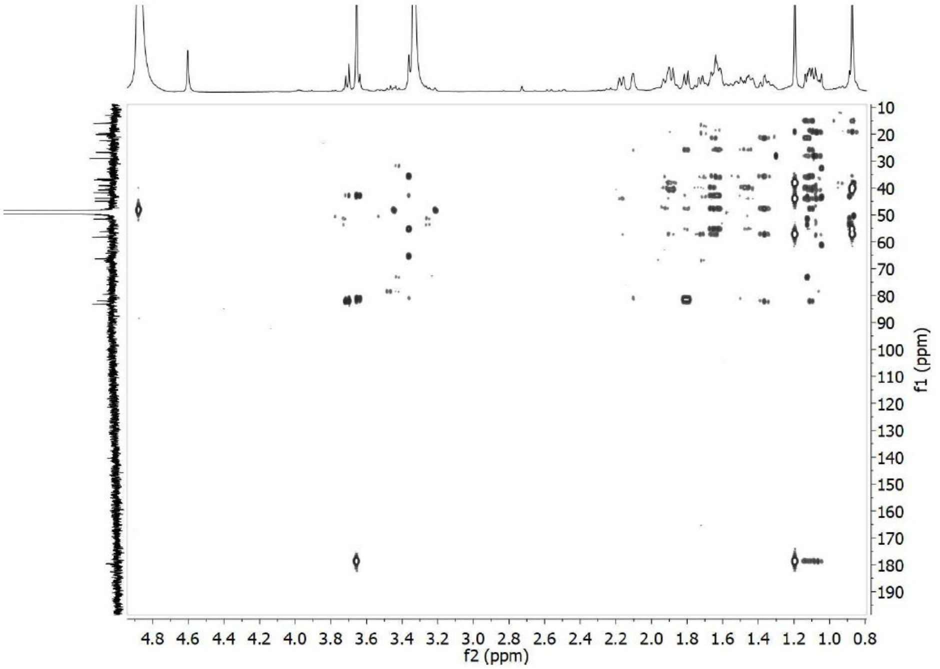

**Figure S 81.** $^{13}C$ NMR spectrum of compound **14** ($CD_3OD$, 600 MHz)

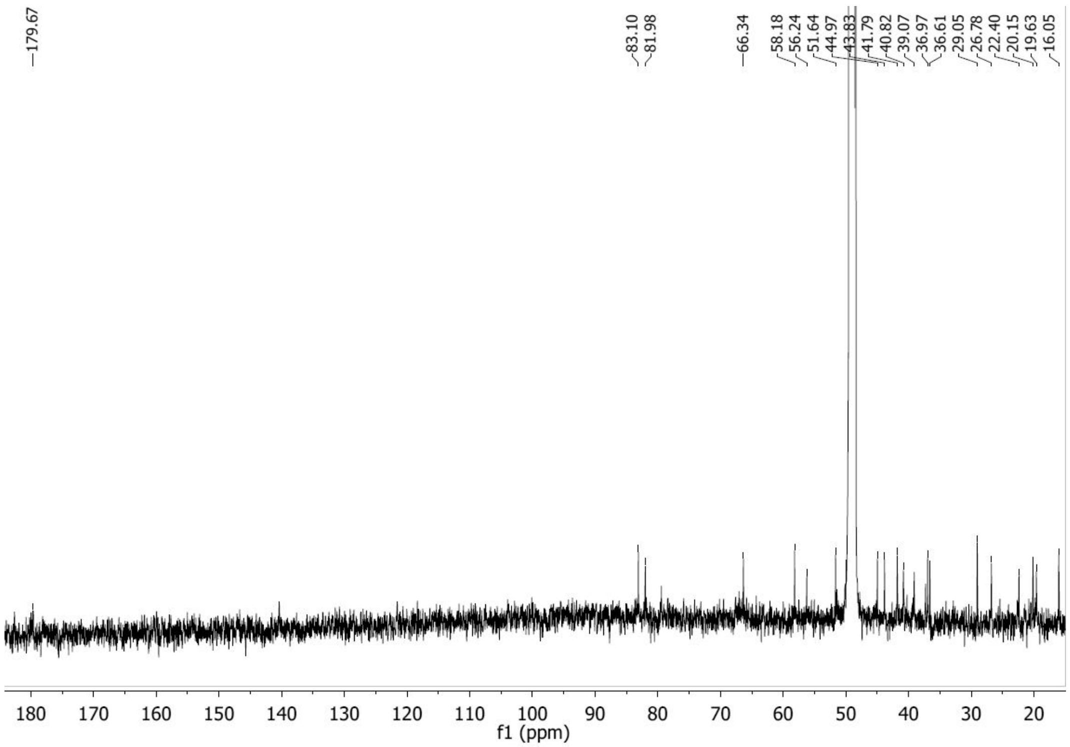


**Figure S 82.** HRESIMS of compound **14**

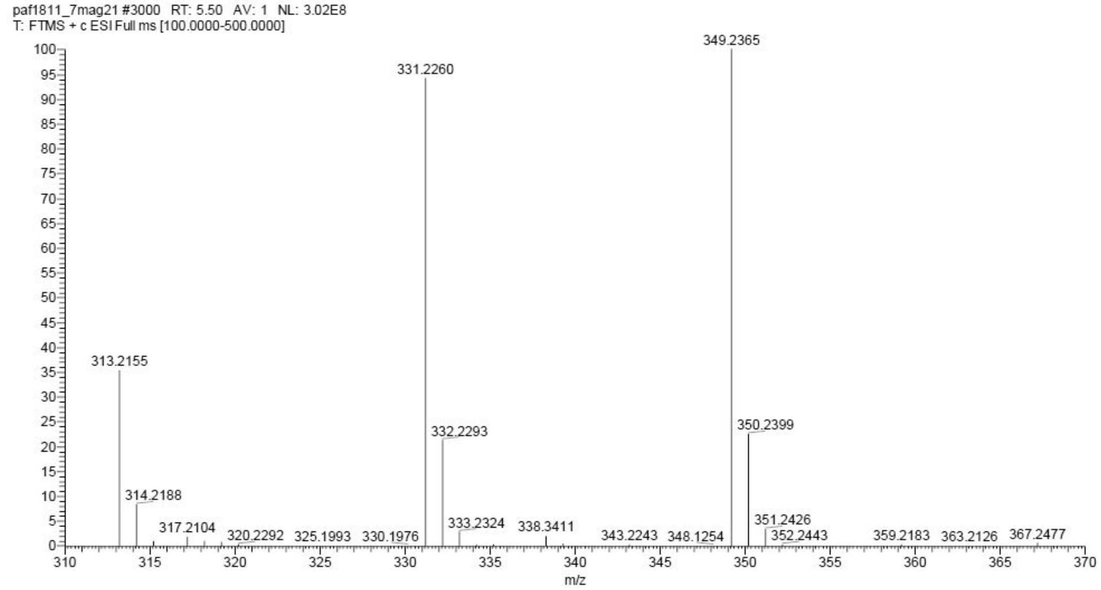

**Figure S 83.** $^{1}$H NMR spectrum of compound **15** ($CD_3OD$, 600 MHz)

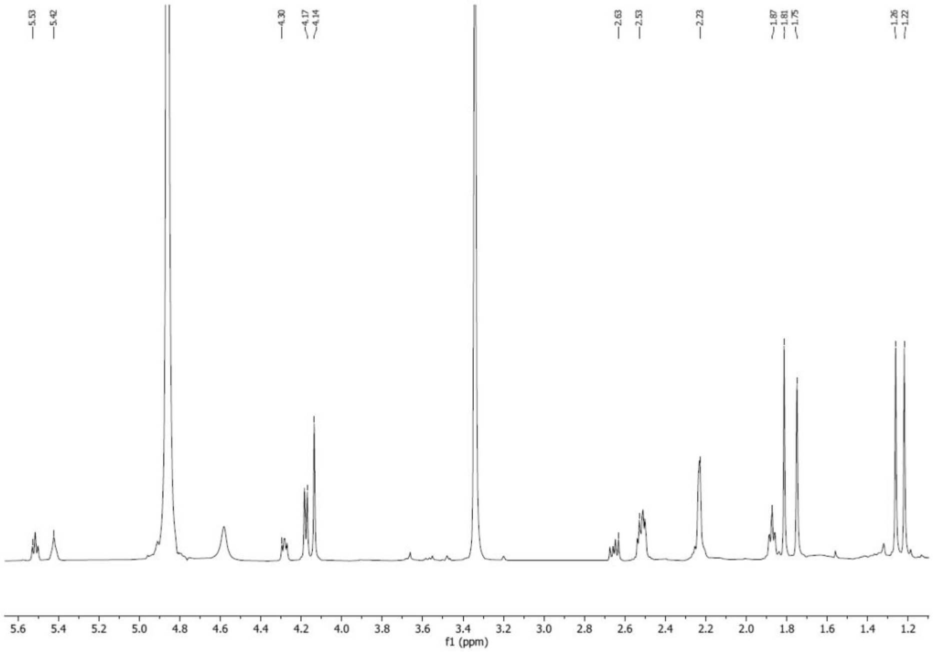


**Figure S 84.** COSY spectrum of compound **15** ($CD_3OD$, 600 MHz)

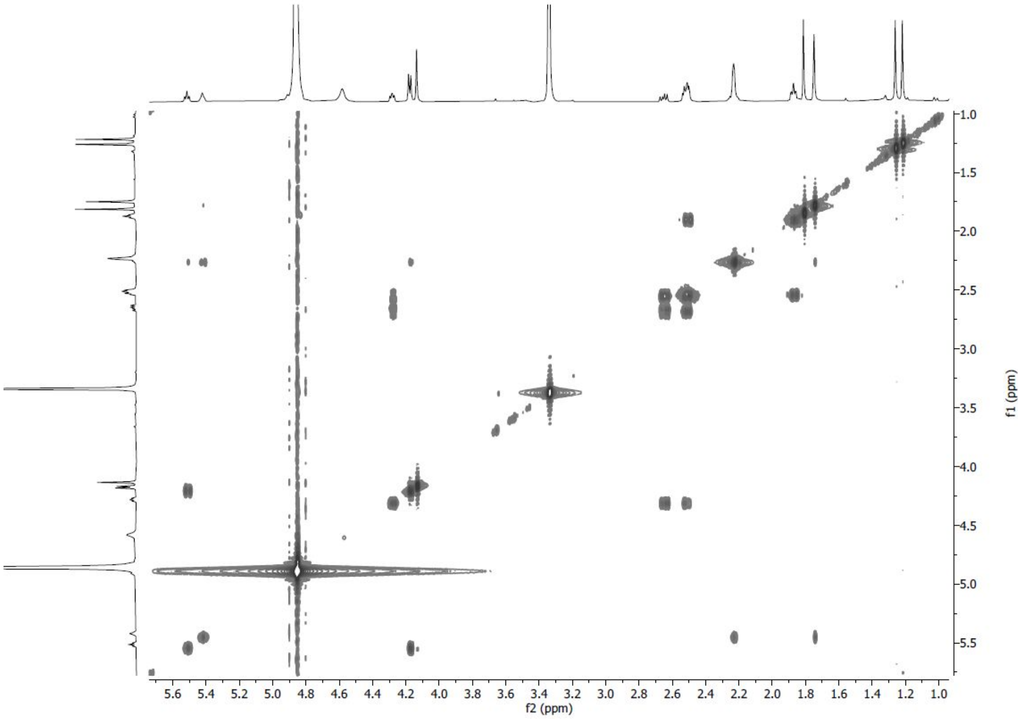

**Figure S 85.** HSQC spectrum of compound **15** ($CD_3OD$, 600 MHz)

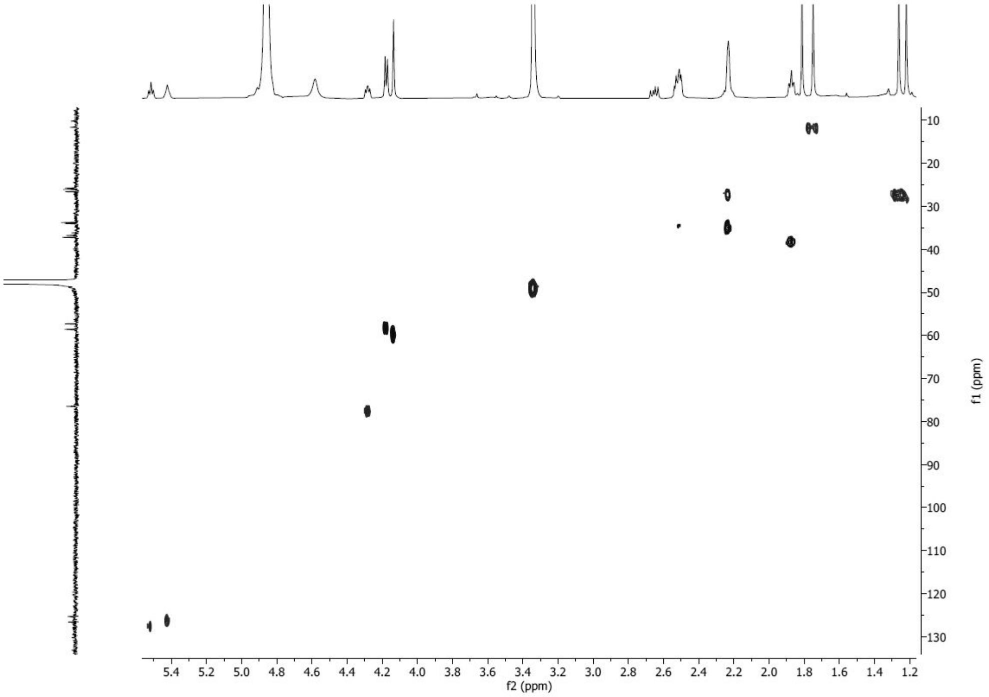

**Figure S 86.** HMBC spectrum of compound **15** ($CD_3OD$, 600 MHz)

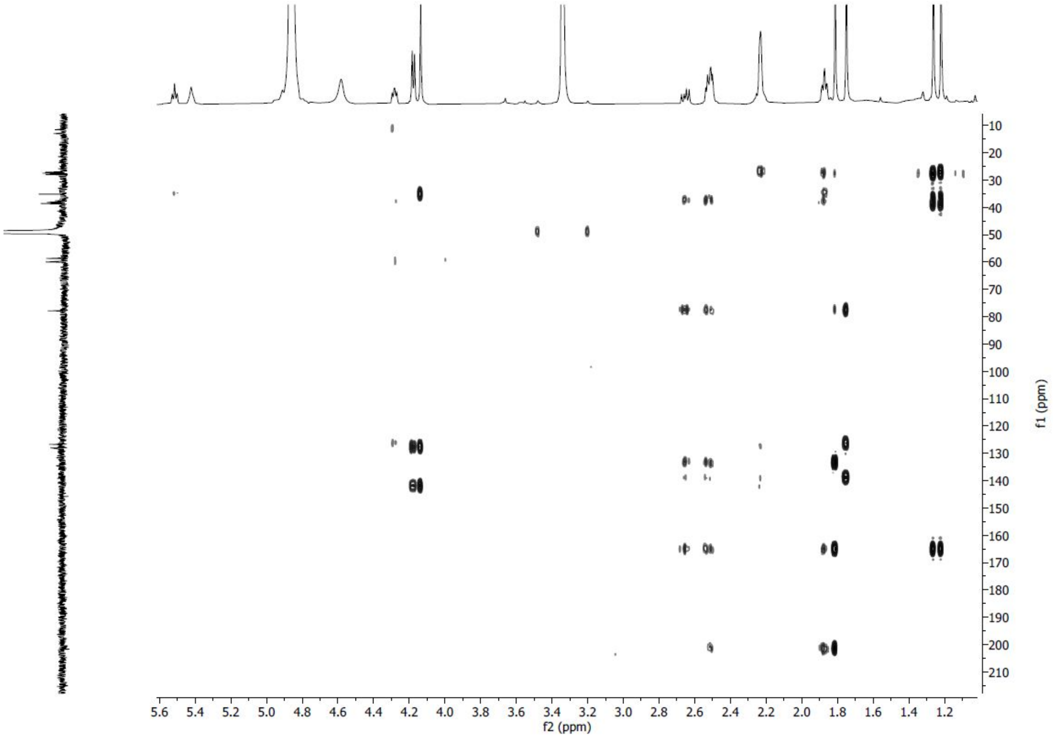

**Figure S 87.** $^{13}C$ NMR spectrum of compound **15** ($CD_3OD$, 600 MHz)

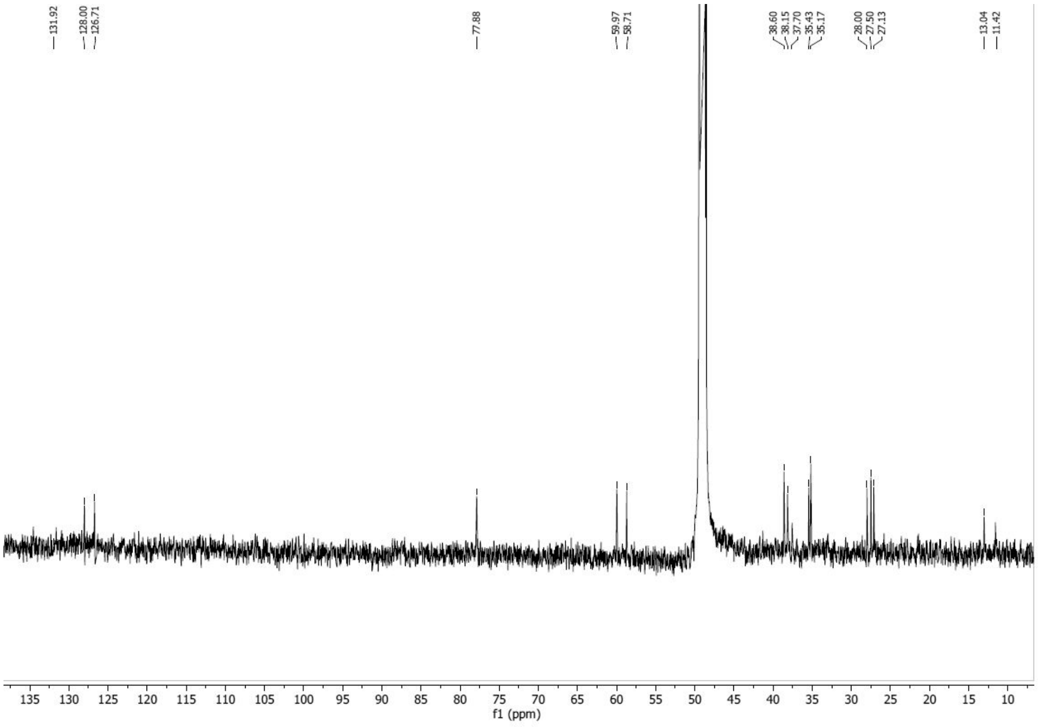


**Figure S 88.** HRESIMS of compound **15**

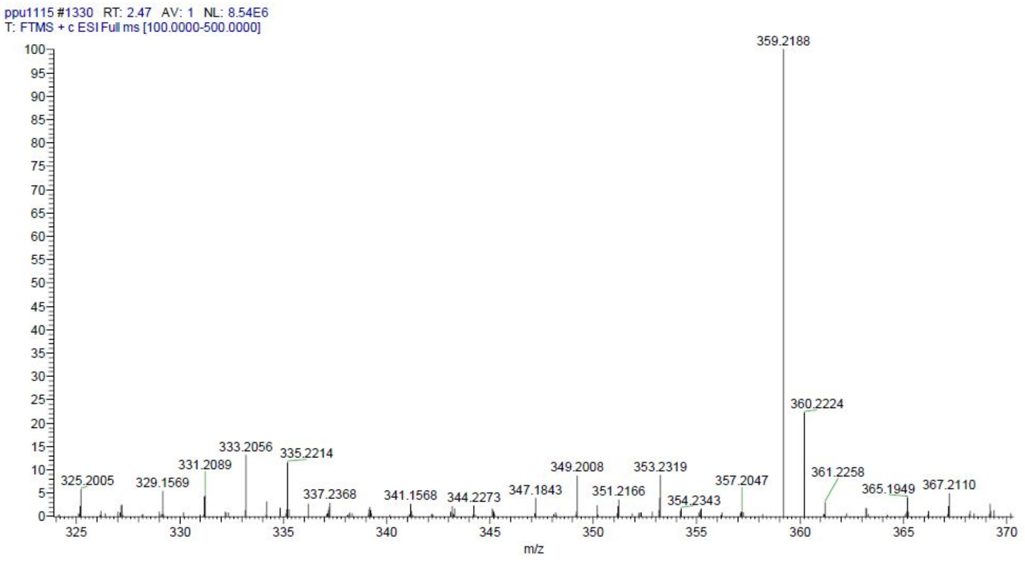

**Figure S 89.** $^{1}$H NMR spectrum of compound **16** ($CD_3OD$, 600 MHz)

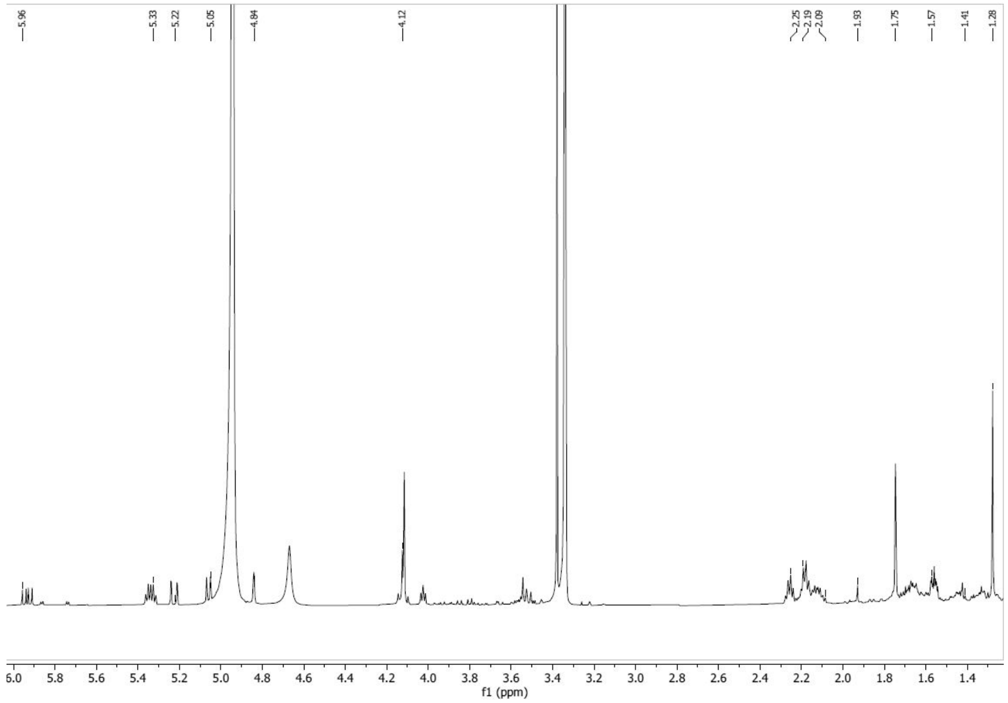

**Figure S 90.** COSY spectrum of compound **16** ($CD_3OD$, 600 MHz)

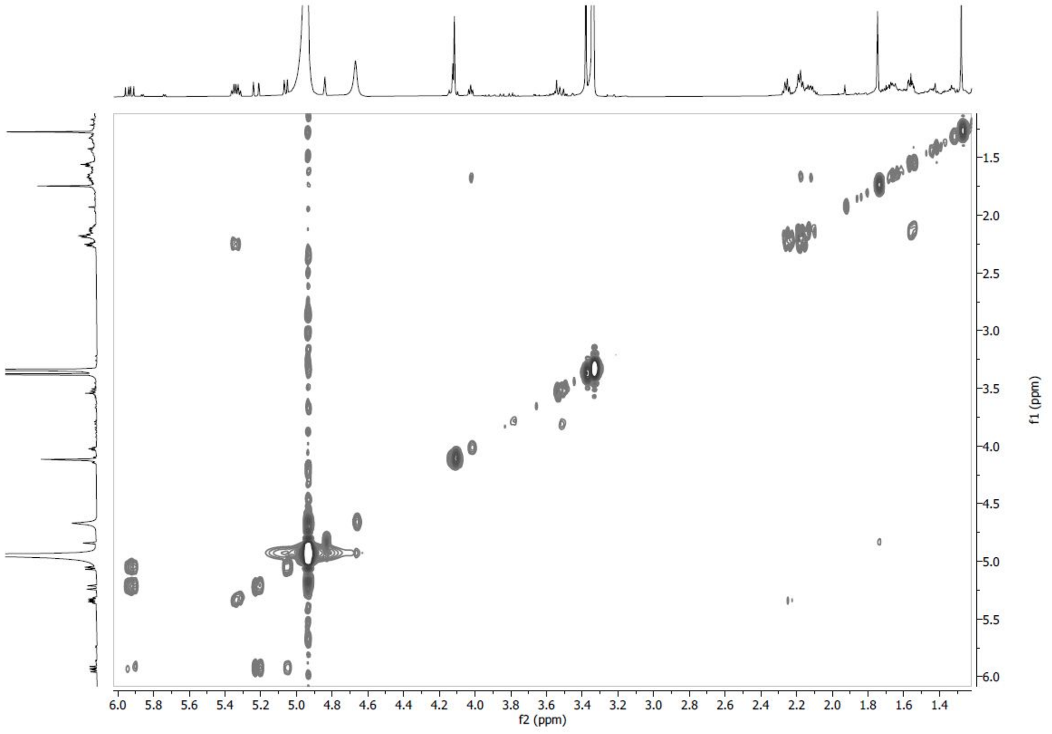

**Figure S 91.** HSQC spectrum of compound **16** ($CD_3OD$, 600 MHz)

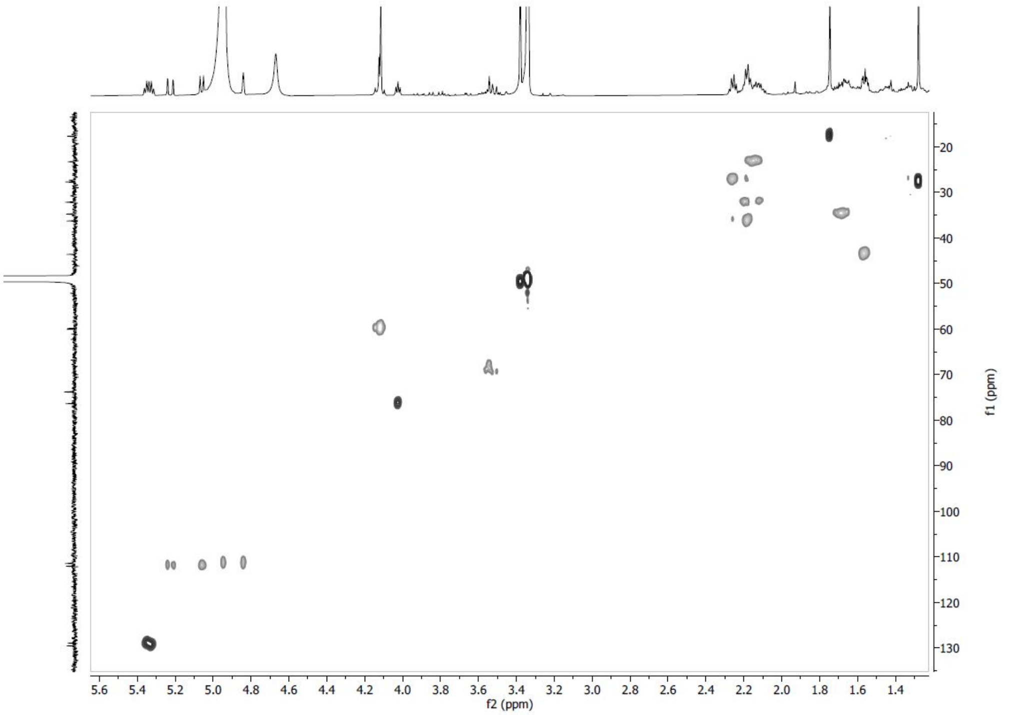


**Figure S 92.** HMBC spectrum of compound **16** ($CD_3OD$, 600 MHz)

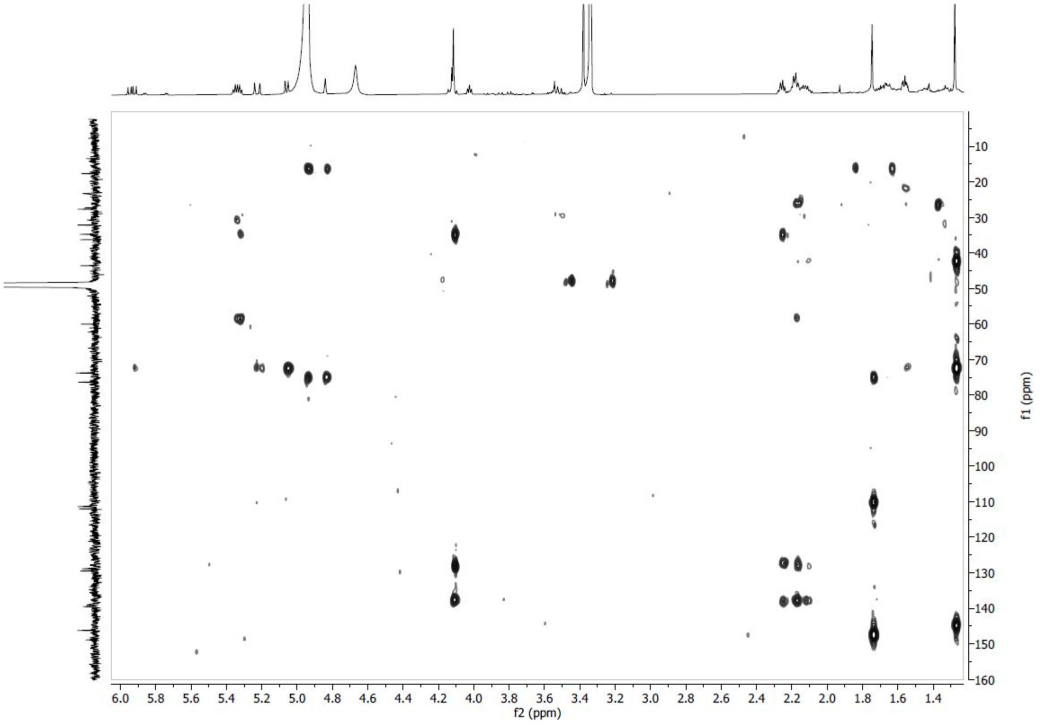

**Figure S 93.** $^{13}C$ NMR spectrum of compound **16** ($CD_3OD$, 600 MHz)

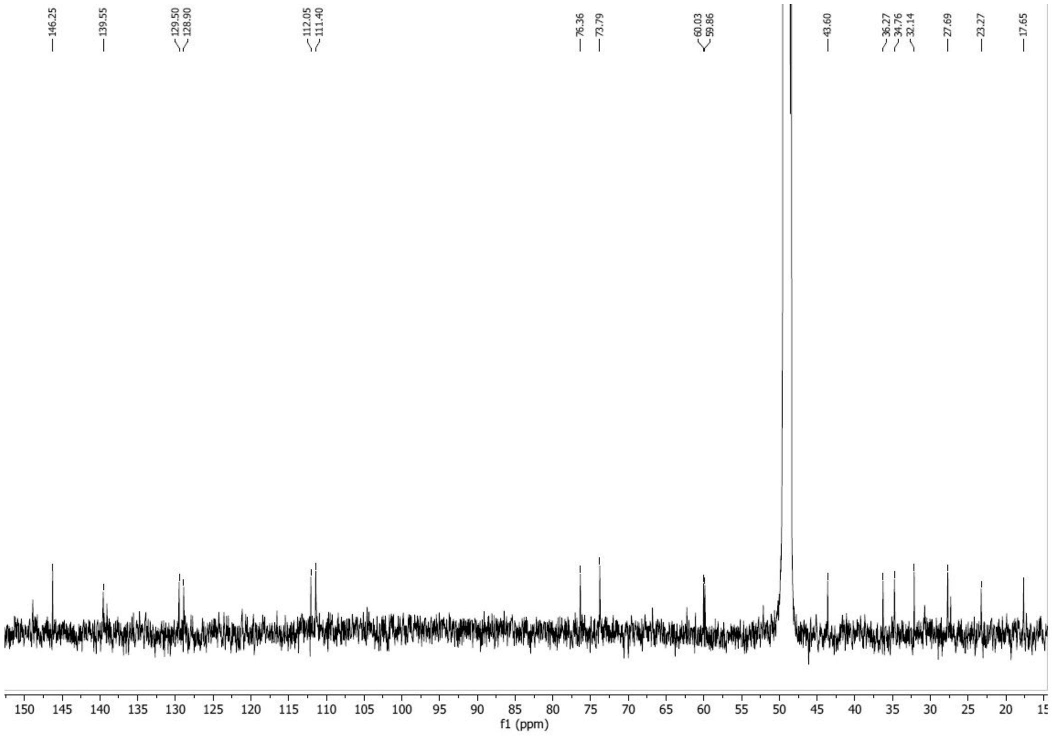

**Figure S 94.** HRESIMS of compound **16**

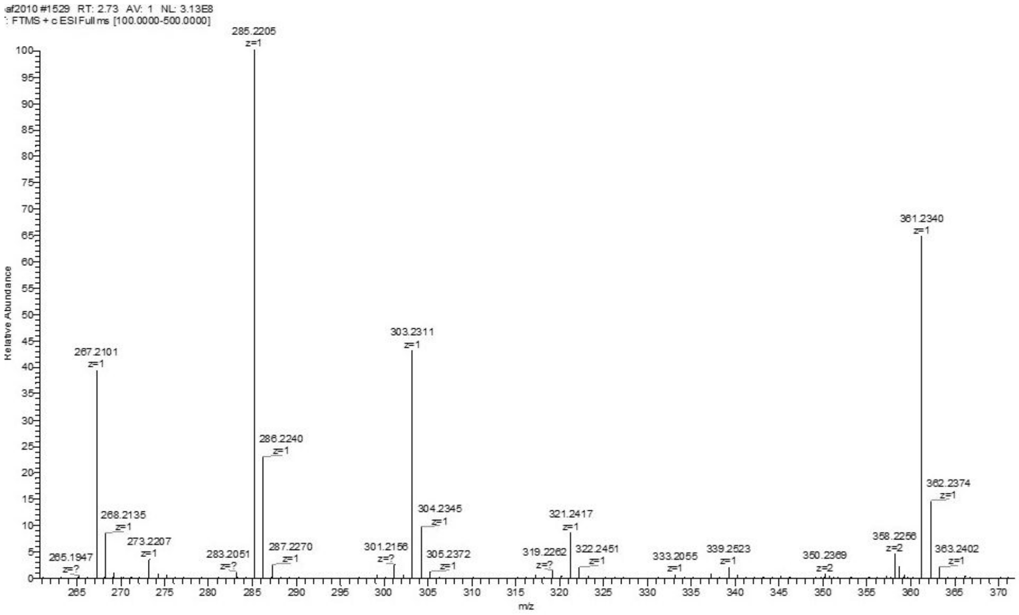

**Table S95**. $^{13}C$ experimental and calculated NMR chemical shifts for **1a-d**, with [a]$|\Delta\delta|(^{13}C)$ and [b]MAE values.

| | $\delta_{calc}$ ($^{13}C$), ppm | | | | $\delta_{exp}$ ($^{13}C$), ppm | $|\Delta\delta|$ ($^{13}C$), ppm[a] | | | |
|---|---|---|---|---|---|---|---|---|---|
| | **1a** | **1b** | **1c** | **1d** | **experimental** | **1a** | **1b** | **1c** | **1d** |
| **Position** | *8R*,12S*, 16R** | *8R*,12S*, 16S** | *8S*,12R*, 16R** | *8S*,12R*, 16S** | | *8R*,12S*, 16R** | *8R*,12S*, 16S** | *8S*,12R*, 16R** | *8S*,12R*, 16S** |
| 1 | 39.47 | 39.34 | 40.33 | 39.82 | 39.4 | 0.07 | 0.06 | 0.93 | 0.42 |
| 2 | 19.72 | 19.67 | 19.79 | 19.93 | 17.8 | 1.92 | 1.87 | 1.99 | 2.13 |
| 3 | 40.15 | 39.78 | 39.97 | 41.85 | 39.7 | 0.45 | 0.08 | 0.27 | 2.15 |
| 4 | 38.96 | 39.09 | 39.03 | 39.35 | 38.8 | 0.16 | 0.29 | 0.23 | 0.55 |
| 5 | 60.56 | 60.75 | 60.07 | 58.25 | 61.5 | 0.94 | 0.75 | 1.43 | 3.25 |
| 6 | 69.18 | 69.00 | 69.12 | 66.34 | 68.2 | 0.98 | 0.80 | 0.92 | 1.86 |
| 7 | 49.79 | 49.93 | 50.54 | 50.10 | 49.4 | 0.39 | 0.53 | 1.14 | 0.70 |
| 8 | 35.57 | 35.76 | 35.22 | 34.75 | 39.3 | 3.73 | 3.54 | 4.08 | 4.55 |
| 9 | 51.56 | 52.29 | 50.78 | 51.08 | 52.0 | 0.44 | 0.29 | 1.22 | 0.92 |
| 10 | 41.40 | 41.64 | 41.98 | 40.75 | 38.3 | 3.10 | 3.34 | 3.68 | 2.45 |
| 11 | 26.80 | 25.83 | 25.02 | 26.28 | 22.3 | 4.50 | 3.53 | 2.72 | 3.98 |
| 12 | 36.05 | 35.84 | 36.31 | 35.58 | 32.8 | 3.25 | 3.04 | 3.51 | 2.78 |
| 13 | 23.80 | 24.94 | 23.32 | 22.93 | 25.0 | 1.20 | 0.06 | 1.68 | 2.07 |
| 14 | 29.64 | 29.30 | 38.67 | 38.97 | 29.6 | 0.04 | 0.30 | 9.07 | 9.37 |
| 15 | 54.48 | 54.25 | 45.83 | 46.07 | 52.5 | 1.98 | 1.75 | 6.67 | 6.43 |
| 16 | 74.47 | 73.92 | 74.29 | 74.42 | 75.0 | 0.53 | 1.08 | 0.71 | 0.58 |
| 17 | 69.31 | 68.90 | 69.22 | 68.30 | 68.7 | 0.61 | 0.20 | 0.52 | 0.40 |
| 18 | 32.55 | 32.27 | 33.18 | 30.18 | 31.0 | 1.55 | 1.27 | 2.18 | 0.82 |
| 19 | 68.37 | 67.86 | 68.92 | 69.46 | 65.6 | 2.77 | 2.26 | 3.32 | 3.86 |
| 20 | 15.74 | 15.73 | 17.15 | 16.94 | 16.0 | 0.26 | 0.27 | 1.15 | 0.94 |
| **MAE**[b] | | | | | | 1.44 | **1.27** | 2.37 | 2.51 |

[a] **$|\Delta\delta|(^{13}C)$** = $|\delta_{exp} - \delta_{calc}|$ ($^{13}C$), ppm: absolute differences for experimental versus calculated $^{13}C$ NMR chemical shifts; [b] **MAE** = $\Sigma[|(\delta_{exp} - \delta_{calcd})|]/n$, summation through n of the absolute error values (difference of the absolute values between corresponding experimental and $^{13}C$ chemical shifts), normalized to the number of the chemical shifts.

**Table S96**. $^1$H experimental and calculated NMR chemical shifts for **1a-d**, with $^a|\Delta\delta|(^1H)$ and $^b$MAE values.

| | δcalc ($^1$H), ppm | | | | $\delta_{exp}$ ($^1$H), ppm | \|Δδ\| ($^1$H), ppm[a] | | | |
|---|---|---|---|---|---|---|---|---|---|
| | **1a** | **1b** | **1c** | **1d** | **experimental** | **1a** | **1b** | **1c** | **1d** |
| **Position** | *8R*,12S*, 16R** | *8R*,12S*, 16S** | *8S*,12R*, 16R** | *8S*,12R*, 16S** | | *8R*,12S*, 16R** | *8R*,12S*, 16S** | *8S*,12R*, 16R** | *8S*,12R*, 16S** |
| 1 | 1.49 | 1.54 | 1.61 | 1.44 | 1.54 | 0.05 | 0.00 | 0.07 | 0.10 |
| | 0.78 | 0.81 | 0.89 | 0.80 | 0.91 | 0.13 | 0.10 | 0.02 | 0.11 |
| 2 | 1.65 | 1.67 | 1.65 | 1.48 | 1.56 | 0.09 | 0.11 | 0.09 | 0.08 |
| | 1.19 | 1.21 | 1.21 | 1.15 | 1.34 | 0.37 | 0.35 | 0.35 | 0.41 |
| 3 | 1.10 | 1.08 | 1.10 | 1.17 | 1.12 | 0.02 | 0.04 | 0.02 | 0.05 |
| | 1.20 | 1.29 | 1.22 | 1.40 | 1.56 | 0.36 | 0.27 | 0.34 | 0.16 |
| 5 | 0.93 | 0.95 | 1.00 | 0.93 | 1.03 | 0.1 | 0.08 | 0.03 | 0.10 |
| 6 | 4.47 | 4.40 | 4.41 | 4.22 | 4.05 | 0.42 | 0.35 | 0.36 | 0.17 |
| 7 | 0.93 | 0.93 | 0.96 | 1.19 | 1.2 | 0.27 | 0.27 | 0.24 | 0.01 |
| | 1.63 | 1.62 | 1.69 | 1.66 | 1.67 | 0.04 | 0.05 | 0.02 | 0.01 |
| 9 | 0.99 | 1.10 | 1.14 | 1.14 | 1.02 | 0.03 | 0.08 | 0.12 | 0.12 |
| 11 | 1.20 | 1.16 | 1.67 | 1.24 | 1.31 | 0.11 | 0.15 | 0.36 | 0.07 |
| | 1.48 | 1.83 | 1.35 | 1.38 | 2.09 | 0.61 | 0.26 | 0.74 | 0.71 |
| 12 | 1.41 | 1.40 | 1.38 | 1.36 | 1.82 | 0.41 | 0.42 | 0.44 | 0.46 |
| 13 | 1.34 | 1.35 | 1.44 | 1.82 | 1.26 | 0.08 | 0.09 | 0.18 | 0.56 |
| | 1.85 | 1.50 | 1.38 | 1.34 | 1.65 | 0.2 | 0.15 | 0.27 | 0.31 |
| 14 | 1.04 | 0.92 | 1.02 | 1.20 | 1.33 | 0.29 | 0.41 | 0.31 | 0.13 |
| | 1.95 | 1.83 | 1.16 | 1.29 | 1.9 | 0.05 | 0.07 | 0.74 | 0.61 |
| 15 | 1.79 | 0.89 | 1.72 | 0.91 | 1.08 | 0.71 | 0.19 | 0.64 | 0.17 |
| | 1.04 | 1.85 | 1.78 | 2.60 | 1.26 | 0.22 | 0.59 | 0.52 | 1.34 |
| 17 | 3.39 | 3.41 | 3.34 | 3.57 | 3.37 | 0.02 | 0.04 | 0.03 | 0.20 |
| | 3.48 | 3.45 | 3.41 | 3.36 | 3.5 | 0.02 | 0.05 | 0.09 | 0.14 |
| 18 | 1.12 | 1.12 | 1.13 | 1.14 | 1.19 | 0.07 | 0.07 | 0.06 | 0.05 |
| 19 | 3.30 | 3.40 | 3.35 | 3.44 | 3.46 | 0.16 | 0.06 | 0.11 | 0.02 |
| | 4.19 | 4.17 | 4.21 | 4.40 | 4 | 0.19 | 0.17 | 0.21 | 0.40 |
| 20 | 1.21 | 1.19 | 1.23 | 1.09 | 1.09 | 0.12 | 0.10 | 0.14 | 0.00 |
| **MAE**[b] | | | | | | 0.20 | **0.17** | 0.25 | 0.25 |

[a] **$|\Delta\delta|(^1H)$** = |δexp –δcalc| ($^1$H), ppm: absolute differences for experimental versus calculated $^1$H NMR chemical shifts; [b] **MAE** = $\Sigma[|(\delta_{exp} - \delta_{calcd})|]/n$, summation through n of the absolute error values (difference of the absolute values between corresponding experimental and $^1$H chemical shifts), normalized to the number of the chemical shifts.

**Table S97**. $^{13}$C experimental and calculated NMR chemical shifts for **4a-b**, with $^{a}|\Delta\delta|(^{13}C)$ and $^{b}$MAE values.

| | $\delta_{calc}$ ($^{13}$C), ppm | | $\delta_{exp}$ ($^{13}$C), ppm | $\|\Delta\delta\|$ ($^{13}$C), ppm[a] | |
|---|---|---|---|---|---|
| | **4a** | **4b** | **experimental** | **4a** | **4b** |
| **Position** | *8R*,13S** | *8S*,13R** | | *8R*,13S** | *8S*,13R** |
| 1 | 53.47 | 55.62 | 53.80 | 0.33 | 1.82 |
| 2 | 209.89 | 209.77 | 215.60 | 5.71 | 5.83 |
| 3 | 46.36 | 46.39 | 44.80 | 1.56 | 1.59 |
| 4 | 48.73 | 48.46 | 46.80 | 1.93 | 1.66 |
| 5 | 53.78 | 53.08 | 47.60 | 6.18 | 5.48 |
| 6 | 22.66 | 22.45 | 20.60 | 2.06 | 1.85 |
| 7 | 37.33 | 39.36 | 36.90 | 0.43 | 2.46 |
| 8 | 49.72 | 50.92 | 43.30 | 6.42 | 7.62 |
| 9 | 52.66 | 48.00 | 52.40 | 0.26 | 4.40 |
| 10 | 44.59 | 45.66 | 42.20 | 2.39 | 3.46 |
| 11 | 22.53 | 22.37 | 20.00 | 2.53 | 2.37 |
| 12 | 33.16 | 34.10 | 33.00 | 0.16 | 1.10 |
| 13 | 45.01 | 46.97 | 48.90 | 3.89 | 1.93 |
| 14 | 60.41 | 50.17 | 61.30 | 0.89 | 11.13 |
| 15 | 132.65 | 139.20 | 134.90 | 2.25 | 4.30 |
| 16 | 135.37 | 136.65 | 137.00 | 1.63 | 0.35 |
| 17 | 25.54 | 25.73 | 24.80 | 0.74 | 0.93 |
| 18 | 73.23 | 73.77 | 66.60 | 6.63 | 7.17 |
| 19 | 65.89 | 66.25 | 63.40 | 2.49 | 2.85 |
| 20 | 18.29 | 20.99 | 16.80 | 1.49 | 4.19 |
| **MAE[b]** | | | | **2.50** | 3.63 |

[a] $\mathbf{|\Delta\delta|(^{13}C)} = |\delta_{exp} - \delta_{calc}|$ ($^{13}$C), ppm: absolute differences for experimental versus calculated $^{13}$C NMR chemical shifts; [b] **MAE** = $\Sigma[|(\delta_{exp} - \delta_{calcd})|]/n$, summation through n of the absolute error values (difference of the absolute values between corresponding experimental and $^{13}$C chemical shifts), normalized to the number of the chemical shifts.

**Table S98**. $^1$H experimental and calculated NMR chemical shifts for **4a-b**, with [a]|Δδ|($^1$H) and [b]MAE values.

| | δcalc ($^1$H), ppm | | $\delta_{exp}$ ($^1$H), ppm | \|Δδ\| ($^1$H), ppm[a] | |
|---|---|---|---|---|---|
| | **4a** | **4b** | **experimental** | **4a** | **4b** |
| **Position** | *8R*,13S** | *8S*,13R** | | *8R*,13S** | *8S*,13R** |
| 1 | 2.16 | 2.42 | 2.26 | 0.10 | 0.16 |
| | 2.01 | 1.89 | 2.12 | 0.11 | 0.23 |
| 3 | 2.19 | 2.12 | 2.52 | 0.33 | 0.40 |
| | 1.93 | 1.97 | 2.44 | 0.51 | 0.51 |
| 5 | 1.58 | 1.47 | 1.98 | 0.40 | 0.51 |
| 6 | 2.28 | 2.20 | 1.74 | 0.54 | 0.46 |
| | 1.72 | 1.77 | 1.5 | 0.22 | 0.27 |
| 7 | 1.41 | 1.64 | 1.48 | 0.07 | 0.16 |
| | 1.62 | 1.56 | 1.66 | 0.04 | 0.10 |
| 9 | 1.23 | 1.20 | 1.36 | 0.13 | 0.16 |
| 11 | 1.46 | 1.49 | 1.56 | 0.10 | 0.07 |
| | 1.38 | 1.75 | 1.5 | 0.12 | 0.25 |
| 12 | 1.24 | 1.29 | 1.37 | 0.13 | 0.08 |
| | 1.29 | 1.37 | 1.37 | 0.08 | 0.00 |
| 14 | 1.01 | 1.24 | 1.12 | 0.11 | 0.12 |
| | 1.58 | 2.00 | 1.51 | 0.07 | 0.49 |
| 15 | 6.22 | 5.89 | 5.74 | 0.48 | 0.15 |
| 16 | 5.99 | 6.03 | 5.5 | 0.49 | 0.53 |
| 17 | 0.95 | 1.03 | 1.04 | 0.09 | 0.01 |
| 18 | 3.61 | 3.73 | 3.6 | 0.01 | 0.13 |
| | 3.54 | 3.58 | 3.47 | 0.07 | 0.11 |
| 19 | 3.90 | 3.81 | 3.58 | 0.32 | 0.23 |
| | 3.97 | 3.88 | 3.48 | 0.49 | 0.40 |
| 20 | 0.80 | 1.16 | 0.84 | 0.04 | 0.32 |
| **MAE[b]** | | | | **0.21** | 0.24 |

[a] **|Δδ|($^1$H)** = |δexp –δcalc| ($^1$H), ppm: absolute differences for experimental versus calculated $^1$H NMR chemical shifts; [b] **MAE** = Σ[|($\delta_{exp}$ – $\delta_{calcd}$)|]/n, summation through n of the absolute error values (difference of the absolute values between corresponding experimental and $^1$H chemical shifts), normalized to the number of the chemical shifts.

**Table S99**. $^{13}$C experimental and calculated NMR chemical shifts for **6a-b**, with $^{a}|\Delta\delta|(^{13}C)$ and $^{b}$MAE values.

| | $\delta_{calc}$ ($^{13}$C), ppm | | $\delta_{exp}$ ($^{13}$C), ppm | $|\Delta\delta|$ ($^{13}$C), ppm$^{a}$ | |
|---|---|---|---|---|---|
| | **6a** | **6b** | **experimental** | **6a** | **6b** |
| **Position** | *8R*,13S** | *8S*,13R** | | *8R*,13S** | *8S*,13R** |
| 1 | 42.94 | 46.43 | 46.7 | 3.76 | 0.27 |
| 2 | 65.65 | 68.11 | 67.8 | 2.15 | 0.31 |
| 3 | 36.62 | 35.26 | 36.0 | 0.62 | 0.74 |
| 4 | 42.52 | 43.48 | 42.0 | 0.52 | 1.48 |
| 5 | 50.26 | 49.37 | 48.6 | 1.66 | 0.77 |
| 6 | 21.54 | 20.91 | 21.0 | 0.54 | 0.09 |
| 7 | 37.66 | 39.97 | 38.4 | 0.74 | 1.57 |
| 8 | 49.49 | 51.13 | 49.2 | 0.29 | 1.93 |
| 9 | 53.78 | 48.73 | 55.0 | 1.22 | 6.27 |
| 10 | 38.51 | 40.82 | 36.9 | 1.61 | 3.92 |
| 11 | 22.32 | 22.17 | 20.8 | 1.52 | 1.37 |
| 12 | 33.40 | 34.55 | 33.5 | 0.10 | 1.05 |
| 13 | 44.88 | 47.06 | 43.4 | 1.48 | 3.66 |
| 14 | 60.73 | 50.96 | 62.0 | 1.27 | 11.04 |
| 15 | 133.63 | 139.96 | 135.9 | 2.27 | 4.06 |
| 16 | 134.68 | 136.14 | 137.5 | 2.82 | 1.36 |
| 17 | 25.62 | 25.77 | 25.6 | 0.02 | 0.17 |
| 18 | 75.18 | 70.30 | 69.3 | 5.88 | 1.00 |
| 19 | 65.65 | 64.26 | 66.0 | 0.35 | 1.74 |
| 20 | 18.84 | 22.22 | 20.0 | 1.16 | 2.22 |
| **MAE$^{b}$** | | | | **1.50** | 2.25 |

$^{a}$ $\mathbf{|\Delta\delta|(^{13}C)}$ = $|\delta_{exp} - \delta_{calc}|$ ($^{13}$C), ppm: absolute differences for experimental versus calculated $^{13}$C NMR chemical shifts; $^{b}$ **MAE** = $\Sigma[|(\delta_{exp} - \delta_{calcd})|]/n$, summation through n of the absolute error values (difference of the absolute values between corresponding experimental and $^{13}$C chemical shifts), normalized to the number of the chemical shifts.

**Table S100**. $^1$H experimental and calculated NMR chemical shifts for **6a-b**, with $^a|\Delta\delta|(^1H)$ and $^b$MAE values.

| | δcalc ($^1$H), ppm | | $\delta_{exp}$ ($^1$H), ppm | \|Δδ\| ($^1$H), ppm[a] | |
|---|---|---|---|---|---|
| | **6a** | **6b** | | **6a** | **6b** |
| **Position** | *8R*,13S** | *8S*,13R** | | *8R*,13S** | *8S*,13R** |
| 1 | 1.87 | 1.93 | 1.67 | 0.20 | 0.26 |
| | 0.85 | 0.65 | 1.40 | 0.55 | 0.75 |
| 2 | 4.14 | 4.22 | 4.11 | 0.03 | 0.11 |
| 3 | 1.09 | 1.03 | 1.67 | 0.58 | 0.64 |
| | 2.69 | 2.89 | 1.76 | 0.93 | 1.13 |
| 5 | 1.08 | 1.10 | 1.34 | 0.26 | 0.24 |
| 6 | 1.34 | 1.74 | 1.65 | 0.31 | 0.09 |
| | 1.58 | 1.49 | 1.53 | 0.05 | 0.04 |
| 7 | 1.22 | 1.58 | 1.37 | 0.15 | 0.21 |
| | 1.54 | 1.51 | 1.60 | 0.06 | 0.09 |
| 9 | 0.94 | 0.94 | 1.07 | 0.13 | 0.13 |
| 11 | 1.48 | 1.63 | 1.78 | 0.30 | 0.15 |
| | 1.45 | 1.69 | 1.48 | 0.03 | 0.21 |
| 12 | 1.21 | 1.28 | 1.28 | 0.07 | 0.00 |
| | 1.29 | 1.36 | 1.36 | 0.07 | 0.00 |
| 14 | 0.93 | 1.18 | 1.06 | 0.13 | 0.12 |
| | 1.49 | 2.04 | 1.44 | 0.05 | 0.60 |
| 15 | 6.16 | 5.84 | 5.75 | 0.41 | 0.09 |
| 16 | 5.93 | 5.99 | 5.48 | 0.45 | 0.51 |
| 17 | 0.92 | 1.00 | 1.00 | 0.08 | 0.00 |
| 18 | 4.01 | 3.23 | 3.51 | 0.50 | 0.28 |
| | 3.43 | 3.61 | 3.55 | 0.12 | 0.06 |
| 19 | 4.41 | 4.34 | 3.91 | 0.50 | 0.43 |
| | 3.66 | 3.31 | 3.66 | 0.00 | 0.35 |
| 20 | 1.31 | 1.51 | 1.06 | 0.25 | 0.45 |
| **MAE[b]** | | | | **0.25** | 0.28 |

[a] **$|\Delta\delta|(^1H)$** = |δexp –δcalc| ($^1$H), ppm: absolute differences for experimental versus calculated $^1$H NMR chemical shifts; [b] **MAE** = $\Sigma[|(\delta_{exp} - \delta_{calcd})|]/n$, summation through n of the absolute error values (difference of the absolute values between corresponding experimental and $^1$H chemical shifts), normalized to the number of the chemical shifts.

**Table S101**. $^{13}C$ experimental and calculated NMR chemical shifts for **7a-b**, with [a]$|\Delta\delta|(^{13}C)$ and [b]MAE values.

| | $\delta_{calc}$ ($^{13}C$), ppm | | $\delta_{exp}$ ($^{13}C$), ppm | $|\Delta\delta|$ ($^{13}C$), ppm[a] | |
|---|---|---|---|---|---|
| | **7a** | **7b** | **experimental** | **7a** | **7b** |
| **Position** | *8R*,13R** | *8S*,13S** | | *8R*,13R** | *8S*,13S** |
| 1 | 45.81 | 45.08 | 46.4 | 0.59 | 1.32 |
| 2 | 68.09 | 68.00 | 68.0 | 0.09 | 0.00 |
| 3 | 39.42 | 42.09 | 41.0 | 1.58 | 1.09 |
| 4 | 38.60 | 38.68 | 38.5 | 0.10 | 0.18 |
| 5 | 47.28 | 47.49 | 48.0 | 0.72 | 0.51 |
| 6 | 21.36 | 20.79 | 21.2 | 0.16 | 0.41 |
| 7 | 37.36 | 39.30 | 37.8 | 0.44 | 1.50 |
| 8 | 49.29 | 51.22 | 51.2 | 1.91 | 0.02 |
| 9 | 53.85 | 48.68 | 55.4 | 1.55 | 6.72 |
| 10 | 38.97 | 40.88 | 38.3 | 0.67 | 2.58 |
| 11 | 22.14 | 21.92 | 21.1 | 1.04 | 0.82 |
| 12 | 28.28 | 29.73 | 28.9 | 0.62 | 0.83 |
| 13 | 50.65 | 53.29 | 49.7 | 0.95 | 3.59 |
| 14 | 56.41 | 45.99 | 57.2 | 0.79 | 11.21 |
| 15 | 134.23 | 140.90 | 137.2 | 2.98 | 3.70 |
| 16 | 131.31 | 132.72 | 133.5 | 2.19 | 0.78 |
| 17 | 68.83 | 69.74 | 69.0 | 0.17 | 0.74 |
| 18 | 71.06 | 71.11 | 71.6 | 0.54 | 0.49 |
| 19 | 21.32 | 20.62 | 20.7 | 0.62 | 0.08 |
| 20 | 19.33 | 22.02 | 19.5 | 0.17 | 2.52 |
| **MAE[b]** | | | | **0.89** | 1.96 |

[a] $\mathbf{|\Delta\delta|(^{13}C)} = |\delta_{exp} - \delta_{calc}|$ ($^{13}C$), ppm: absolute differences for experimental versus calculated $^{13}C$ NMR chemical shifts; [b] **MAE** = $\Sigma[|(\delta_{exp} - \delta_{calcd})|]/n$, summation through n of the absolute error values (difference of the absolute values between corresponding experimental and $^{13}C$ chemical shifts), normalized to the number of the chemical shifts.

**Table S102**. $^1H$ experimental and calculated NMR chemical shifts for **7a-b**, with [a]$|\Delta\delta|(^1H)$ and [b]MAE values.

| | δcalc ($^1H$), ppm | | $\delta_{exp}$ ($^1H$), ppm | $|\Delta\delta|(^1H)$, ppm[a] | |
|---|---|---|---|---|---|
| | **7a** | **7b** | **experimental** | | |
| **Position** | *8R*,13R** | *8S*,13S** | | *8R*,13R** | *8S*,13S** |
| 1 | 1.65 | 1.94 | 1.69 | 0.04 | 0.25 |
| | 1.16 | 1.08 | 1.42 | 0.26 | 0.34 |
| 2 | 4.25 | 4.26 | 4.13 | 0.12 | 0.13 |
| 3 | 1.80 | 1.82 | 1.81 | 0.01 | 0.01 |
| | 1.41 | 1.26 | 1.47 | 0.06 | 0.21 |
| 5 | 1.29 | 1.18 | 1.28 | 0.01 | 0.10 |
| 6 | 1.43 | 1.41 | 1.49 | 0.06 | 0.08 |
| | 1.69 | 1.59 | 1.54 | 0.15 | 0.05 |
| 7 | 1.32 | 1.62 | 1.47 | 0.15 | 0.15 |
| | 1.60 | 1.52 | 1.66 | 0.06 | 0.14 |
| 9 | 1.01 | 0.99 | 1.12 | 0.11 | 0.13 |
| 11 | 1.49 | 1.73 | 1.45 | 0.04 | 0.28 |
| | 1.56 | 1.69 | 1.66 | 0.10 | 0.03 |
| 12 | 1.09 | 1.23 | 1.33 | 0.24 | 0.10 |
| | 1.44 | 1.79 | 1.44 | 0.00 | 0.35 |
| 14 | 0.83 | 1.88 | 1.03 | 0.20 | 0.85 |
| | 1.37 | 1.36 | 1.59 | 0.22 | 0.23 |
| 15 | 6.31 | 5.91 | 5.84 | 0.47 | 0.07 |
| 16 | 6.19 | 5.91 | 5.63 | 0.56 | 0.28 |
| 17 | 3.49 | 3.53 | 3.45 | 0.04 | 0.08 |
| | 3.30 | 3.57 | 3.42 | 0.12 | 0.15 |
| 18 | 2.84 | 2.88 | 3.09 | 0.25 | 0.21 |
| | 3.51 | 3.48 | 3.37 | 0.14 | 0.11 |
| 19 | 0.96 | 0.94 | 0.99 | 0.03 | 0.05 |
| 20 | 1.25 | 1.53 | 1.10 | 0.15 | 0.43 |
| **MAE**[b] | | | | **0.14** | 0.19 |

[a] $|\Delta\delta|(^1H)$ = |δexp –δcalc| ($^1H$), ppm: absolute differences for experimental versus calculated $^1H$ NMR chemical shifts; [b] **MAE** = $\Sigma[|(\delta_{exp} - \delta_{calcd})|]/n$, summation through n of the absolute error values (difference of the absolute values between corresponding experimental and $^1H$ chemical shifts), normalized to the number of the chemical shifts.

**Table S103.** $^{13}C/^{1}H$ MAE (ppm) Values and DP4+ Data Reported for All the Possible Relative Stereoisomers for Compounds **1**, **4**, **6** and **7**.

| | # of possible relative stereoisomers | Stereoisomer | Relative configuration | $^{13}C$ MAE (ppm)[a] | $^{1}H$ MAE (ppm)[b] | DP4+ probability[c] |
|---|---|---|---|---|---|---|
| **1** | 4 | **1a** | *8R*,12S*,16R** | 1.44 | 0.20 | 0.00% |
| | | **1b** | ***8R*,12S*,16S**** | **1.27** | **0.17** | **100.00%** |
| | | **1c** | *8S*,12R*,16R** | 2.37 | 0.25 | 0.00% |
| | | **1d** | *8S*,12R*,16S** | 2.51 | 0.25 | 0.00% |
| **4** | 2 | **4a** | *8R*,13S** | **2.50** | **0.21** | **100.00%** |
| | | **4b** | *8S*,13R** | 3.63 | 0.24 | 0.00% |
| **6** | 2 | **6a** | *8R*,13S** | **1.50** | **0.25** | **100.00%** |
| | | **6b** | *8S*,13R** | 2.25 | 0.28 | 0.00% |
| **7** | 2 | **7a** | *8R*,13R** | **0.89** | **0.14** | **100.00%** |
| | | **7b** | *8S*,13S** | 1.96 | 0.19 | 0.00% |

[a] **$^{13}$C MAE** = $\Sigma[|(\delta_{exp} - \delta_{calcd})|]/n$, summation through n of the absolute error values (difference of the absolute values between corresponding experimental and $^{13}$C chemical shifts), normalized to the number of the chemical shifts. Chemical shift data reported were produced using the "multi standard" approach, using TMS as reference compound for $sp^3$ $^{13}$C atoms, and benzene for $sp^2$ $^{13}$C atoms; the related data are reported in Tables S1, S3, S5 and S7 Supporting Information.

[b] **$^{1}$H MAE** = $\Sigma[|(\delta_{exp} - \delta_{calcd})|]/n$, summation through n of the absolute error values (difference of the absolute values between corresponding experimental and $^{1}$H chemical shifts), normalized to the number of the chemical shifts. Chemical shift data reported were produced using the "multi standard" approach, using TMS as reference compound for $sp^3$ $^{1}$H atoms, and benzene for $sp^2$ $^{1}$H atoms; the related data are reported in in Tables S2, S4, S6 and S8 Supporting Information.

[c] **DP4+ probabilities** related to the set of data reported in Tables S1-S8 ($^{13}$C and $^{1}$H chemical shift set of data). This set of data was produced using only TMS as reference compound, and then $sp^3$ and $sp^2$ atoms were differently treated following the "multi-standard" approach flagging the latter in the DP4+ Excel file.
